# Contributions of Emotion Regulation and Brain Structure and Function to Adolescent Internalizing Problems and Stress Vulnerability During the COVID-19 Pandemic: A Longitudinal Study

**DOI:** 10.1016/j.bpsgos.2021.06.001

**Published:** 2021-06-12

**Authors:** David G. Weissman, Alexandra M. Rodman, Maya L. Rosen, Steven Kasparek, Makeda Mayes, Margaret A. Sheridan, Lilliana J. Lengua, Andrew N. Meltzoff, Katie A. McLaughlin

**Affiliations:** a Department of Psychology, Harvard University, Cambridge, Massachusetts; b Department of Psychology, University of Washington, Seattle, Washington; c Department of Psychology and Neuroscience, University of North Carolina at Chapel Hill, Chapel Hill, North Carolina

**Keywords:** Amygdala, Cognitive reappraisal, Expressive suppression, Hippocampus, Psychopathology, Rumination

## Abstract

**Background:**

Adolescence is a period of increased vulnerability for internalizing problems, particularly following stressful life events. We examined how emotion regulation and brain structure and function were associated with internalizing problems during the COVID-19 pandemic and moderated the association between pandemic-related stressors and internalizing problems.

**Methods:**

Data are from a longitudinal sample (*N* = 145, age range, 10–15 years) strategically assessed at 3 crucial time points: before the COVID-19 pandemic, early during the stay-at-home order period, and again 6 months later. We examined associations of amygdala and hippocampal volume and amygdala activation during an emotional processing task before the pandemic, examined use of emotion regulation strategies before and during the pandemic, and examined pandemic-related stressors with internalizing problems.

**Results:**

Greater exposure to pandemic-related stressors was associated with higher internalizing problems both early and later in the COVID-19 pandemic. Youths who reported more frequent use of rumination before the pandemic and higher use of expressive suppression and lower use of cognitive reappraisal early in the pandemic had higher internalizing problems early in the pandemic. Higher left amygdala activation to neutral relative to fearful faces before the pandemic was associated with greater internalizing problems and a stronger link between pandemic-related stressors and internalizing problems early in the pandemic.

**Conclusions:**

Stressors related to the COVID-19 pandemic are strongly associated with adolescent internalizing problems, as are individual differences in emotional reactivity and regulation and their underlying neural mechanisms. Interventions that reduce pandemic-related stressors and foster adaptive emotion regulation skills may protect against adolescent psychopathology during this period of heightened exposure to stress.

Adolescence is a period of increased risk for internalizing problems (1,2). Exposure to stressful life events increases during adolescence, and adolescents experience heightened vulnerability to developing stress-related anxiety and depression symptoms (3, 4, 5, 6). The COVID-19 pandemic has produced dramatic societal changes that have resulted in increased exposure to numerous health, economic, and social stressors for adolescents. Internalizing problems have increased during the pandemic in both adolescents and adults (7, 8, 9, 10, 11). However, the degree of exposure to pandemic-related stressors varies widely (10,12,13), and risk for internalizing problems related to these stressors is unlikely to be uniform. Identifying characteristics that convey risk for or resilience to internalizing problems during the COVID-19 pandemic may help to generate targets for interventions to promote well-being during this period of heightened stress exposure. This longitudinal study investigated how individual differences in multiple domains of emotional processing—use of specific emotion regulation strategies, brain structure, and neural function during emotional processing—predicted vulnerability to stress-related internalizing problems during the COVID-19 pandemic.

Difficulties with emotion regulation, increased amygdala reactivity to threat, and smaller hippocampal and amygdala volume are among the psychological and neural mechanisms that may increase vulnerability for internalizing problems in children and adolescents in response to stressful life events (14, 15, 16). Use of maladaptive emotion regulation strategies, such as rumination and suppression, is associated with elevated risk for internalizing problems in longitudinal studies and meta-analyses of adults and adolescents (17, 18, 19, 20). In contrast, greater use of adaptive emotion regulation strategies, such as cognitive reappraisal (21), is less consistently associated with psychopathology than maladaptive strategies (17,22,23). However, more habitual and effective use of cognitive reappraisal has a protective moderating influence on internalizing problems among youths who experience stressful life events (24,25). In the context of the COVID-19 pandemic, adolescents who engage in more cognitive reappraisal might be expected to be more resilient to pandemic-related stressors, while adolescents who engage in more expressive suppression and rumination might be expected to be more vulnerable to stress-related increases in internalizing problems.

Heightened amygdala reactivity to threat-related cues, such as fearful or angry faces or scenes depicting violence, may reflect that potential threats have greater emotional salience, leading to more intense negative emotional experiences and greater mobilization of defensive responses (26, 27, 28). Heightened amygdala reactivity to potential threats is associated with depression and anxiety in adolescence, including in prospective studies (29,30). Moreover, the prospective association between stressful life events and depression is stronger among youths with higher amygdala reactivity to fearful and angry faces (15). Similar to the amygdala, the hippocampus is involved in the processing of emotionally salient stimuli and in regulating physiological responses to stress and negative emotion (31, 32, 33), in addition to the central role it plays in learning and memory (34). While the association between hippocampal and amygdala volume and internalizing problems is inconsistent in children and adolescents (35, 36, 37, 38, 39), several studies have found that lower hippocampal volume is associated with greater vulnerability to internalizing problems following stressful life events. For example, maternal aggression is associated with greater increases in depressive symptoms over time in early adolescents with smaller hippocampal volume (40), and stressful life events are associated with elevated anxiety only among participants with small hippocampal volume (41). Finally, the prospective association between stressful life events and depression symptoms is stronger among youths with reduced hippocampal and amygdala volume (16). In the context of the COVID-19 pandemic, adolescents with greater amygdala reactivity to threat cues and smaller amygdala and hippocampal volume before the pandemic might be expected to be more vulnerable to stress-related increases in internalizing problems.

The current longitudinal study examined whether emotion regulation strategy use, amygdala reactivity to threat, and hippocampal and amygdala volume contribute to vulnerability to internalizing problems in response to stressors related to the COVID-19 pandemic. We expected that greater use of rumination and expressive suppression and less frequent use of cognitive reappraisal would be associated with more internalizing problems and would magnify the association between pandemic-related stressors and internalizing problems. Similarly, we expected that greater amygdala reactivity to threat and smaller amygdala and hippocampal volume—measured before the pandemic—would be associated with greater internalizing problems and vulnerability to stress-related internalizing problems. Owing to the unprecedented nature and duration of the COVID-19 pandemic, we did not have specific hypotheses about how associations might vary between early and later in the pandemic. However, internalizing problems present before the pandemic were measured over 2 years earlier and with a different scale than during the pandemic. We therefore anticipated greater stability in internalizing problems from early to later in the pandemic than from before the pandemic to early in the pandemic. Thus, statistically, risk and protective factors would likely be more strongly associated with changes in internalizing problems early in the pandemic than later.

## Methods and Materials

### Participants

**Figure 1:**
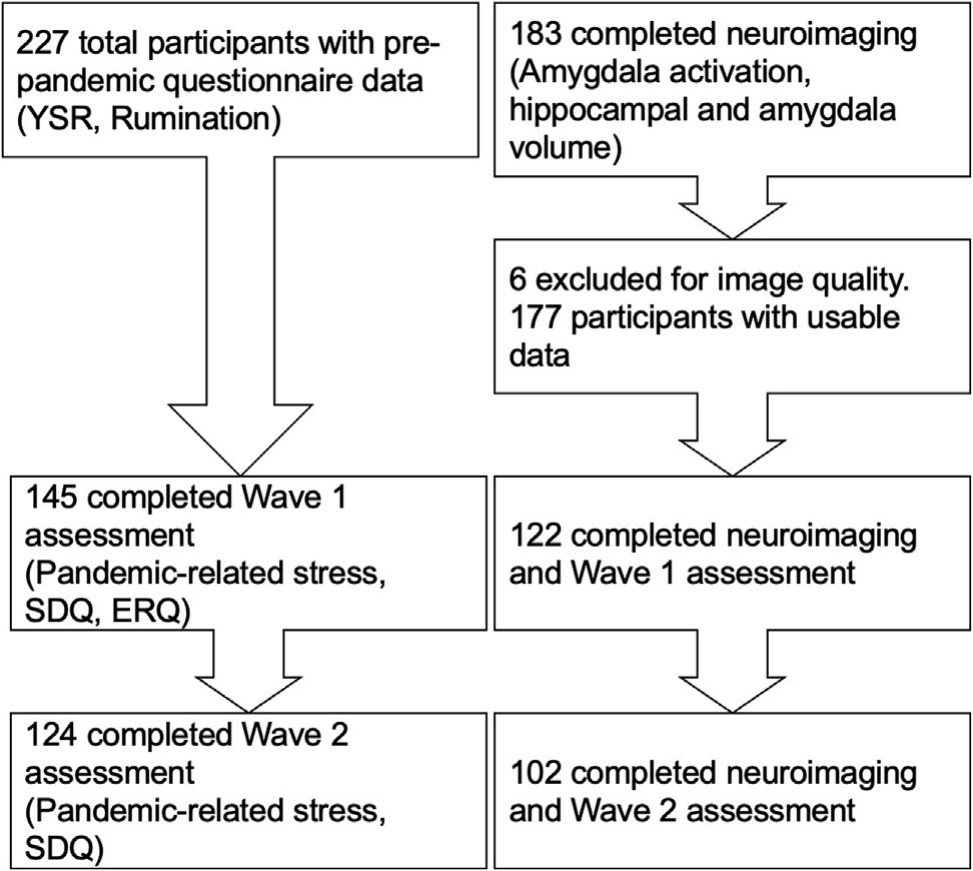
Participant recruitment and inclusion flowchart. Flowchart depicts recruitment and participation at each wave of data collection. ERQ, Emotion Regulation Questionnaire; SDQ, Strengths and Difficulties Questionnaire; YSR, Youth Self-Report.

Participants were drawn from a longitudinal study of children followed from age 3 and a caregiver, with the initial aim of studying the development of self-regulation in childhood (42). At age 11 to 12 years, between June 2017 and October 2018, before the pandemic, participants completed a baseline evaluation as part of a study examining mechanisms linking adverse childhood experiences with psychopathology (43). Participants were excluded from the study based on the following criteria: IQ < 80, active substance dependence, psychosis, presence of pervasive developmental disorders (e.g., autism), and psychotropic medication use. We conducted two assessments of this sample during the COVID-19 pandemic. Youths and a caregiver completed questionnaires online between April and May 2020 (wave 1) and between November 2020 and January 2021 (wave 2). See Figure 1 for a flowchart of participant recruitment and inclusion. Participant race/ethnicity is summarized in Table S1. Participants who had complete data at each wave did not differ from participants missing data on any variable included in study analyses.

### Measures

See the Supplement for greater details on all measures, including psychometrics.

#### Rumination (Prepandemic Baseline)

The 13-item rumination subscale of the Children’s Response Styles Questionnaire (44) was used to assess use of rumination.

#### Pandemic-Related Stressors (Wave 1 and Wave 2)

We developed a set of questions to assess pandemic-related stressors that was administered to children and caregivers. Stressors were coded as present if they were endorsed by either the youth or their parent and were summed, using a cumulative risk approach (45). See the Supplement and Table S2 for details. The complete scale can be found at https://osf.io/drqku/.

#### Cognitive Reappraisal and Expressive Suppression (Wave 1)

The Emotion Regulation Questionnaire (21) is a 10-item self-report questionnaire assessing the tendency to use cognitive reappraisal and expressive suppression. Of the 10 items, 6 pertain to cognitive reappraisal and 4 pertain to expressive suppression.

#### Internalizing Problems (Prepandemic Baseline, Wave 1, and Wave 2)

Internalizing problems at the prepandemic baseline were assessed based on child report on the Youth Self-Report (46). At the wave 1 and wave 2 COVID-19 follow-up assessments, adolescents completed the Strengths and Difficulties Questionnaire, a 25-item scale composed of 5 subscales with 5 items each (47,48). To make the scales comparable, internalizing scores were scaled to values between 0 and 1 (i.e., proportion of maximum scaling) (49).

### Emotional Processing Functional Magnetic Resonance Imaging Task (Prepandemic Baseline)

The emotional processing functional magnetic resonance imaging (fMRI) task consisted of 2 runs of 9 blocks, each lasting 18 seconds, during which participants passively viewed neutral, fearful, and scrambled face stimuli. Faces were drawn from the NimStim stimulus set (50). The calm faces from this dataset were used as neutral expressions, as these expressions are potentially less emotionally evocative than neutral faces (50), which can be perceived as negatively valenced (50). Further details can be found in the Supplement.

### MRI Acquisition and Preprocessing

MRI was performed on a 3T Achieva (Philips) scanner at the University of Washington Integrated Brain Imaging Center (repetition time = 2 seconds, voxel size = 3 mm^3^ for the functional scan). Preprocessing and statistical analysis of fMRI data were performed for the purposes of extracting activation to fearful versus neutral faces from left and right amygdala regions of interest. Further details can be found in the Supplement.

### Structural MRI Processing

Measures of hippocampal and amygdala volume and total intracranial volume were obtained using automatic segmentation in FreeSurfer 5.3 (https://surfer.nmr.mgh.harvard.edu/). Following prior work (16), right and left volumes were summed to create bilateral hippocampal and amygdala volume measures. To keep variables on a similar scale for regression analyses, subcortical volumes were divided by 1000, and intracranial volume was divided by 1,000,000. Further details can be found in the Supplement.

### Procedures

All study procedures were approved by the Institutional Review Board at Harvard University. Habitual use of rumination, internalizing problems, and measures of brain structure and function were assessed at the prepandemic baseline. Pandemic-related stressors, use of cognitive reappraisal and expressive suppression, and internalizing problems were assessed at each of the wave 1 and wave 2 follow-up assessments during the pandemic. Legal guardians provided informed consent, and youths provided assent via electronic signature obtained using Qualtrics (Provo, UT). Once consent and assent was obtained for the pandemic follow-up assessments, parents and children were separately provided with a link to surveys on RedCap (https://www.project-redcap.org/) and asked to complete them. If children had trouble completing the surveys on their own, an experimenter called via phone or video chat and read the questions aloud to the child and recorded their responses.

### Analyses

All analyses were conducted in R version 4.0.3 (R Foundation for Statistical Computing) (51). We used a paired sample *t* test to evaluate change in internalizing problems from the pandemic wave 1 to wave 2 follow-up. The associations between pandemic-related stressors and internalizing problems at the wave 1 and wave 2 follow-ups were evaluated using multiple linear regression, controlling for internalizing problems at the previous assessment, sex, and age.

**Figure 2:**
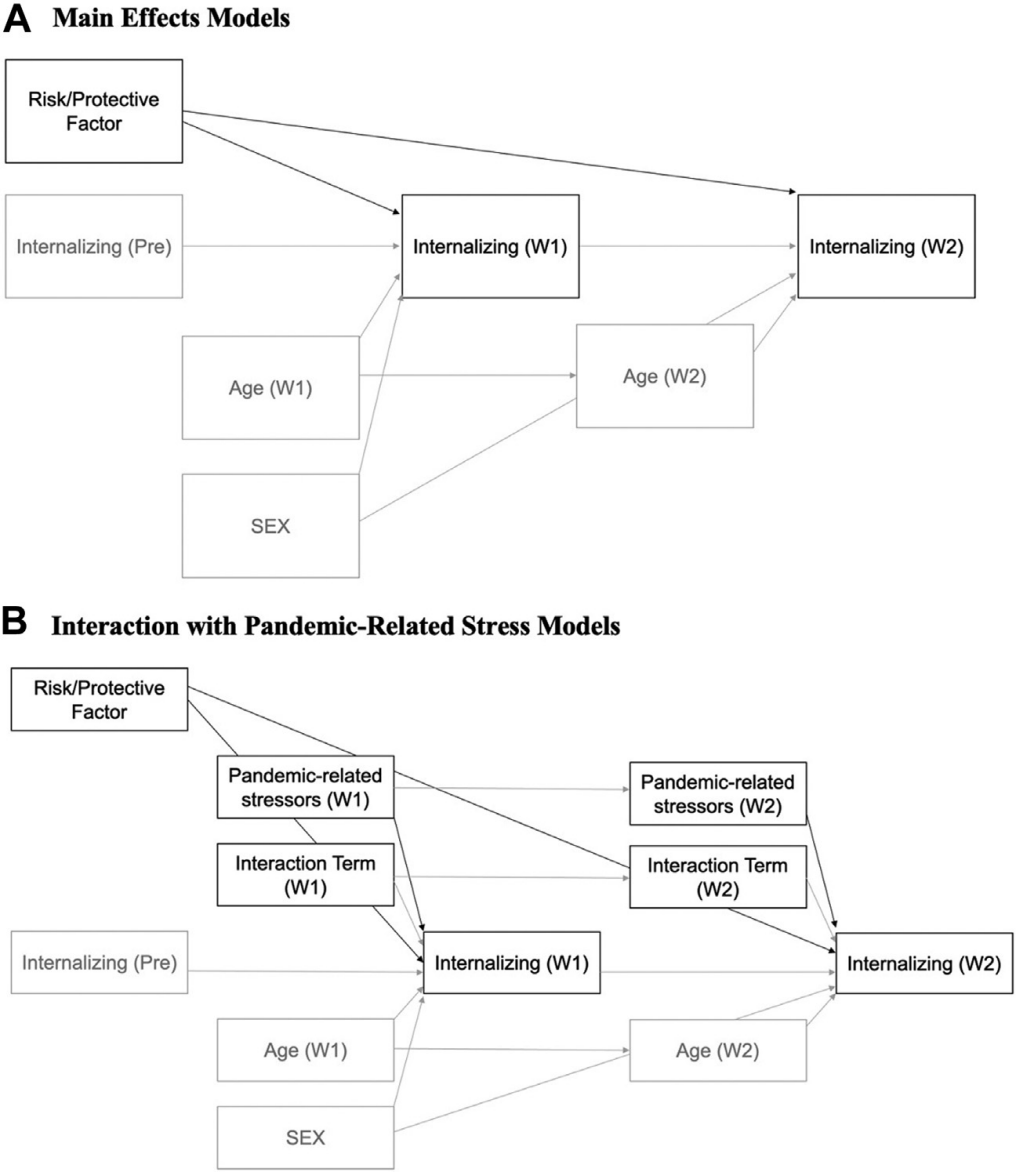
Longitudinal path models. Diagrams represent the path models used to test study hypotheses. Covariances were freely estimated. Models testing associations with hippocampal and amygdala volume also included paths from total intracranial volume to internalizing at wave 1 (W1) and wave 2 (W2) and covariances with the other predictors. Interaction Term refers to the product of risk/protective factor and pandemic-related stressors (both mean-centered). Pre, prepandemic baseline.

For each risk or protective factor, we fit 2 longitudinal path models. The first model examined the main effect of each factor on internalizing problems at wave 1 and wave 2 (Figure 2A). The second model examined the interaction between each risk and protective factor with exposure to pandemic-related stressors. For each model, both the risk or protective factor and the pandemic-related stressors reported at wave 1 and wave 2 were mean centered, and an interaction term was computed by multiplying them (Figure 2B). Models all fit the data well. Model fit statistics and full model output for all models are presented in the Supplement.

Because 14 total path models were examined, false discovery rate (FDR) correction (52) was used to correct for multiple comparisons within each domain of analysis (emotion regulation: 6 models; amygdala activation: 4 models; amygdala and hippocampal volume: 4 models). Therefore, FDR-corrected *p* values are indicated in Results and Table 2 as FDR-*p.* Simple slopes analysis was used to follow up significant interactions. More detail as well as reproducible code and output (i.e., R Markdown; https://rmarkdown.rstudio.com/) of all analyses are provided in the Supplement, and all data and analysis codes are available at https://github.com/dgweissman/COVID.

**Table 2:** Results of Regression Analyses From Longitudinal Path Models

Independent Variables	Wave 1 Internalizing Problems					Wave 2 Internalizing Problems				
	*B*	SE	β	*p*	FDR*-p*	*B*	SE	β	*p*	FDR*-p*
Emotion Regulation										
Rumination	0.007	0.003	0.209	.018	.036	0.001	0.003	0.021	.791	.946
Expressive suppression	0.078	0.022	0.265	.000	.000	0.065	0.027	0.172	.018	.108
Cognitive reappraisal	−0.120	0.023	−0.374	.000	.000	−0.015	0.033	−0.037	.647	.946
Rumination × stress	0.000	0.001	−0.013	.842	.842	−0.001	0.002	−0.046	.537	.946
Expressive suppression × stress	0.023	0.011	0.136	.044	.066	0.001	0.014	0.005	.946	.946
Cognitive reappraisal × stress	−0.011	0.011	−0.067	.335	.402	−0.003	0.012	−0.017	.822	.946
Amygdala Activation										
Left amygdala	−0.054	0.019	−0.233	.005	.012	−0.005	0.023	−0.017	.830	.939
Right amygdala	−0.008	0.021	−0.034	.694	.802	−0.006	0.024	−0.019	.811	.939
Left amygdala reactivity × stress	−0.028	0.010	−0.200	.006	.012	−0.001	0.013	−0.006	.939	.939
Right amygdala reactivity × stress	−0.003	0.010	−0.022	.789	.802	0.014	0.013	0.083	.280	.939
Brain Structure										
Amygdala volume	−0.047	0.067	−0.080	.478	.637	0.060	0.077	0.079	.439	.785
Hippocampal volume	−0.027	0.029	−0.099	.364	.637	0.019	0.035	0.055	.585	.785
Amygdala volume × stress	−0.044	0.025	−1.156	.083	.332	−0.005	0.037	−0.103	.888	.888
Hippocampal volume × stress	−0.004	0.014	−0.021	.784	.784	−0.009	0.016	−0.043	.589	.785

Each row represents a separate path model. Covariates for all models were age, sex, and internalizing problems at the previous wave. Full results of each model and reproducible analysis code can be found in the Supplement.

*B*, unstandardized coefficient; β, standardized coefficient; *FDR-p*, false discovery rate–corrected *p* values using the Benjamini and Hochberg procedure; *p*, uncorrected *p* values.

## Results

### Descriptive Statistics

Descriptive statistics and bivariate associations between all variables are provided in Table 1. See Figure S2 for the distribution of exposure to pandemic-related stressors.

**Table 1:** Descriptive Statistics and Bivariate Correlations

Variable	*n*	Mean	SD
Baseline			
1 Sex, Female	145	0.43	0.50
2 Income-to-Needs Ratio	143	3.75	1.79
3 Youth Self-Report Internalizing Problems	145	51.70	10.80
4 Rumination	145	7.88	6.21
5 Intracranial Volume, mm^3^ × 10^6^	122	1.55	0.14
6 Amygdala Volume, mm^3^ × 10^3^	122	3.08	0.36
7 Hippocampal Volume, mm^3^ × 10^3^	122	8.35	0.78
8 Left Amygdala Activation	122	0.24	0.90
9 Right Amygdala Activation	122	0.16	0.87

Pandemic Follow-up 1			
10 Age	143	14.40	0.46
11 Pandemic-Related Stressors	143	2.28	1.74
12 Strength and Difficulties Questionnaire Internalizing Problems	143	3.79	3.32
13 Cognitive Reappraisal	143	3.26	0.65
14 Expressive Suppression	143	2.84	0.71

Pandemic Follow-up 2			
15 Age	124	14.90	0.48
16 Pandemic-Related Stressors	124	2.16	1.71
17 Strength and Difficulties Questionnaire Internalizing Problems	124	5.05	3.75

	Correlations																
	1	2	3	4	5	6	7	8	9	10	11	12	13	14	15	16	17
1	–																
2	0.21^a^	–															
3	−0.38^a^	−0.26∗	–														
4	−0.16	0.01	0.50^a^	–													
5	−0.52^a^	0.11	0.09	−0.00	–												
6	−0.50^a^	0.17	0.04	−0.00	0.66^a^	–											
7	−0.46^a^	0.22^a^	0.10	−0.01	0.64^a^	0.69^a^	–										
8	−0.11	0.00	0.09	−0.02	−0.01	−0.02	−0.09	–									
9	0.01	−0.02	0.07	−0.04	−0.07	−0.05	−0.08	0.57^a^	–								
10	0.05	0.42^a^	−0.12	0.02	0.02	0.01	−0.04	0.06	−0.05	–							
11	0.04	−0.13	0.00	0.03	−0.04	−0.12	−0.02	−0.26^a^	−0.26^a^	−.05	–						
12	0.28^a^	−0.14	0.10	0.21^a^	−0.31^a^	−0.29^a^	−0.30^a^	−0.25^a^	−0.03	0.00	0.46^a^	–					
13	0.00	0.10	−0.11	0.00	0.00	0.00	0.01	0.12	−0.08	−0.04	−0.26^a^	−0.39^a^	–				
14	0.08	−0.08	0.05	0.03	−0.12	−0.13	−0.14	−0.04	−0.02	0.10	0.10	0.30^a^	−0.16	–			
15	0.03	0.40^a^	−0.18	0.02	0.03	0.01	0.02	−0.05	−0.12	1.00^a^	0.07	0.11	−0.08	0.10	–		
16	0.07	−0.25^a^	0.09	0.02	−0.20^a^	−0.40^a^	−0.39^a^	−0.14	−0.15	−0.12	0.42^a^	0.30^a^	−0.09	0.15	−0.08	–	
17	0.26^a^	0.02	0.07	0.13	−0.30^a^	−0.26^a^	−0.24^a^	−0.14	0.03	−0.04	0.26^a^	0.59^a^	−0.23^a^	0.32^a^	−0.03	0.37^a^	–

a *p* < .05.

### Pandemic-Related Stressors and Internalizing Problems

**Figure 3:**
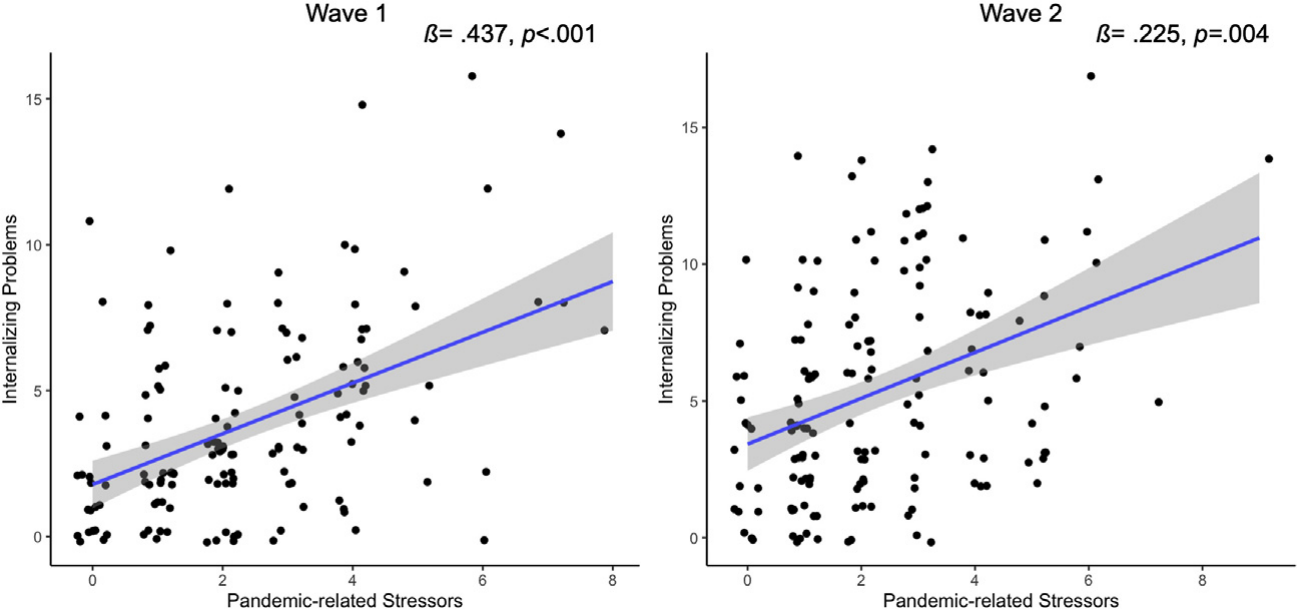
Association between pandemic-related stressors and internalizing problems. Exposure to pandemic-related stressors is positively associated with internalizing problems at both wave 1 (April to May 2020) and wave 2 (November 2020 to January 2021).

Internalizing problems increased significantly during the pandemic from wave 1 to wave 2 (mean difference 1.18, 95% CI 0.59–1.77, *t*_116_ = 5.80). Adolescents who experienced more pandemic-related stressors early in the pandemic reported higher internalizing problems at the pandemic wave 1 assessment (unstandardized *B* = 0.834, SE = 0.134, standardized β = 0.437, *t*_138_ = 6.19, *p* < .001), controlling for age, sex, and internalizing problems at the prepandemic baseline. Notably, while the zero-order correlations between prepandemic internalizing problems and wave 1 internalizing problems were small and nonsignificant (*r* = 0*.*10, *p* = .215), their association in the regression model that included pandemic-related stressors was modest and significant (*B* = 0.209, SE = 0.070, β = 0.225, *t*_138_ = 2.96, *p* = 0.004). The pandemic-related stressors occurring between the wave 1 and wave 2 pandemic assessments were associated with higher internalizing problems at wave 2 (*B* = 0.486, SE = 0.166, β = 0.225, *t*_112_ = 2.93, *p* = .004), controlling for age, sex, and wave 1 internalizing problems (Figure 3).

### Emotion Regulation, Pandemic-Related Stressors, and Internalizing Problems

Youths who reported more frequent use of rumination at baseline had higher internalizing problems at the wave 1 pandemic assessment (*B* = 0.007, SE = 0.003, β = 0.209, FDR-*p* = .036), controlling for age, sex, and baseline symptoms, but not at the wave 2 pandemic assessment (*B* = 0.001, SE = 0.003, β = 0.021, FDR-*p* = .946), controlling for age, sex, and wave 1 symptoms.

Greater use of expressive suppression reported at wave 1 during the pandemic was associated with higher internalizing problems at wave 1 (*B* = 0.078, SE = 0.022, β = 0.265, FDR-*p* < .001), controlling for age, sex, and prepandemic symptoms. Greater use of expressive suppression reported at wave 1 during the pandemic was also associated with higher internalizing problems at wave 2, controlling for age, sex, and wave 1 symptoms, but this association was not significant after multiple comparisons correction (*B* = 0.065, SE = 0.027, β = 0.172, FDR-*p* = .946).

Greater use of cognitive reappraisal reported early in the pandemic was associated with higher internalizing problems at wave 1 (*B* = −0.120, SE = 0.023, β = −0.374, FDR-*p* < .001), controlling for age, sex, and prepandemic symptoms. However, greater use of cognitive reappraisal reported at wave 1 during the pandemic was not associated with internalizing problems at wave 2 (*B* = −0.015, SE = 0.033, β = −0.037, FDR-*p* = .946), controlling for age, sex, and wave 1 symptoms. Use of rumination, cognitive reappraisal, and expressive suppression did not interact with pandemic-related stressors to predict internalizing problems at either point in the pandemic (Table 2).

### Amygdala Activation, Pandemic-Related Stressors, and Internalizing Problems

**Figure 4:**
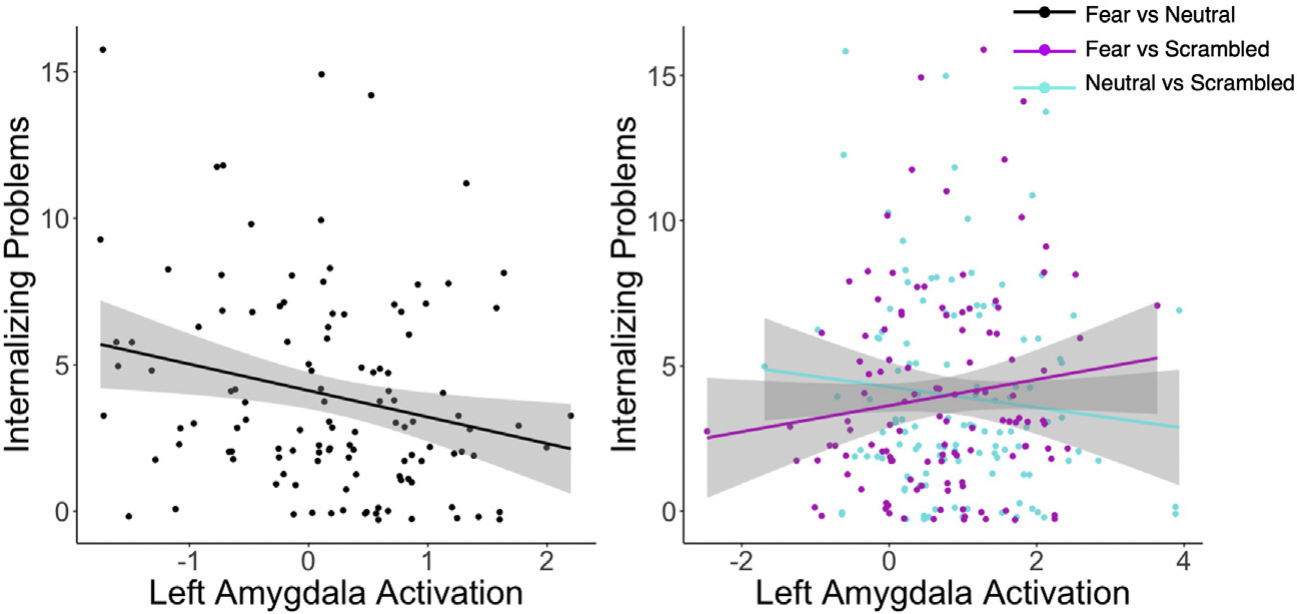
Left amygdala discrimination between fearful and neutral faces and internalizing problems. Left amygdala reactivity to fearful vs. neutral faces was negatively associated with internalizing problems early in the pandemic. Examining the association of both fearful faces and neutral faces relative to scrambled faces revealed that this association was attributable to both greater activation to neutral faces and lower activation to fearful faces, but neither of those contrasts was significantly associated with internalizing problems.

Although highly heterogeneous, amygdala activation to fearful faces was significantly greater than amygdala activation to neutral faces on average (mean [SD] 0.239 [0.897], *p <* .001). Lower left amygdala activation to fearful relative to neutral faces before the pandemic was associated with greater internalizing problems in the early phase of the pandemic (*B* = −0.054, SE = 0.019, β = −0.233, FDR-*p* = .012), controlling for age, sex, and prepandemic symptoms. To determine whether this association was driven by greater reactivity to neutral faces or less reactivity fearful faces, we decomposed this contrast by examining the association of left amygdala activation to fearful versus scrambled faces and to neutral versus scrambled faces with internalizing problems, neither of which was significant (Figure 4). No associations were observed for right amygdala activation (Table 2).

**Figure 5:**
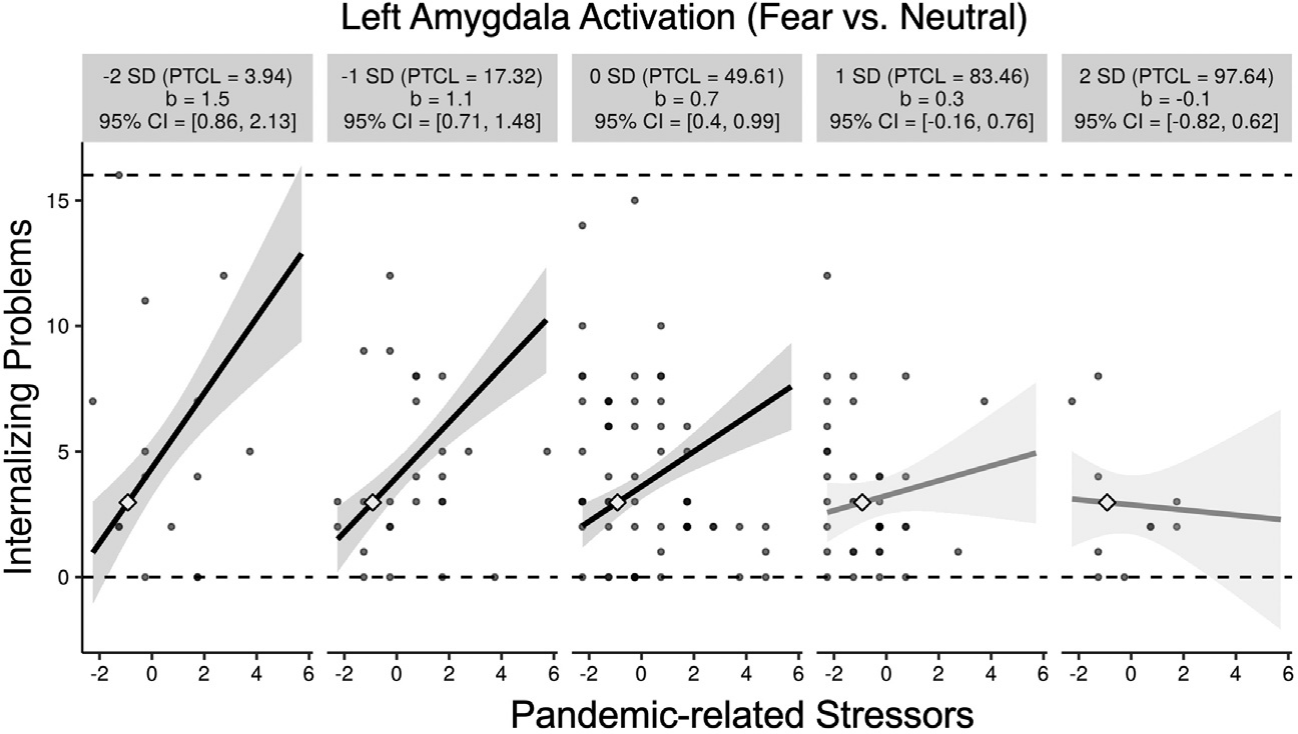
Amygdala discrimination, pandemic-related stressors, and internalizing symptoms. Pandemic-related stressors are more strongly associated with internalizing problems in participants with lower amygdala reactivity to fearful vs. neutral faces (i.e., greater differentiation of threatening vs. neutral stimuli). Among participants with high amygdala response to fearful vs. neutral faces, the association between pandemic-related stressors and internalizing problems is not significant. PTCL, percentile.

Left amygdala activation to fearful relative to neutral faces moderated the association of pandemic-related stressors with internalizing problems early in the pandemic (*B* = −0.028, SE = 0.010, β = −0.200, FDR-*p* = .012), controlling for age, sex, and prepandemic symptoms. Pandemic-related stressors were positively associated with internalizing problems among youths with low to mean amygdala reactivity, but not among youths with high amygdala reactivity to fearful versus neutral faces (Figure 5). No significant interactions were observed for right amygdala activation (Table 2).

### Brain Structure, Pandemic-Related Stressors, and Internalizing Problems

Neither amygdala volume nor hippocampal volume at the baseline visit was associated with internalizing problems at either point during the pandemic, and neither interacted with pandemic-related stressors in predicting internalizing problems at either wave (Table 2).

## Discussion

The COVID-19 pandemic has produced sudden and unprecedented changes in the lives of adolescents that have introduced many novel stressors and exacerbated risk for anxiety and depression (7, 8, 9, 10, 11), which were already on the rise among adolescents in the United States (51). The current study examined factors contributing to individual differences in the impact of the pandemic on adolescent internalizing problems in the early phase of the pandemic (late spring 2020) and again 6 months later (late fall/winter 2020). Adolescents demonstrated worsening internalizing problems over the course of the pandemic, and adolescents with high exposure to pandemic-related stressors had particularly elevated internalizing problems relative to the previous time point. We found significant variability in internalizing problems based on the use of different emotion regulation strategies before and during the pandemic, including rumination, cognitive reappraisal, and expressive suppression, as well as amygdala activation measured before the pandemic. Specifically, greater left amygdala activation to neutral relative to fearful faces was associated with higher internalizing problems during the pandemic. These findings support widespread concerns about the mental health consequences of the pandemic in adolescents, particularly for adolescents exposed to more pandemic-related stressors. Use of maladaptive emotion regulation strategies and less differentiated neural responses to threatening and neutral cues may make youths particularly vulnerable to the increased exposure to stressors that have characterized the pandemic.

The degree of exposure to pandemic-related stressors was associated with worsening internalizing problems over time both early in the pandemic and as the pandemic progressed. This is consistent with cumulative risk models, which argue that stressors have a cumulative impact on mental health (45), and prior work on exposure to stressors and psychopathology during community-wide disruptions, such as natural disasters and terrorist attacks (53, 54, 55). These findings therefore characterize the impact of a unique and unprecedented source of stress, but conform to the general patterns of association observed in other stressful contexts. Further, although the stressors examined here (e.g., social isolation, parental job loss, food insecurity) were exacerbated by the pandemic and lockdowns, these types of stressors can and do occur in other contexts. Conversely, social isolation owing to the lockdowns may have further exacerbated the mental health impacts of these stressors by limiting the social support available to adolescents. While the pandemic continues, policies aimed at mitigating the stressors experienced by families (e.g., unemployment benefits, return to in-person schooling) may have the greatest potential to decrease the impact of the pandemic on adolescents’ mental health.

Internalizing problems before the pandemic were not correlated with internalizing problems during the pandemic. However, they were significantly associated in the regression model that included pandemic-related stressors, with an effect size consistent with the stability that might be expected between two moderately correlated measures (56) collected 2 to 3 years apart during adolescence, suggesting that the strong association between pandemic-related stressors and internalizing problems may have reduced the stability of internalizing problems in early adolescence.

Greater use of maladaptive emotion regulation strategies, including rumination and expressive suppression, and lower use of cognitive reappraisal predicted worse internalizing problems early in the pandemic. Overall, greater use of adaptive emotion regulation strategies (i.e., cognitive reappraisal) and lower use of maladaptive strategies (i.e., rumination and suppression) were associated with lower internalizing problems during the pandemic but were not as effective at reducing the impact of pandemic-related stressors. Further, perhaps owing to the high rank-order stability of internalizing problems during the pandemic, the protective influence of emotion regulation strategy use was specific to the early months of the pandemic. Nonetheless, these results provide some evidence that interventions focused on teaching adolescents adaptive emotion regulation skills may be an effective means of reducing internalizing psychopathology during the pandemic and beyond.

Higher left amygdala activation to neutral compared with fearful faces before the pandemic had a modest and significant association with internalizing problems in the early phase of the pandemic and moderated the association of pandemic-related stressors with internalizing problems, such that the association was stronger among youths with higher left amygdala activation to neutral relative to fearful faces. The direction of these associations is unexpected. In prior work, the association between stressful life events and depression was stronger in youths with greater amygdala responses to fearful and angry faces compared with shapes (15). Decomposing our finding by examining fearful and neutral faces each compared with scrambled faces was inconclusive. However, our results may nonetheless reflect that youths who differentiate less between fearful faces, which indicate the presence of a potential threat in the environment, and neutral faces, which are ambiguous and do not clearly reflect the presence of a threat, may interpret neutral or ambiguous cues as more threatening. Amygdala reactivity to potential threats is amplified in contexts of high uncertainty (57,58). Youths who experience violence and other forms of childhood trauma—experiences characterized by high threat and uncertainty that confer greater risk for internalizing problems—are more likely to perceive neutral faces as angry (59,60) and to attribute hostile intent to ambiguous social cues more generally (61). Less differentiated amygdala reactivity to threatening compared with neutral cues, perhaps reflecting greater uncertainty in the interpretation of the more ambiguous neutral faces, may make adolescents more vulnerable to developing internalizing problems when faced with chronic stress and uncertainty, common experiences during the COVID-19 pandemic. Indeed, amygdala activation was not correlated with internalizing problems assessed concurrently at the prepandemic baseline, which suggests that less differentiated amygdala reactivity to threatening compared with neutral cues may be more an indicator of vulnerability to future stress and uncertainty than a direct correlate of internalizing problems.

Neither amygdala nor hippocampal volume was associated with internalizing problems directly, and amygdala and hippocampal volume did not interact with pandemic-related stressors in predicting internalizing problems at either time point in the pandemic. Thus, we failed to replicate the pattern of smaller hippocampal and amygdala volume being associated with greater vulnerability to stress that has been observed previously among youths exposed to childhood violence (16,41). It is possible that differences in hippocampal and amygdala volume resulting from exposure to traumatic stressors may reflect specific structural alterations that impact responses to stress more so than the variability in hippocampal and amygdala volume observed in samples without high trauma exposure.

This study confirms and replicates the concerning impact that the COVID-19 pandemic has had on adolescent mental health (7, 8, 9, 10, 11) and reveals several sources of meaningful variability in symptoms of depression and anxiety among adolescents. Exposure to stressors is by far the most robust and consistent predictor of internalizing problems examined in this study, consistent with substantial evidence on the link between stressors and internalizing psychopathology (62,63). Adolescent-reported use of maladaptive emotion regulation strategies was also a modest predictor that can be measured inexpensively and easily and could help to identify youths who might be particularly at risk for developing internalizing problems and thus might benefit from mental health interventions. Alternatively, while the modest associations of lower amygdala discrimination between fearful and neutral faces in increasing vulnerability to pandemic-related stressors informs our understanding of the mechanisms underlying stress sensitivity in adolescents, this measure is substantially more costly and difficult to acquire and may offer marginal additional clinical or practical utility in identifying the most vulnerable youths relative to self-reports and parent reports of stressors and emotion regulation strategy use.

While this study has several strengths, including rich, multi-informant (parent and adolescent) and multimodal (self-reports, neuroimaging) data on a relatively large sample of adolescents assessed at 3 time points before and during the COVID-19 pandemic, it also has several limitations that should be considered when interpreting our findings. First, because the participants in this sample all were within a relatively narrow 2-year age band, we were unable to characterize the specificity or generalizability of these findings across other developmental windows. Second, the stimuli in the fMRI task of the current study included only fearful and neutral faces. Therefore, we were unable to determine whether the association between reduced differentiation in the amygdala between fearful and neutral faces and internalizing problems in the context of pandemic-related stressors is specific to fear or may reflect responses to negative emotional cues more generally. Third, because our neuroimaging and emotion regulation measures were collected at only one time point, we were unable to examine their stability or the consistency of their associations with internalizing problems across multiple time points as we were with pandemic-related stressors. Finally, while the pandemic-related stress measure demonstrated convergent validity via consistently moderate associations with internalizing problems at both waves, it nonetheless had the limitation of equally weighting stressors that may have had highly variable objective and subjective impacts. Despite these limitations, when synthesized with the many other developmental studies underway during the pandemic, we anticipate these longitudinal, multi-informant, multimodal findings contributing to a richer and more complete understanding of factors contributing to adolescent mental health during the pandemic as well as factors contributing to stress vulnerability more broadly.

Internalizing problems have increased steadily among adolescents during the COVID-19 pandemic, particularly for individuals who have experienced a high number of pandemic-related stressors as well as among adolescents who habitually use maladaptive emotion regulation strategies such as rumination and suppression and use cognitive reappraisal less frequently. Lower differentiation of amygdala activation between threatening and neutral stimuli, assessed before the pandemic, is also associated with increased vulnerability to stress-related internalizing problems. Attempts to identify adolescents most in need of mental health interventions would benefit from screening for exposure to pandemic-related stressful experiences and use of emotion regulation strategies. Such efforts may help to identify adolescents in need of extra support and intervention to mitigate the mental health crisis posed by the COVID-19 pandemic.

## Supplementary Material

Supplementary Material

Key Resources Table
