# Supplementary material for: Contributions of Emotion Regulation and Brain Structure and Function to Adolescent Internalizing Problems and Stress Vulnerability During the COVID-19 Pandemic: A Longitudinal Study
Source: Biol Psychiatry Glob Open Sci. 2021 Jun 12;1(4):272–82. doi: 10.1016/j.bpsgos.2021.06.001 (PMC8643098; doi:10.1016/j.bpsgos.2021.06.001)
Supplement: Supplementary Material [file mmc1.pdf]

Contributions of Emotion Regulation and Brain Structure and Function to Adolescent Internalizing Problems and Stress Vulnerability During the COVID-19 Pandemic: A Longitudinal Study

Supplemental Information

1.	Supplemental Methods	2
2.	Supplemental References	8
3.	Table S1	10
4.	Table S2	11
5.	Table S3	12
6.	Figure S1	13
7.	Figure S2	14
8.	Sensitivity Analyses	15
9.	Reproducible analyses and results (Rmarkdown)	17

Measures

Rumination. The Children's Response Styles Questionnaire (CRSQ) (1) is a 25-item scale that assesses the extent to which children respond to sad feelings with rumination, defined as self-focused thought concerning the causes and consequences of depressed mood, distraction, or problem-solving. For each item, youth are asked to rate how often they respond in that way when they feel sad on a 4-point Likert scale ranging from almost never (1) to almost always (4). The rumination subscale includes 13 items that are summed, with higher scores indicating greater habitual engagement of rumination. Sample items include: "*Think about a recent situation wishing it had gone better*" and "*Think why can't I handle things better?*" The CRSQ has demonstrated good psychometric properties (1) and had good internal consistency in this sample (α = .86).

Pandemic-related Stressors. We developed a set of questions to assess pandemic-related stressors that was administered to children and caregivers. The assessment included *health, financial, social, school, and physical environment stressors* that occurred within the month prior to the first assessment during COVID-19, and that occurred in the intervening period between the first and second COVID-19 assessments at the final follow-up. Seven of the stressors were related to the health of participants or close others (e.g. contracting COVID-19); four were related to financial impacts of COVID-19 (e.g. parent lost a job); four were related to disruptions to social life (e.g. social isolation); and three related to noise and crowding (e.g. the environment where the youth does school work is noisy). Stressors were coded as present if they were endorsed by either the youth or their parent and summed, using a cumulative risk approach (2). See Table S1. The complete scale can be found at https://osf.io/drqku/.

Cognitive Reappraisal and Expressive Suppression. The Emotion Regulation Questionnaire (ERQ) (3) is a 10-item self-report questionnaire assessing the tendency to use cognitive reappraisal and expressive suppression. Six items pertain to cognitive reappraisal (e.g. "I control my emotions by changing the way I think about the situation I'm in"), and 4 pertain to expressive suppression (e.g. "I control my emotions by not expressing them"). Responses are provided on a 7-point Likert scale ranging from strongly disagree (1) to strongly agree (7). Separate sum scores are calculated for cognitive reappraisal and expressive suppression. Higher scores indicate a greater tendency to use that strategy. Both the reappraisal ($\alpha = 0.79$) and expressive suppression sub-scales ($\alpha = 0.75$) demonstrated adequate internal consistency in this sample.

Internalizing problems. Internalizing problems at the pre-pandemic baseline were assessed based on child report on the Youth Self-Report (YSR) (4), among the most widely-used measures of youth emotional and behavioral problems. The YSR had good internal consistency in this sample ($\alpha = .86$). Nineteen participants reported clinical levels of internalizing problems above the 90th percentile (T-Score > 63). At the Wave 1 and Wave 2 COVID-19 follow-up assessments adolescents completed the Strengths and Difficulties Questionnaire (SDQ; Goodman, 1997), a 25-item scale comprised of five subscales, with five items each (5). The Internalizing Problems composite from the SDQ had adequate internal consistency in this sample at Wave 1 ($\alpha = 0.74$) and Wave 2 ($\alpha = 0.78$). To make the scales comparable, both YSR and SDQ internalizing scores were scaled to values between 0 and 1, i.e. proportion of maximum scaling (6).

Emotional Processing fMRI Task

The emotional processing task consisted of 2 runs of 9 18-second blocks, during which participants passively viewed emotional face stimuli. Faces were drawn from the NimStim stimulus set (7). Each run consisted of 3 blocks of neutral, fearful, and scrambled faces and 3 ITI blocks displayed in a pseudo-random order that ensured that no block type was displayed twice in a row. The "calm" faces from this dataset were used as neutral expressions, as these expressions are potentially less emotionally evocative than neutral faces (7), which can be perceived as negatively-valenced (7). During each block, 36 faces of different actors expressing the same emotion were displayed for 300 ms each, with 200 ms between each face, based on prior face processing tasks (8). At one point during each block participants were prompted to indicate by an index or middle finger button press whether the last face they saw was male or female (or whether a dot appeared on the left or right side of the screen for scrambled face blocks) to ensure they were paying attention to the stimuli.

MRI Data Acquisition and Preprocessing

Before undergoing scanning, youth were trained to minimize head movements in a mock scanner. They watched a movie with a head-mounted motion tracker that stopped playing if a movement of over 2 mm occurred. In addition, in the scanner we used an inflatable head-stabilizing pillow to restrict movement.

Scanning was performed on a 3T Phillips Achieva scanner at the University of Washington Integrated Brain Imaging Center using a 32-channel head coil. T1-weighted MPRAGE volumes were acquired (repetition time = 2530 ms, TE=3.5ms, flip angle=7°, FOV=256×256, 176 slices, in-plane voxel size=1mm^3) for co-registration with fMRI data. Blood oxygenation level dependent (BOLD) signal during functional runs was acquired using a

gradient-echo T2*-weighted echo planar imaging (EPI) sequence. Thirty-seven 3-mm thick slices were acquired sequentially and parallel to the AC-PC line (TR=2s, TE=25ms, flip angle=79°, Inter-slice gap=.6mm, FOV=224×224×132.6, matrix size=76x74). Prior to each scan, four images were acquired and discarded to allow longitudinal magnetization to reach equilibrium.

Preprocessing and statistical analysis of fMRI data was performed for the purposes of extracting activation to fearful vs. neutral faces from left and right amygdala ROIs, in a pipeline using Gnu Make, a software development tool designed for building executables from source files that can be used to create neuroimaging workflows that rely on multiple software packages (9). The following preprocessing steps were applied: 1) motion correction followed by slice-time correction in FSL; 2) skull-stripping using FSL's bet tool; 3) despiking using AFNI's 3dDespike tool; and 4) smoothing with a 6mm full-width half-max kernel using SUSAN in FSL. Outlier volumes in which framewise displacement exceeded 1mm, the derivative of variance in BOLD signal across the brain (DVARS) exceeded the upper fence (above 75th percentile + 1.5 × inter-quartile range), or signal intensity was more than 3 SD from the mean were regressed out of person-level models. Six rigid-body motion regressors and the time-series extracted from white matter and ventricles were included in person-level models to reduce noise associated with motion and physiological fluctuations. Person- and group-level models were estimated in FSL using a boxcar function convolved with the hemodynamic response function (HRF). Separate models were estimated for each of the two runs, and then combined into a group-level model. Following estimation of person-level models, the resulting contrast images were normalized into standard space, and anatomical co-registration of the functional data with each participant's T1-weighted image was performed using Advanced Normalization Tools (ANTs) software. Six

participants were excluded for poor quality of structural images that compromised coregistration with functional data. Four participants had runs where greater than 20% of the volumes were censored. For those participants only one run of the task was used. 122 participants had usable fMRI data and were included in analyses involving amygdala responses.

Left and right amygdala ROIs were extracted from each participant based on the Harvard Oxford subcortical probabilistic structural atlas, thresholded at 20% probability and warped back into each subjects' native space. Mean z-scores were extracted from the right and left amygdala for the contrasts fear vs. neutral (for primary analyses), as well as fear vs. scrambled and neutral vs. scrambled (for post-hoc analysis to determine whether findings were driven by differences in response to the fearful or neutral faces) for each participant.

Structural MRI processing

Measures of left and right hippocampal and amygdala volume and total intracranial volume were obtained using automatic segmentation in FreeSurfer 5.3. Six participants were excluded for poor quality of structural images that compromised automatic segmentation. Each segmentation was inspected manually by at least two investigators to ensure that no segmentation errors were present. No manual edits were performed on subcortical segmentations. Following prior work (10), right and left volumes were summed to create bilateral hippocampal and amygdala volume measures. To keep variables on a similar scale for regression analyses, subcortical volumes were divided by 1,000, and intracranial volume was divided by 1,000,000. 122 participants had usable structural MRI data and were included in analyses involving hippocampal and amygdala volume.

Path Analyses

For each risk or protective factor, we fit 2 longitudinal path models. The first model examined the main effect of each factor on internalizing problems at Wave 1 and Wave 2. Stability paths were included from internalizing problems at pre-pandemic baseline to internalizing problems at Wave 1 and internalizing problems at Wave 1 to Wave 2. Paths were also included from sex to internalizing problems at Wave 1 and Wave 2 and from age at Wave 1 and Wave 2 to internalizing problems at the same age. A stability path was included from age at Wave 1 to Wave 2. Covariances were also included between internalizing problems at pre-pandemic baseline, sex, age at Wave 1, and the risk or protective factor in each model. Residual covariances were included between internalizing problems at Wave 1 and age at Wave 2.

The second model examined the interaction between each risk and protective factor with pandemic-related stress. For each model, both the risk or protective factor and pandemic-related stress reported at Wave 1 and Wave 2 were mean-centered, and an interaction term was computed by multiplying them. In addition to the paths specified in the main effects model, paths were included from pandemic-related stress reported at each Wave to internalizing problems reported at the same Wave and from the interaction term and internalizing problems. Stability paths were included between both the pandemic-related stress variables and the interaction terms. Covariances were included between pandemic-related stress at and the interaction term at Wave 1 and the other exogenous predictors. Residual covariances were included between pandemic-related stress and the interaction term at Wave 2, age at Wave 2, and internalizing problems at Wave 1. For models examining associations with hippocampal and amygdala volume, paths were also included from total intracranial volume to internalizing problems at the Wave 1 and Wave 2 follow-ups, and covariances were included between total intracranial

volume and the other predictors in the model. Reproducible code and output (Rmarkdown) of all analyses are provided on pages 13-76, and all data and analysis code are available at https://github.com/dgweissman/COVID.

Model fits were evaluated using a chi-square test, comparative fit index (CFI), root mean squared error of approximation (RMSEA), and Standardized Root Mean Square Residual (SRMR). All models fit the data well. With one exception, chi-square tests of model fit were all nonsignificant (p-values ranged from .044-.532). CFI ranged from .988-1.000; RMSEA ranged from .000 to .072; SRMR ranged from .005 to .052.

Supplemental References

1. Abela JRZ, Brozina K, Haigh EP (2002): An examination of the response styles theory of depression in third- and seventh-grade children: A short-term longitudinal study. *J Abnorm Child Psychol*. https://doi.org/10.1023/A:1019873015594
2. Evans GW, Li D, Whipple SS (2013): Cumulative risk and child development. *Psychol Bull* 139: 1342–1396.
3. Gross JJ, John OP (2003): Individual Differences in Two Emotion Regulation Processes: Implications for Affect, Relationships, and Well-Being. *J Pers Soc Psychol*. https://doi.org/10.1037/0022-3514.85.2.348
4. Achenbach TM (1991): Manual for the child behavior checklist/4-18 and 1991 profile. *Burlington VT*.
5. Goodman A, Lamping DL, Ploubidis GB (2010): When to Use Broader Internalising and Externalising Subscales Instead of the Hypothesised Five Subscales on the Strengths and

Difficulties Questionnaire (SDQ): Data from British Parents, Teachers and Children. *J Abnorm Child Psychol* 38: 1179–1191.

6. Little TD (2013): *Longitudinal Structural Equation Modeling*. Guilford Press.
7. Tottenham N, Tanaka JW, Leon AC, McCarry T, Nurse M, Hare TA, *et al.* (2009): The NimStim set of facial expressions: Judgments from untrained research participants. *Psychiatry Res* 168: 242–249.
8. Somerville LH, Kim H, Johnstone T, Alexander AL, Whalen PJ (2004): Human amygdala responses during presentation of happy and neutral faces: Correlations with state anxiety. *Biol Psychiatry*. https://doi.org/10.1016/j.biopsych.2004.01.007
9. Askren MK, McAllister-Day TK, Koh N, Mestre Z, Dines JN, Korman BA, *et al.* (2016): Using Make for Reproducible and Parallel Neuroimaging Workflow and Quality-Assurance. *Front Neuroinformatics*. https://doi.org/10.3389/fninf.2016.00002
10. Weissman DG, Lambert HK, Rodman AM, Peverill M, Sheridan MA, McLaughlin KA (2020): Reduced hippocampal and amygdala volume as a mechanism underlying stress sensitization to depression following childhood trauma. *Depress Anxiety*. https://doi.org/10.1002/da.23062

Table S1: Participant Race/Ethnicity

	Wave 1		Wave 2	
**Race/Ethnicity**	**n**	**%**	**n**	**%**
Asian	13	9	12	10
Black	14	10	12	10
Latinx	18	12	14	11
White	96	66	78	63
Other	4	2	3	2

Table S2. COVID Experiences Survey Stressor Items

Item	Reporter	Subscale	T1 Frequency	T2 Frequency
Believe got sick with COVID-19 AND experienced symptoms OR Tested positive for COVID-19 OR was hospitalized	Child	Health	.070	.063
Parent or sibling was sick with COVID-19	Child	Health	.141	.297
Partner or close friend was sick with COVID-19	Child	Health	.047	.063
Other relative was sick with COVID-19	Parent	Health	.086	.078
Know someone who died of COVID-19	Child	Health	.180	.258
Parent is a healthcare worker and still working	Parent	Health	.117	.180
Parent is a grocery store or other frontline worker and still working	Parent	Health	.266	.219
Has felt lonely a few times a week or more	Child	Social	.203	.086
You or someone in your household has experienced racism, prejudice, or discrimination related to COVID-19	Child	Social	.016	.008
Has a difficult relationship with parent that has gotten worse since being home more	Child	Social	.078	.039
Has a difficult relationship with a sibling or someone else who lives in the home that has gotten worse since being home more	Child	Social	.047	.047
Has had difficulty getting schoolwork done at home	Child	Noise/ Crowding	.422	.328
The environment where you do work is noisy	Child	Noise/ Crowding	.180	.086
The house is crowded (people/room > 1 standard deviation above the mean; Evans & Kim, 2007)	Parent	Noise/ Crowding	.086	0.078
Either parent was laid off or went out of business	Parent	Financial	0.065	0.047
Experienced food insecurity	Parent	Financial	0.133	0.117
Been evicted or otherwise forced to leave home because of financial reasons	Parent	Financial	0	0
Experienced significant financial loss	Parent	Financial	0.203	0.258

Table S3: Correlations between Stress Subscales, Global Stress, and Internalizing Problems.

**Wave 1**	**1**	**2**	**3**	**4**	**5**	**6**
**1. Health Subscale**	-					
**2. Social Subscale**	.14	-				
**3. Financial Subscale**	-.05	.10	-			
**4. Noise/Crowding Subscale**	-.06	.25	.07	-		
**5. Total Stress Score**	.56	.59	.46	.55	-	
**6. Internalizing Problems**	.11	.48	.27	.31	.48	-

**Wave 2**	1	2	3	4	5	6
**1. Health Subscale**	-					
**2. Social Subscale**	.05	-				
**3. Financial Subscale**	.33	.10	-			
**4. Noise/Crowding Subscale**	-.07	.17	-.08	-		
**5. Total Stress Score**	.78	.40	.63	.32	-	
**6. Internalizing Problems**	.05	.38	.00	.38	.27	-

Figure S1. Distribution of Number of Pandemic-related Stressors Experienced at Waves 1 and 2

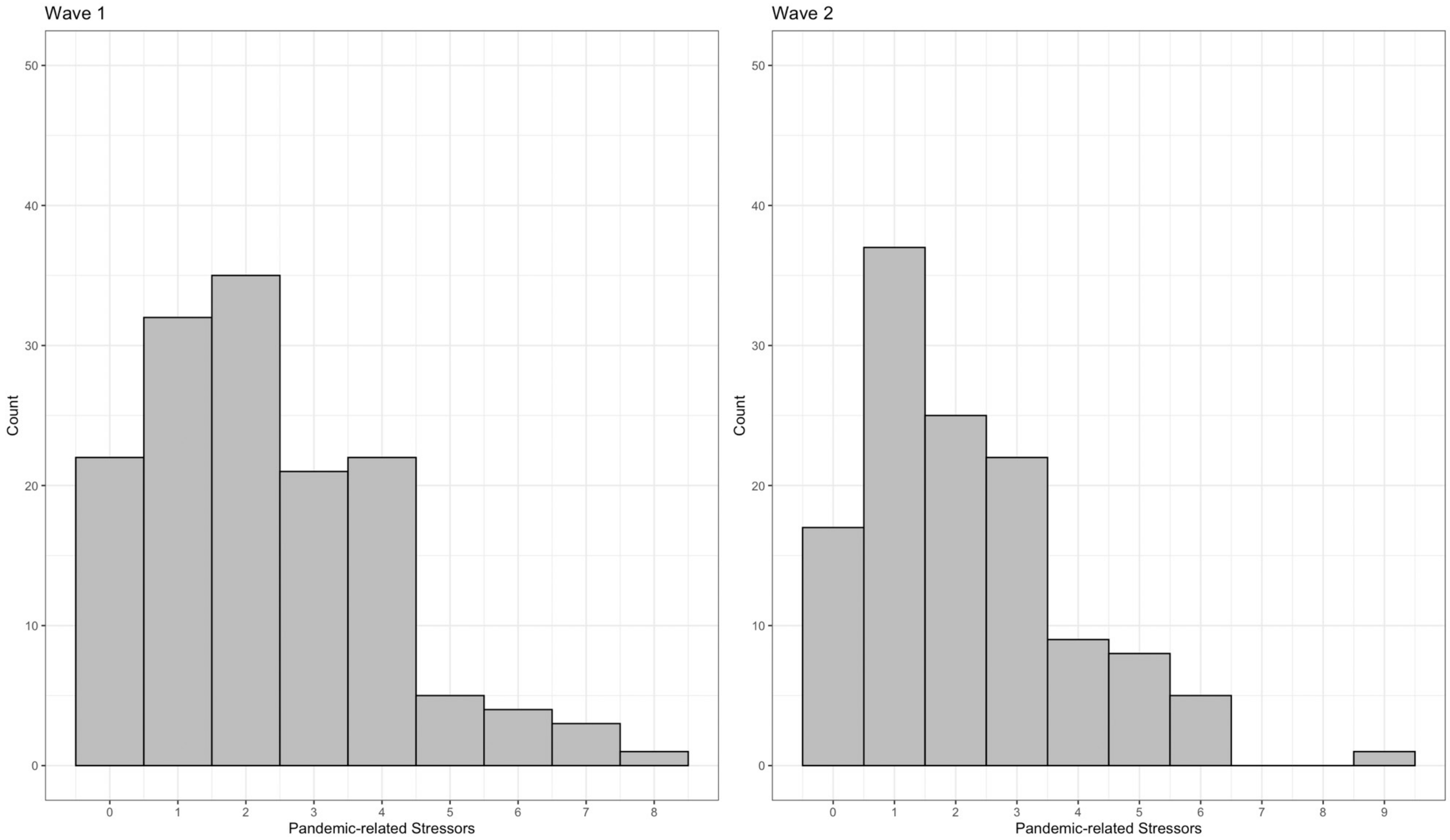

Figure S2: Expressive suppression, Pandemic-Related Stressors, and Internalizing Symptoms

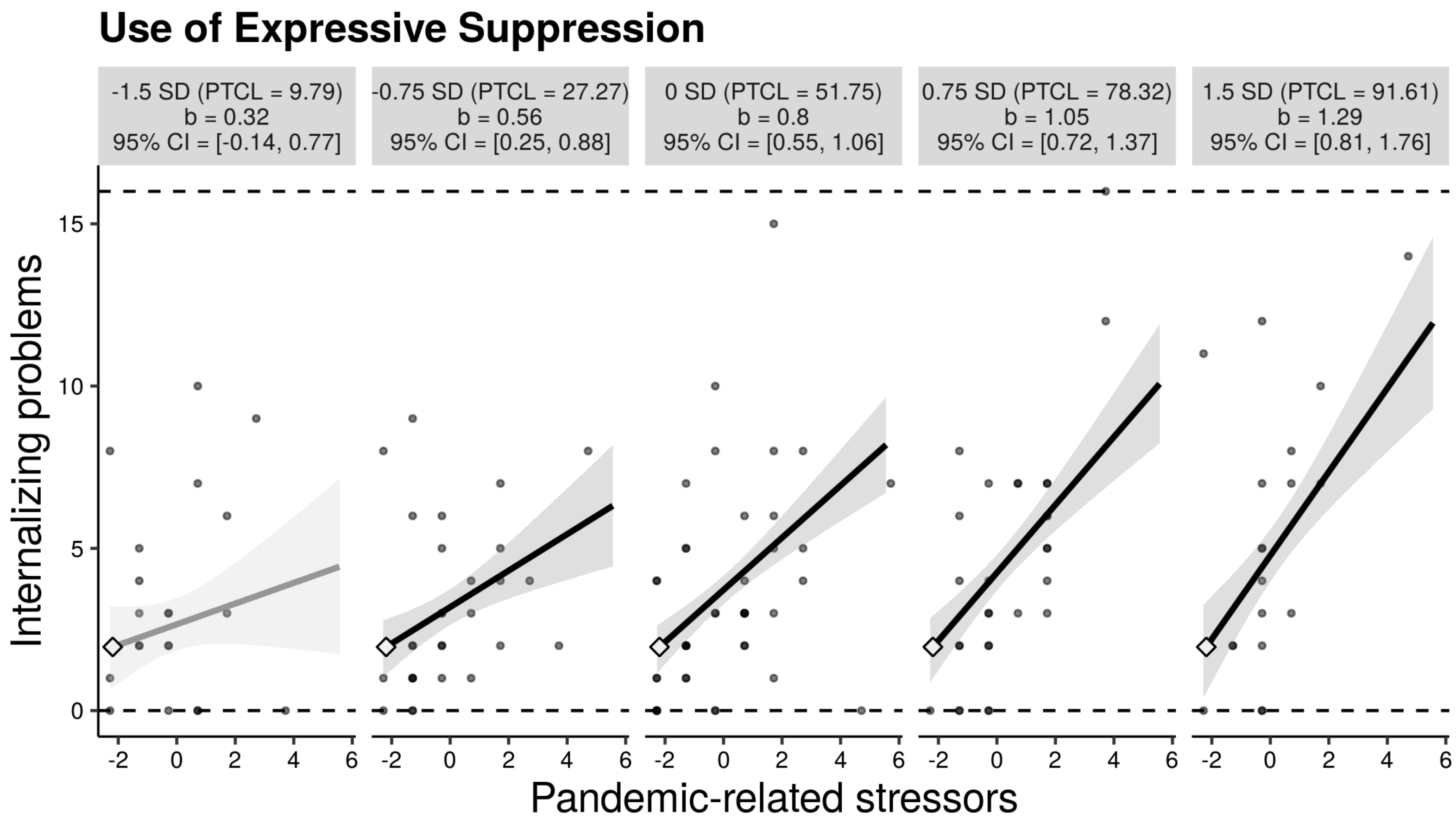

Figure 4: Pandemic-related stressors are more strongly positively associated with internalizing problems among participants who use expressive suppression more frequently. Figure produced using the interActive data visualization tool (11).

Sensitivity Analyses using 50% Probability Harvard Oxford Atlas Amygdala ROI

Due to reviewer concerns about the specificity of findings to the amygdala when using a 20% thresholded amygdala region of interest and a 6 mm smoothing kernel, we extracted amygdala ROIs with a more conservative 50% probability ROI. Linear regression was used to investigate the associations with left and right amygdala activation and internalizing problems at Wave 1 and the interactions between left and right amygdala activation and pandemic-related stress at Wave 1 in relation to internalizing problems at Wave 1. Results are summarized in the table below.

Table S4.
Association with internalizing problems at Wave 1

**Amygdala activation**	*B*	*SE*	*ß*	*p*
Left Amygdala	-.891	.323	-.244	.007
Right Amygdala	-.187	.343	-.045	.588
Left Amygdala Reactivity x Stress	-.376	.163	-.172	.024
Right Amygdala Reactivity x Stress	.016	.172	.008	.916

Covariates for all models were age, sex, and internalizing problems at pre-pandemic baseline.

Sensitivity Analyses controlling for mean framewise displacement and % of data excluded as motion outliers

We also conducted sensitivity analyses controlling for framewise displacement and the percentage of participant data that was censored due to motion outliers (>=1mm FD). Results are summarized below.

Table S5.
Association with internalizing problems at Wave 1

**Amygdala activation**	*B*	*SE*	*ß*	*p*
Left Amygdala	-.883	.329	-.227	.008
Right Amygdala	-.247	.349	-.061	.480
Left Amygdala Reactivity x Stress	-.452	.177	-.192	.012
Right Amygdala Reactivity x Stress	-.005	.174	-.002	.977

Covariates for all models were age, sex, internalizing problems at pre-pandemic baseline, mean framewise displacement, and percentage of data censored due to motion outliers.

Sensitivity Analyses excluding participants for whom only one run of fMRI data was used

Finally, sensitivity analyses were conducted excluding the 3 participants for whom only one run of data was used. Results are summarized below.

Table S6.
Association with internalizing problems at Wave 1

**Amygdala activation**	*B*	*SE*	*ß*	*p*
Left Amygdala	-.852	.328	-.218	.011
Right Amygdala	-.224	.349	-.055	.523
Left Amygdala Reactivity x Stress	-.437	.176	-.187	.015
Right Amygdala Reactivity x Stress	-.014	.174	-.007	.934

Covariates for all models were age, sex, and internalizing problems at pre-pandemic baseline.

Reproducible Analyses for: Contributions of emotion regulation and brain structure and function to adolescent internalizing problems and stress vulnerability during the COVID-19 pandemic: A longitudinal study

David Weissman

4/23/2021

Analyses

Change in internalizing from W1 to W2

```
t.test(dt.covid$SDQ_C_INT2, dt.covid$SDQ_C_INT1, paired = T)

##
##  Paired t-test
##
## data:  dt.covid$SDQ_C_INT2 and dt.covid$SDQ_C_INT1
## t = 5.8006, df = 116, p-value = 5.83e-08
## alternative hypothesis: true difference in means is not equal to 0
## 95 percent confidence interval:
##  0.07799623 0.15887801
## sample estimates:
## mean of the differences
##               0.1184371
```

Number of Pandemic-related Stressors (Figure S1)

```
splot1 <- ggplot(dt.covid, aes(x = COVID_Stress1)) + geom_histogram(binwidth
= 1,
    colour = "black", fill = "gray") + xlab("Pandemic-related Stressors") + y
lab("Count") +
    theme_bw() + ggtitle("Wave 1") + scale_x_continuous(breaks = 0:9) + ylim(
0, 50)
splot2 <- ggplot(dt.covid, aes(x = COVID_Stress2)) + geom_histogram(binwidth
= 1,
    colour = "black", fill = "gray") + xlab("Pandemic-related Stressors") + y
lab("Count") +
    theme_bw() + ggtitle("Wave 2") + scale_x_continuous(breaks = 0:9) + ylim(
```

```
0, 50)
splot1 + splot2
```

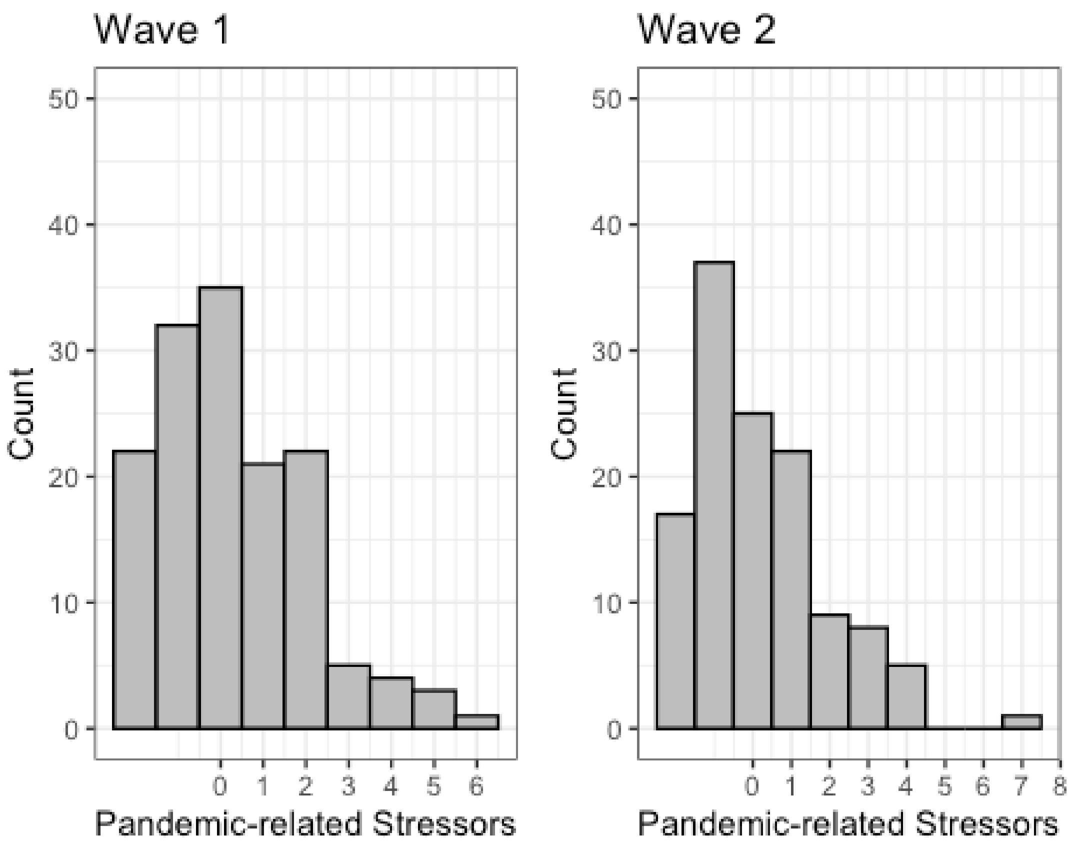

Pandemic-related Stress and Internalizing problems

```
# Wave 1
cor(dt.covid$SDQ_C_INT1, dt.covid$COVID_Stress1, use = "complete.obs")

## [1] 0.4555541

summary(lm(SDQ_C_INT1 ~ COVID_Stress1 + YINTERNAL + SEX + Covid_Age, data = d
t.covid))

##
## Call:
## lm(formula = SDQ_C_INT1 ~ COVID_Stress1 + YINTERNAL + SEX + Covid_Age,
##     data = dt.covid)
##
## Residuals:
##      Min       1Q   Median       3Q      Max
## -0.41147 -0.10494 -0.03267  0.08569  0.58262
##
## Coefficients:
##                Estimate Std. Error t value Pr(>|t|)
```

```
## (Intercept)   0.0811585  0.4674907   0.174  0.86243    
## COVID_Stress1 0.0521360  0.0084197   6.192 6.38e-09 ***
## YINTERNAL     0.2092713  0.0706392   2.963  0.00359 ** 
## SEX           0.1414366  0.0318165   4.445 1.79e-05 ***
## Covid_Age     0.0006465  0.0323292   0.020  0.98407    
## ---
## Signif. codes:  0 '***' 0.001 '**' 0.01 '*' 0.05 '.' 0.1 ' ' 1
## 
## Residual standard error: 0.1742 on 138 degrees of freedom
##   (2 observations deleted due to missingness)
## Multiple R-squared:  0.3164, Adjusted R-squared:  0.2966 
## F-statistic: 15.97 on 4 and 138 DF,  p-value: 9.074e-11
```

```
lm.beta(lm(SDQ_C_INT1 ~ COVID_Stress1 + YINTERNAL + SEX + Covid_Age, data = d
t.covid))
```

```
## 
## Call:
## lm(formula = SDQ_C_INT1 ~ COVID_Stress1 + YINTERNAL + SEX + Covid_Age, 
##     data = dt.covid)
## 
## Standardized Coefficients::
##   (Intercept) COVID_Stress1     YINTERNAL           SEX     Covid_Age 
##   0.000000000   0.437111518   0.225044983   0.338703641   0.001426568
```

```
# Wave 2
cor(dt.covid$SDQ_C_INT2, dt.covid$COVID_Stress2, use = "complete.obs")
```

```
## [1] 0.3916019
```

```
summary(lm(SDQ_C_INT2 ~ COVID_Stress2 + SDQ_C_INT + SEX + Covid_Age2, data = 
dt.covid))
```

```
## 
## Call:
## lm(formula = SDQ_C_INT2 ~ COVID_Stress2 + SDQ_C_INT + SEX + Covid_Age2, 
##     data = dt.covid)
## 
## Residuals:
##      Min       1Q   Median       3Q      Max 
## -0.39112 -0.13486 -0.03082  0.13589  0.65786 
## 
## Coefficients:
##                Estimate Std. Error t value Pr(>|t|)    
## (Intercept)    0.779504   0.613657   1.270  0.20662    
## COVID_Stress2  0.034714   0.011839   2.932  0.00408 ** 
## SDQ_C_INT      0.037915   0.006429   5.898 3.98e-08 ***
## SEX            0.077285   0.041340   1.870  0.06416 .  
## Covid_Age2    -0.039952   0.041286  -0.968  0.33529    
## ---
## Signif. codes:  0 '***' 0.001 '**' 0.01 '*' 0.05 '.' 0.1 ' ' 1
```

```
##
## Residual standard error: 0.2065 on 112 degrees of freedom
##   (28 observations deleted due to missingness)
## Multiple R-squared:  0.4169, Adjusted R-squared:  0.3961
## F-statistic: 20.02 on 4 and 112 DF,  p-value: 1.848e-12
```

```
lm.beta(lm(SDQ_C_INT2 ~ COVID_Stress2 + SDQ_C_INT + SEX + Covid_Age2, data =
dt.covid))
```

```
##
## Call:
## lm(formula = SDQ_C_INT2 ~ COVID_Stress2 + SDQ_C_INT + SEX + Covid_Age2,
##     data = dt.covid)
##
## Standardized Coefficients::
##   (Intercept) COVID_Stress2     SDQ_C_INT           SEX    Covid_Age2
##     0.0000000     0.2249709     0.4785669     0.1441276    -0.0711411
```

Residuals normal?

```
hist(residuals(lm(SDQ_C_INT1 ~ COVID_Stress1 + YINTERNAL + SEX + Covid_Age, d
ata = dt.covid)))
```

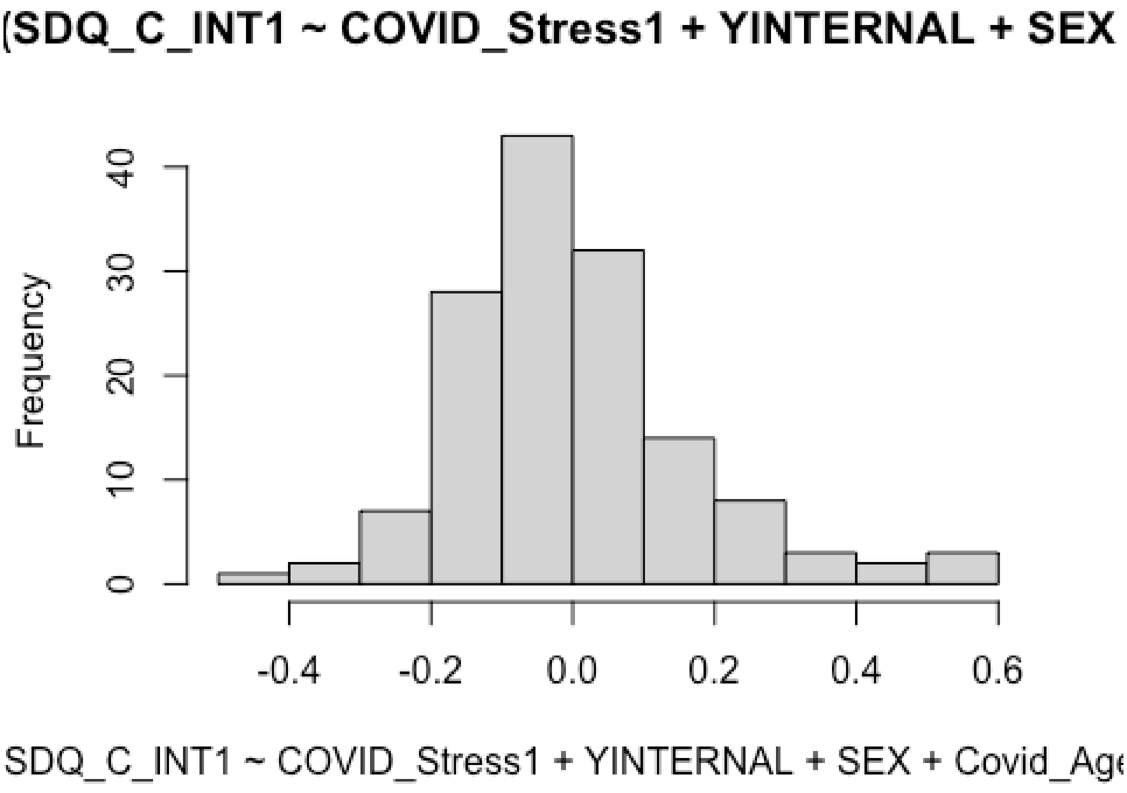

```
hist(residuals(lm(SDQ_C_INT2 ~ COVID_Stress2 + YINTERNAL + SEX + Covid_Age, d
ata = dt.covid)))
```

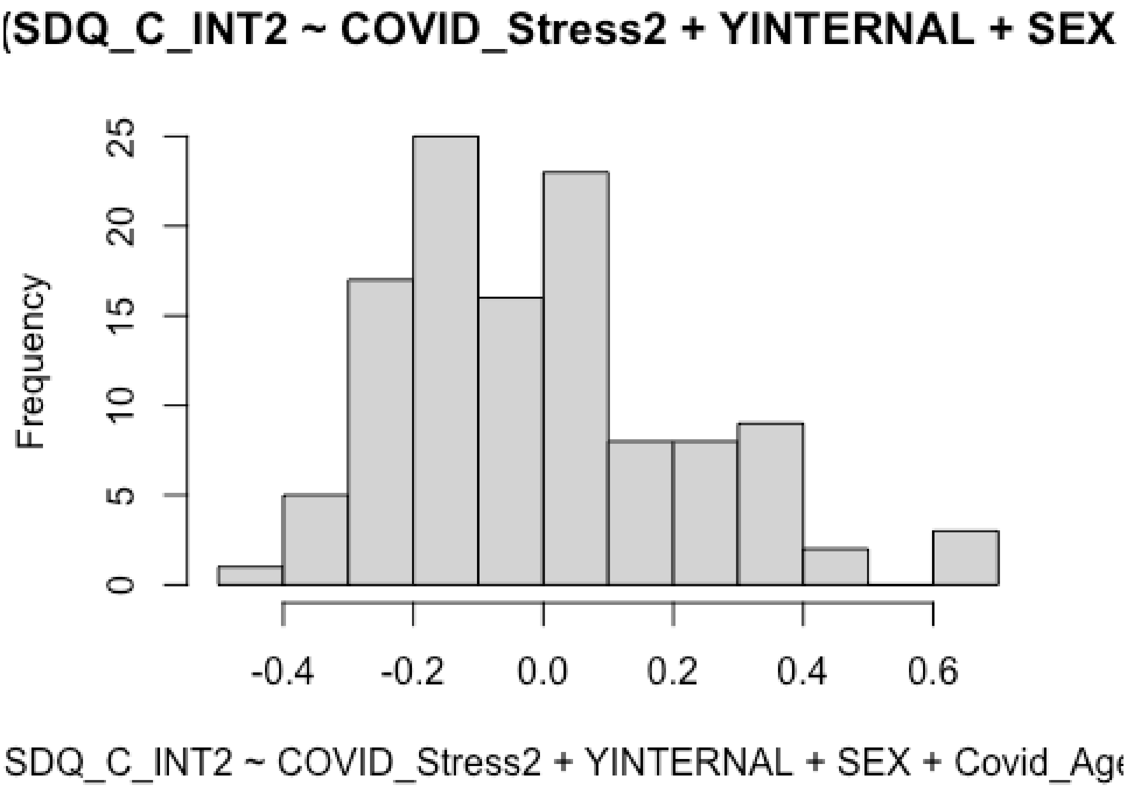

#Figure 1

```
isplot1 <- ggplot(dt.covid, aes(x = COVID_Stress1, y = SDQ_C_INT1))
isplot1 <- isplot1 + geom_jitter(width = 0.25, height = 0.25) + theme_classic
() +
    xlab("Pandemic-related Stressors") + ylab("Internalizing Problems") + geo
m_smooth(method = "lm") +
    scale_x_continuous(breaks = c(0, 2, 4, 6, 8))
isplot2 <- ggplot(dt.covid, aes(x = COVID_Stress2, y = SDQ_C_INT2))
isplot2 <- isplot2 + geom_jitter(width = 0.25, height = 0.25) + theme_classic
() +
    xlab("Pandemic-related Stressors") + ylab("Internalizing Problems") + geo
m_smooth(method = "lm") +
    scale_x_continuous(breaks = c(0, 2, 4, 6, 8))
isplot1 + isplot2
```

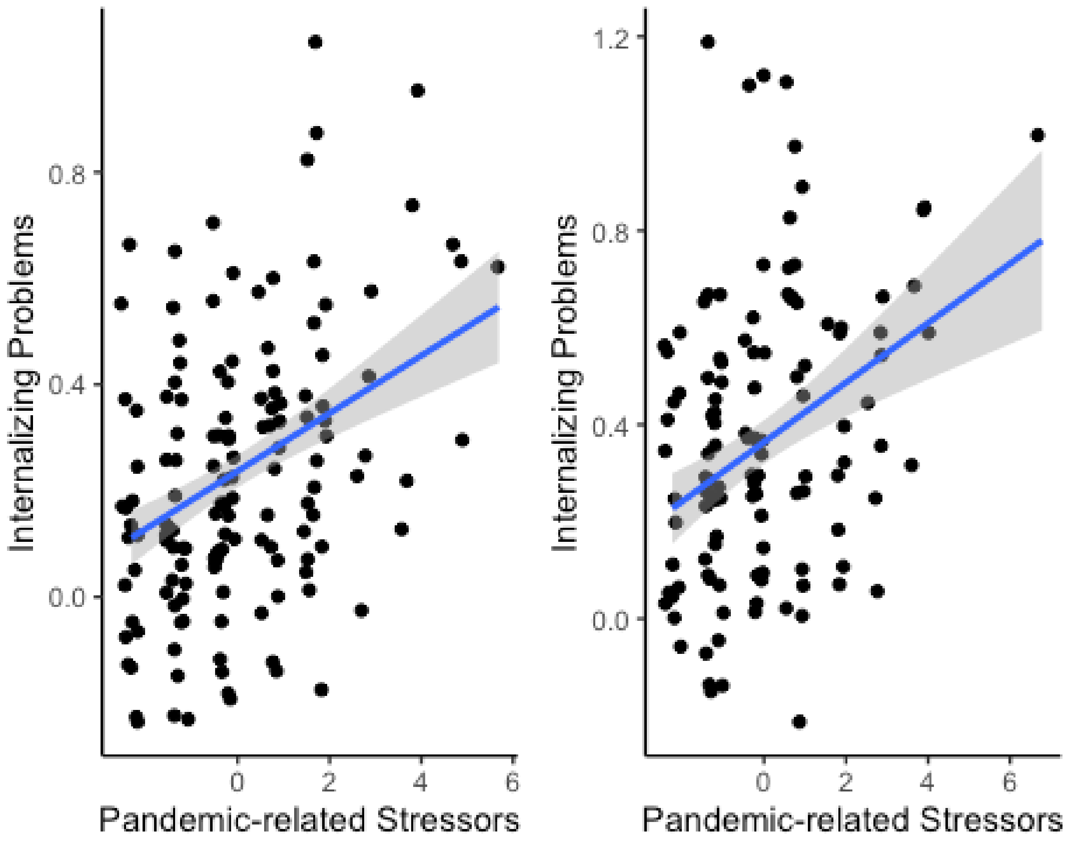

Emotion Regulation Models

```
#Emotion Regulation
#Rumination main effects
RumMod1 <- '
# regressions
SDQ_C_INT2 ~ SDQ_C_INT1+Covid_Age2+CRSQ_RUMc+SEX
SDQ_C_INT1 ~ YINTERNAL + CRSQ_RUMc + SEX + Covid_Age
Covid_Age2 ~ Covid_Age

# variances
SDQ_C_INT2 ~~ SDQ_C_INT2
SDQ_C_INT1 ~~ SDQ_C_INT1
YINTERNAL ~~ YINTERNAL
CRSQ_RUMc~~CRSQ_RUMc
SEX ~~ SEX
Covid_Age~~Covid_Age
Covid_Age2~~Covid_Age2

# covariances
YINTERNAL~~CRSQ_RUMc
YINTERNAL~~SEX
SEX~~CRSQ_RUMc
```

```
Covid_Age~~CRSQ_RUMc
Covid_Age2~~CRSQ_RUMc
Covid_Age~~SEX
Covid_Age2~~SEX
Covid_Age~~YINTERNAL
Covid_Age2~~SDQ_C_INT1

# intercepts
SDQ_C_INT2 ~1
SDQ_C_INT1 ~1
YINTERNAL ~1
CRSQ_RUMc~1
SEX ~1
Covid_Age~1
Covid_Age2~1
'

RumMod1.fit <- sem(RumMod1, estimator = "ML", missing ="ML", data = dt.covid)
summary(RumMod1.fit, standardized=TRUE, fit.measures = TRUE)

## lavaan 0.6-6 ended normally after 146 iterations
##
##   Estimator                                         ML
##   Optimization method                           NLMINB
##   Number of free parameters                         32
##
##   Number of observations                           145
##   Number of missing patterns                         4
##
## Model Test User Model:
##
##   Test statistic                                 3.471
##   Degrees of freedom                                 3
##   P-value (Chi-square)                           0.324
##
## Model Test Baseline Model:
##
##   Test statistic                               717.687
##   Degrees of freedom                                21
##   P-value                                        0.000
##
## User Model versus Baseline Model:
##
##   Comparative Fit Index (CFI)                    0.999
##   Tucker-Lewis Index (TLI)                       0.995
##
## Loglikelihood and Information Criteria:
##
##   Loglikelihood user model (H0)               -366.776
##   Loglikelihood unrestricted model (H1)       -365.040
```

```
##
##   Akaike (AIC)                                  797.552
##   Bayesian (BIC)                                892.808
##   Sample-size adjusted Bayesian (BIC)           791.548
##
## Root Mean Square Error of Approximation:
##
##   RMSEA                                           0.033
##   90 Percent confidence interval - lower          0.000
##   90 Percent confidence interval - upper          0.148
##   P-value RMSEA <= 0.05                           0.475
##
## Standardized Root Mean Square Residual:
##
##   SRMR                                            0.006
##
## Parameter Estimates:
##
##   Standard errors                              Standard
##   Information                                  Observed
##   Observed information based on                 Hessian
##
## Regressions:
##                    Estimate  Std.Err  z-value  P(>|z|)   Std.lv  Std.all
##   SDQ_C_INT2 ~
##     SDQ_C_INT1        0.696    0.104    6.686    0.000    0.696    0.545
##     Covid_Age2       -0.054    0.041   -1.307    0.191   -0.054   -0.094
##     CRSQ_RUMc         0.001    0.003    0.265    0.791    0.001    0.021
##     SEX               0.082    0.043    1.901    0.057    0.082    0.154
##   SDQ_C_INT1 ~
##     YINTERNAL         0.134    0.087    1.549    0.121    0.134    0.145
##     CRSQ_RUMc         0.007    0.003    2.359    0.018    0.007    0.209
##     SEX               0.154    0.035    4.450    0.000    0.154    0.367
##     Covid_Age        -0.015    0.035   -0.434    0.664   -0.015   -0.034
##   Covid_Age2 ~
##     Covid_Age         1.005    0.009  113.109    0.000    1.005    0.995
##
## Covariances:
##                    Estimate  Std.Err  z-value  P(>|z|)   Std.lv  Std.all
##   CRSQ_RUMc ~~
##     YINTERNAL         0.703    0.130    5.428    0.000    0.703    0.506
##   SEX ~~
##     YINTERNAL        -0.041    0.010   -4.201    0.000   -0.041   -0.374
##   CRSQ_RUMc ~~
##     SEX              -0.489    0.259   -1.884    0.060   -0.489   -0.159
##     Covid_Age         0.004    0.237    0.018    0.986    0.004    0.001
##  .Covid_Age2 ~~
##     CRSQ_RUMc         0.018    0.022    0.795    0.427    0.018    0.063
##   SEX ~~
##     Covid_Age         0.032    0.019    1.663    0.096    0.032    0.139
```

```
##   .Covid_Age2 ~~
##      SEX               0.001    0.002    0.354    0.723    0.001    0.031
##    YINTERNAL ~~
##      Covid_Age        -0.015    0.009   -1.712    0.087   -0.015   -0.144
##   .SDQ_C_INT1 ~~
##     .Covid_Age2        0.002    0.001    2.721    0.007    0.002    0.266
##
## Intercepts:
##                     Estimate  Std.Err  z-value  P(>|z|)   Std.lv  Std.all
##     .SDQ_C_INT2        0.962    0.613    1.570    0.116    0.962    3.623
##     .SDQ_C_INT1        0.336    0.512    0.657    0.511    0.336    1.616
##      YINTERNAL         0.410    0.019   22.074    0.000    0.410    1.833
##      CRSQ_RUMc         0.007    0.516    0.014    0.989    0.007    0.001
##      SEX               0.434    0.041   10.565    0.000    0.434    0.877
##      Covid_Age        14.375    0.038  375.987    0.000   14.375   31.226
##     .Covid_Age2        0.509    0.128    3.987    0.000    0.509    1.096
##
## Variances:
##                     Estimate  Std.Err  z-value  P(>|z|)   Std.lv  Std.all
##     .SDQ_C_INT2        0.044    0.006    7.670    0.000    0.044    0.626
##     .SDQ_C_INT1        0.036    0.004    8.448    0.000    0.036    0.835
##      YINTERNAL         0.050    0.006    8.515    0.000    0.050    1.000
##      CRSQ_RUMc        38.564    4.551    8.473    0.000   38.564    1.000
##      SEX               0.245    0.029    8.521    0.000    0.245    1.000
##      Covid_Age         0.212    0.025    8.512    0.000    0.212    1.000
##     .Covid_Age2        0.002    0.000    7.653    0.000    0.002    0.009
```

```
#Rumination, interaction with pandemic-related stress
dt.covid$RumxStress1<-dt.covid$CRSQ_RUMc*dt.covid$COVID_Stress1
dt.covid$RumxStress2<-dt.covid$CRSQ_RUMc*dt.covid$COVID_Stress2
RumMod2 <- '
# regressions
SDQ_C_INT2 ~ SDQ_C_INT1+Covid_Age2+CRSQ_RUMc+COVID_Stress2+RumxStress2+SEX
SDQ_C_INT1 ~ YINTERNAL + CRSQ_RUMc + SEX + Covid_Age+COVID_Stress1+RumxStress
1
Covid_Age2 ~ Covid_Age
COVID_Stress2~COVID_Stress1
RumxStress2~RumxStress1

# variances
SDQ_C_INT2 ~~ SDQ_C_INT2
SDQ_C_INT1 ~~ SDQ_C_INT1
YINTERNAL ~~ YINTERNAL
CRSQ_RUMc~~CRSQ_RUMc
SEX ~~ SEX
Covid_Age~~Covid_Age
Covid_Age2~~Covid_Age2
COVID_Stress1~~COVID_Stress1
COVID_Stress2~~COVID_Stress2
RumxStress1~~RumxStress1
```

```
RumxStress2~~RumxStress2

# covariances
YINTERNAL~~CRSQ_RUMc
YINTERNAL~~SEX
SEX~~CRSQ_RUMc
Covid_Age~~CRSQ_RUMc
Covid_Age2~~CRSQ_RUMc
Covid_Age~~SEX
Covid_Age2~~SEX
Covid_Age~~YINTERNAL
Covid_Age2~~SDQ_C_INT1
COVID_Stress1~~YINTERNAL
COVID_Stress1~~SEX
COVID_Stress1~~CRSQ_RUMc
COVID_Stress1~~Covid_Age
COVID_Stress2~~SDQ_C_INT1
COVID_Stress2~~Covid_Age2
COVID_Stress2~~CRSQ_RUMc
COVID_Stress2~~SEX
COVID_Stress1~~RumxStress1
RumxStress1~~YINTERNAL
RumxStress1~~SEX
RumxStress1~~CRSQ_RUMc
RumxStress1~~Covid_Age
COVID_Stress2~~RumxStress2
RumxStress2~~SDQ_C_INT1
RumxStress2~~Covid_Age2
RumxStress2~~CRSQ_RUMc
RumxStress2~~SEX

# intercepts
SDQ_C_INT2 ~1
SDQ_C_INT1 ~1
YINTERNAL ~1
CRSQ_RUMc~1
SEX ~1
Covid_Age~1
Covid_Age2~1
COVID_Stress1~1
COVID_Stress2~1
RumxStress1~1
RumxStress2~1
'
RumMod2.fit <- sem(RumMod2, estimator = "ML", missing ="ML", data = dt.covid)
summary(RumMod2.fit, standardized=TRUE,fit.measures = TRUE)

## lavaan 0.6-6 ended normally after 262 iterations
##
##   Estimator                                         ML
```

```
##   Optimization method                           NLMINB
##   Number of free parameters                         64
##
##   Number of observations                           145
##   Number of missing patterns                         5
##
## Model Test User Model:
##
##   Test statistic                                16.176
##   Degrees of freedom                                13
##   P-value (Chi-square)                           0.240
##
## Model Test Baseline Model:
##
##   Test statistic                               863.794
##   Degrees of freedom                                55
##   P-value                                        0.000
##
## User Model versus Baseline Model:
##
##   Comparative Fit Index (CFI)                    0.996
##   Tucker-Lewis Index (TLI)                       0.983
##
## Loglikelihood and Information Criteria:
##
##   Loglikelihood user model (H0)              -1844.147
##   Loglikelihood unrestricted model (H1)      -1836.059
##
##   Akaike (AIC)                                3816.295
##   Bayesian (BIC)                              4006.806
##   Sample-size adjusted Bayesian (BIC)         3804.287
##
## Root Mean Square Error of Approximation:
##
##   RMSEA                                          0.041
##   90 Percent confidence interval - lower         0.000
##   90 Percent confidence interval - upper         0.097
##   P-value RMSEA <= 0.05                          0.545
##
## Standardized Root Mean Square Residual:
##
##   SRMR                                           0.036
##
## Parameter Estimates:
##
##   Standard errors                             Standard
##   Information                                 Observed
##   Observed information based on                Hessian
##
## Regressions:
```

```
##                      Estimate  Std.Err  z-value  P(>|z|)   Std.lv  Std.all
##   SDQ_C_INT2 ~
##     SDQ_C_INT1          0.591    0.107    5.497    0.000    0.591    0.460
##     Covid_Age2         -0.041    0.040   -1.030    0.303   -0.041   -0.073
##     CRSQ_RUMc           0.002    0.003    0.652    0.514    0.002    0.051
##     COVID_Stress2       0.034    0.012    2.815    0.005    0.034    0.220
##     RumxStress2        -0.001    0.002   -0.617    0.537   -0.001   -0.046
##     SEX                 0.095    0.042    2.250    0.024    0.095    0.178
##   SDQ_C_INT1 ~
##     YINTERNAL           0.132    0.078    1.698    0.089    0.132    0.144
##     CRSQ_RUMc           0.006    0.003    2.388    0.017    0.006    0.192
##     SEX                 0.144    0.031    4.633    0.000    0.144    0.346
##     Covid_Age           0.006    0.031    0.192    0.847    0.006    0.014
##     COVID_Stress1       0.047    0.008    5.856    0.000    0.047    0.401
##     RumxStress1        -0.000    0.001   -0.200    0.842   -0.000   -0.013
##   Covid_Age2 ~
##     Covid_Age           1.009    0.009  113.608    0.000    1.009    0.995
##   COVID_Stress2 ~
##     COVID_Stress1       0.399    0.075    5.310    0.000    0.399    0.409
##   RumxStress2 ~
##     RumxStress1         0.446    0.059    7.497    0.000    0.446    0.537
##
## Covariances:
##                      Estimate  Std.Err  z-value  P(>|z|)   Std.lv  Std.all
##   CRSQ_RUMc ~~
##     YINTERNAL           0.718    0.131    5.470    0.000    0.718    0.513
##   SEX ~~
##     YINTERNAL          -0.041    0.010   -4.140    0.000   -0.041   -0.366
##   CRSQ_RUMc ~~
##     SEX                -0.483    0.263   -1.838    0.066   -0.483   -0.156
##     Covid_Age           0.014    0.241    0.060    0.952    0.014    0.005
##  .Covid_Age2 ~~
##     CRSQ_RUMc           0.017    0.022    0.747    0.455    0.017    0.060
##   SEX ~~
##     Covid_Age           0.038    0.019    1.962    0.050    0.038    0.165
##  .Covid_Age2 ~~
##     SEX                 0.001    0.002    0.374    0.708    0.001    0.033
##   YINTERNAL ~~
##     Covid_Age          -0.015    0.009   -1.712    0.087   -0.015   -0.144
##  .SDQ_C_INT1 ~~
##    .Covid_Age2          0.002    0.001    2.156    0.031    0.002    0.206
##   YINTERNAL ~~
##     COVID_Stress1       0.001    0.032    0.045    0.964    0.001    0.004
##   SEX ~~
##     COVID_Stress1       0.030    0.072    0.412    0.680    0.030    0.034
##   CRSQ_RUMc ~~
##     COVID_Stress1       0.222    0.905    0.245    0.806    0.222    0.020
##   Covid_Age ~~
##     COVID_Stress1      -0.026    0.066   -0.394    0.693   -0.026   -0.033
##  .SDQ_C_INT1 ~~
```

```
##     .COVID_Stress2      0.037    0.023    1.583    0.113    0.037    0.142
##   .Covid_Age2 ~~
##     .COVID_Stress2      0.009    0.006    1.439    0.150    0.009    0.135
##   .COVID_Stress2 ~~
##      CRSQ_RUMc         -0.361    0.757   -0.476    0.634   -0.361   -0.037
##      SEX                0.056    0.064    0.880    0.379    0.056    0.073
##    COVID_Stress1 ~~
##      RumxStress1        0.222    1.663    0.133    0.894    0.222    0.011
##    YINTERNAL ~~
##      RumxStress1       -0.104    0.215   -0.483    0.629   -0.104   -0.040
##    SEX ~~
##      RumxStress1       -0.438    0.475   -0.923    0.356   -0.438   -0.077
##    CRSQ_RUMc ~~
##      RumxStress1       -0.205    5.968   -0.034    0.973   -0.205   -0.003
##    Covid_Age ~~
##      RumxStress1        0.301    0.442    0.682    0.495    0.301    0.057
##   .COVID_Stress2 ~~
##     .RumxStress2       -3.795    1.173   -3.236    0.001   -3.795   -0.304
##   .SDQ_C_INT1 ~~
##     .RumxStress2        0.127    0.120    1.058    0.290    0.127    0.093
##   .Covid_Age2 ~~
##     .RumxStress2        0.021    0.034    0.615    0.539    0.021    0.058
##   .RumxStress2 ~~
##      CRSQ_RUMc          4.108    3.974    1.034    0.301    4.108    0.081
##      SEX                0.482    0.336    1.437    0.151    0.482    0.120
##
## Intercepts:
##                     Estimate  Std.Err  z-value  P(>|z|)   Std.lv  Std.all
##     .SDQ_C_INT2         0.799    0.595    1.341    0.180    0.799    3.028
##     .SDQ_C_INT1         0.036    0.455    0.079    0.937    0.036    0.174
##      YINTERNAL          0.410    0.019   22.074    0.000    0.410    1.833
##      CRSQ_RUMc          0.007    0.519    0.013    0.989    0.007    0.001
##      SEX                0.434    0.041   10.558    0.000    0.434    0.877
##      Covid_Age         14.375    0.038  375.933    0.000   14.375   31.222
##     .Covid_Age2         0.450    0.128    3.525    0.000    0.450    0.964
##      COVID_Stress1     -0.031    0.144   -0.215    0.829   -0.031   -0.018
##     .COVID_Stress2     -0.089    0.139   -0.637    0.524   -0.089   -0.052
##      RumxStress1        0.330    0.957    0.344    0.731    0.330    0.029
##     .RumxStress2        0.203    0.725    0.281    0.779    0.203    0.021
##
## Variances:
##                     Estimate  Std.Err  z-value  P(>|z|)   Std.lv  Std.all
##     .SDQ_C_INT2         0.041    0.005    7.677    0.000    0.041    0.588
##     .SDQ_C_INT1         0.028    0.003    8.400    0.000    0.028    0.675
##      YINTERNAL          0.050    0.006    8.515    0.000    0.050    1.000
##      CRSQ_RUMc         39.003    4.656    8.377    0.000   39.003    1.000
##      SEX                0.246    0.029    8.510    0.000    0.246    1.000
##      Covid_Age          0.212    0.025    8.512    0.000    0.212    1.000
##     .Covid_Age2         0.002    0.000    7.693    0.000    0.002    0.009
##      COVID_Stress1      3.017    0.354    8.515    0.000    3.017    1.000
```

```
##    .COVID_Stress2      2.394     0.303     7.888     0.000     2.394     0.833
##     RumxStress1      132.908    15.609     8.515     0.000   132.908     1.000
##    .RumxStress2       65.314     8.276     7.892     0.000    65.314     0.712

#Expressive Suppression main effects
ESMod1 <- '
# regressions
SDQ_C_INT2 ~ SDQ_C_INT1+Covid_Age2+ERQ_ESc+SEX
SDQ_C_INT1 ~ YINTERNAL + ERQ_ESc + SEX + Covid_Age
Covid_Age2 ~ Covid_Age

# variances
SDQ_C_INT2 ~~ SDQ_C_INT2
SDQ_C_INT1 ~~ SDQ_C_INT1
YINTERNAL ~~ YINTERNAL
ERQ_ESc~~ERQ_ESc
SEX ~~ SEX
Covid_Age~~Covid_Age
Covid_Age2~~Covid_Age2

# covariances
YINTERNAL~~ERQ_ESc
YINTERNAL~~SEX
SEX~~ERQ_ESc
Covid_Age~~ERQ_ESc
Covid_Age2~~ERQ_ESc
Covid_Age~~SEX
Covid_Age2~~SEX
Covid_Age~~YINTERNAL
Covid_Age2~~SDQ_C_INT1

# intercepts
SDQ_C_INT2 ~1
SDQ_C_INT1 ~1
YINTERNAL ~1
ERQ_ESc~1
SEX ~1
Covid_Age~1
Covid_Age2~1
'

ESMod1.fit <- sem(ESMod1, estimator = "ML", missing ="ML", data = dt.covid)
summary(ESMod1.fit, standardized=TRUE, fit.measures = TRUE)

## lavaan 0.6-6 ended normally after 129 iterations
##
##   Estimator                                         ML
##   Optimization method                           NLMINB
##   Number of free parameters                         32
##
```

```
##   Number of observations                           145
##   Number of missing patterns                         4
##
## Model Test User Model:
##
##   Test statistic                                 4.689
##   Degrees of freedom                                 3
##   P-value (Chi-square)                           0.196
##
## Model Test Baseline Model:
##
##   Test statistic                               693.464
##   Degrees of freedom                                21
##   P-value                                        0.000
##
## User Model versus Baseline Model:
##
##   Comparative Fit Index (CFI)                    0.997
##   Tucker-Lewis Index (TLI)                       0.982
##
## Loglikelihood and Information Criteria:
##
##   Loglikelihood user model (H0)                -62.017
##   Loglikelihood unrestricted model (H1)        -59.672
##
##   Akaike (AIC)                                 188.033
##   Bayesian (BIC)                               283.289
##   Sample-size adjusted Bayesian (BIC)          182.029
##
## Root Mean Square Error of Approximation:
##
##   RMSEA                                          0.062
##   90 Percent confidence interval - lower         0.000
##   90 Percent confidence interval - upper         0.165
##   P-value RMSEA <= 0.05                          0.333
##
## Standardized Root Mean Square Residual:
##
##   SRMR                                           0.006
##
## Parameter Estimates:
##
##   Standard errors                             Standard
##   Information                                 Observed
##   Observed information based on                Hessian
##
## Regressions:
##                    Estimate  Std.Err  z-value  P(>|z|)   Std.lv  Std.all
##   SDQ_C_INT2 ~
##     SDQ_C_INT1        0.636    0.101    6.301    0.000    0.636    0.498
```

```
##     Covid_Age2       -0.061    0.040   -1.515    0.130   -0.061   -0.107
##     ERQ_ESc           0.065    0.027    2.367    0.018    0.065    0.172
##     SEX               0.083    0.041    2.024    0.043    0.083    0.155
##   SDQ_C_INT1 ~
##     YINTERNAL         0.212    0.074    2.883    0.004    0.212    0.229
##     ERQ_ESc           0.078    0.022    3.496    0.000    0.078    0.265
##     SEX               0.144    0.034    4.220    0.000    0.144    0.342
##     Covid_Age        -0.019    0.034   -0.564    0.573   -0.019   -0.043
##   Covid_Age2 ~
##     Covid_Age         1.005    0.009  113.440    0.000    1.005    0.995
##
## Covariances:
##                    Estimate  Std.Err  z-value  P(>|z|)   Std.lv  Std.all
##   ERQ_ESc ~~
##     YINTERNAL         0.006    0.013    0.468    0.640    0.006    0.039
##   SEX ~~
##     YINTERNAL        -0.041    0.010   -4.204    0.000   -0.041   -0.374
##   ERQ_ESc ~~
##     SEX               0.031    0.029    1.074    0.283    0.031    0.090
##     Covid_Age         0.032    0.027    1.164    0.244    0.032    0.098
##  .Covid_Age2 ~~
##     ERQ_ESc          -0.002    0.003   -0.656    0.512   -0.002   -0.058
##   SEX ~~
##     Covid_Age         0.032    0.019    1.664    0.096    0.032    0.140
##  .Covid_Age2 ~~
##     SEX               0.001    0.002    0.335    0.738    0.001    0.029
##   YINTERNAL ~~
##     Covid_Age        -0.015    0.009   -1.712    0.087   -0.015   -0.144
##  .SDQ_C_INT1 ~~
##    .Covid_Age2        0.003    0.001    3.106    0.002    0.003    0.303
##
## Intercepts:
##                    Estimate  Std.Err  z-value  P(>|z|)   Std.lv  Std.all
##    .SDQ_C_INT2        1.086    0.602    1.806    0.071    1.086    4.091
##    .SDQ_C_INT1        0.368    0.499    0.737    0.461    0.368    1.770
##     YINTERNAL         0.410    0.019   22.074    0.000    0.410    1.833
##     ERQ_ESc           0.004    0.059    0.060    0.952    0.004    0.005
##     SEX               0.434    0.041   10.564    0.000    0.434    0.877
##     Covid_Age        14.375    0.038  375.995    0.000   14.375   31.227
##    .Covid_Age2        0.512    0.127    4.020    0.000    0.512    1.102
##
## Variances:
##                    Estimate  Std.Err  z-value  P(>|z|)   Std.lv  Std.all
##    .SDQ_C_INT2        0.042    0.006    7.662    0.000    0.042    0.599
##    .SDQ_C_INT1        0.035    0.004    8.444    0.000    0.035    0.800
##     YINTERNAL         0.050    0.006    8.515    0.000    0.050    1.000
##     ERQ_ESc           0.496    0.059    8.451    0.000    0.496    1.000
##     SEX               0.245    0.029    8.520    0.000    0.245    1.000
##     Covid_Age         0.212    0.025    8.512    0.000    0.212    1.000
##    .Covid_Age2        0.002    0.000    7.673    0.000    0.002    0.009
```

```
#Expressive Suppression, interaction with pandemic-related stress
dt.covid$ESxStress1<-dt.covid$ERQ_ESc*dt.covid$COVID_Stress1
dt.covid$ESxStress2<-dt.covid$ERQ_ESc*dt.covid$COVID_Stress2
ESMod2 <- '
# regressions
SDQ_C_INT2 ~ SDQ_C_INT1+Covid_Age2+ERQ_ESc+COVID_Stress2+ESxStress2+SEX
SDQ_C_INT1 ~ YINTERNAL + ERQ_ESc + SEX + Covid_Age+COVID_Stress1+ESxStress1
Covid_Age2 ~ Covid_Age
COVID_Stress2~COVID_Stress1
ESxStress2~ESxStress1

# variances
SDQ_C_INT2 ~~ SDQ_C_INT2
SDQ_C_INT1 ~~ SDQ_C_INT1
YINTERNAL ~~ YINTERNAL
ERQ_ESc~~ERQ_ESc
SEX ~~ SEX
Covid_Age~~Covid_Age
Covid_Age2~~Covid_Age2
COVID_Stress1~~COVID_Stress1
COVID_Stress2~~COVID_Stress2
ESxStress1~~ESxStress1
ESxStress2~~ESxStress2

# covariances
YINTERNAL~~ERQ_ESc
YINTERNAL~~SEX
SEX~~ERQ_ESc
Covid_Age~~ERQ_ESc
Covid_Age2~~ERQ_ESc
Covid_Age~~SEX
Covid_Age2~~SEX
Covid_Age~~YINTERNAL
Covid_Age2~~SDQ_C_INT1
COVID_Stress1~~YINTERNAL
COVID_Stress1~~SEX
COVID_Stress1~~ERQ_ESc
COVID_Stress1~~Covid_Age
COVID_Stress2~~SDQ_C_INT1
COVID_Stress2~~Covid_Age2
COVID_Stress2~~ERQ_ESc
COVID_Stress2~~SEX
COVID_Stress1~~ESxStress1
ESxStress1~~YINTERNAL
ESxStress1~~SEX
ESxStress1~~ERQ_ESc
ESxStress1~~Covid_Age
COVID_Stress2~~ESxStress2
ESxStress2~~SDQ_C_INT1
ESxStress2~~Covid_Age2
```

```
ESxStress2~~ERQ_ESc
ESxStress2~~SEX

# intercepts
SDQ_C_INT2 ~1
SDQ_C_INT1 ~1
YINTERNAL ~1
ERQ_ESc~1
SEX ~1
Covid_Age~1
Covid_Age2~1
COVID_Stress1~1
COVID_Stress2~1
ESxStress1~1
ESxStress2~1
'
ESMod2.fit <- sem(ESMod2, estimator = "ML", missing ="ML", data = dt.covid)
summary(ESMod2.fit, standardized=TRUE, fit.measures = TRUE)

## lavaan 0.6-6 ended normally after 162 iterations
##
##   Estimator                                         ML
##   Optimization method                           NLMINB
##   Number of free parameters                         64
##
##   Number of observations                           145
##   Number of missing patterns                         5
##
## Model Test User Model:
##
##   Test statistic                                20.823
##   Degrees of freedom                                13
##   P-value (Chi-square)                           0.077
##
## Model Test Baseline Model:
##
##   Test statistic                               848.629
##   Degrees of freedom                                55
##   P-value                                        0.000
##
## User Model versus Baseline Model:
##
##   Comparative Fit Index (CFI)                    0.990
##   Tucker-Lewis Index (TLI)                       0.958
##
## Loglikelihood and Information Criteria:
##
##   Loglikelihood user model (H0)               -972.158
##   Loglikelihood unrestricted model (H1)       -961.746
##
```

```
##   Akaike (AIC)                                2072.315
##   Bayesian (BIC)                              2262.826
##   Sample-size adjusted Bayesian (BIC)         2060.308
##
## Root Mean Square Error of Approximation:
##
##   RMSEA                                          0.064
##   90 Percent confidence interval - lower         0.000
##   90 Percent confidence interval - upper         0.114
##   P-value RMSEA <= 0.05                          0.288
##
## Standardized Root Mean Square Residual:
##
##   SRMR                                           0.039
##
## Parameter Estimates:
##
##   Standard errors                             Standard
##   Information                                 Observed
##   Observed information based on                Hessian
##
## Regressions:
##                    Estimate  Std.Err  z-value  P(>|z|)   Std.lv  Std.all
##   SDQ_C_INT2 ~
##     SDQ_C_INT1        0.542    0.103    5.263    0.000    0.542    0.421
##     Covid_Age2       -0.047    0.039   -1.180    0.238   -0.047   -0.083
##     ERQ_ESc           0.060    0.027    2.260    0.024    0.060    0.162
##     COVID_Stress2     0.034    0.012    2.741    0.006    0.034    0.214
##     ESxStress2        0.001    0.014    0.068    0.946    0.001    0.005
##     SEX               0.089    0.040    2.239    0.025    0.089    0.168
##   SDQ_C_INT1 ~
##     YINTERNAL         0.208    0.066    3.170    0.002    0.208    0.228
##     ERQ_ESc           0.064    0.020    3.257    0.001    0.064    0.221
##     SEX               0.130    0.030    4.356    0.000    0.130    0.315
##     Covid_Age        -0.001    0.030   -0.020    0.984   -0.001   -0.001
##     COVID_Stress1     0.047    0.008    6.004    0.000    0.047    0.402
##     ESxStress1        0.023    0.011    2.014    0.044    0.023    0.136
##   Covid_Age2 ~
##     Covid_Age         1.008    0.009  114.322    0.000    1.008    0.995
##   COVID_Stress2 ~
##     COVID_Stress1     0.358    0.075    4.792    0.000    0.358    0.372
##   ESxStress2 ~
##     ESxStress1        0.580    0.085    6.794    0.000    0.580    0.488
##
## Covariances:
##                    Estimate  Std.Err  z-value  P(>|z|)   Std.lv  Std.all
##   ERQ_ESc ~~
##     YINTERNAL         0.003    0.013    0.255    0.799    0.003    0.021
##   SEX ~~
##     YINTERNAL        -0.042    0.010   -4.224    0.000   -0.042   -0.376
```

```
##   ERQ_ESc ~~
##     SEX               0.035    0.029    1.172    0.241    0.035    0.099
##     Covid_Age         0.036    0.027    1.302    0.193    0.036    0.110
##  .Covid_Age2 ~~
##     ERQ_ESc          -0.003    0.003   -0.975    0.330   -0.003   -0.089
##   SEX ~~
##     Covid_Age         0.034    0.019    1.768    0.077    0.034    0.149
##  .Covid_Age2 ~~
##     SEX               0.000    0.002    0.209    0.834    0.000    0.019
##   YINTERNAL ~~
##     Covid_Age        -0.015    0.009   -1.713    0.087   -0.015   -0.144
##  .SDQ_C_INT1 ~~
##    .Covid_Age2        0.001    0.001    2.132    0.033    0.001    0.204
##   YINTERNAL ~~
##     COVID_Stress1     0.001    0.032    0.045    0.964    0.001    0.004
##   SEX ~~
##     COVID_Stress1     0.039    0.072    0.533    0.594    0.039    0.045
##   ERQ_ESc ~~
##     COVID_Stress1     0.125    0.104    1.212    0.226    0.125    0.103
##   Covid_Age ~~
##     COVID_Stress1    -0.026    0.066   -0.398    0.691   -0.026   -0.033
##  .SDQ_C_INT1 ~~
##    .COVID_Stress2     0.020    0.022    0.889    0.374    0.020    0.079
##  .Covid_Age2 ~~
##    .COVID_Stress2     0.010    0.006    1.518    0.129    0.010    0.142
##  .COVID_Stress2 ~~
##     ERQ_ESc           0.120    0.096    1.253    0.210    0.120    0.110
##     SEX               0.049    0.065    0.759    0.448    0.049    0.064
##   COVID_Stress1 ~~
##     ESxStress1       -0.065    0.176   -0.372    0.710   -0.065   -0.031
##   YINTERNAL ~~
##     ESxStress1       -0.028    0.023   -1.221    0.222   -0.028   -0.103
##   SEX ~~
##     ESxStress1        0.049    0.051    0.963    0.335    0.049    0.083
##   ERQ_ESc ~~
##     ESxStress1        0.022    0.072    0.302    0.763    0.022    0.026
##   Covid_Age ~~
##     ESxStress1       -0.032    0.047   -0.684    0.494   -0.032   -0.058
##  .COVID_Stress2 ~~
##    .ESxStress2        0.703    0.188    3.738    0.000    0.703    0.362
##  .SDQ_C_INT1 ~~
##    .ESxStress2       -0.007    0.018   -0.392    0.695   -0.007   -0.035
##  .Covid_Age2 ~~
##    .ESxStress2        0.005    0.005    1.034    0.301    0.005    0.096
##  .ESxStress2 ~~
##     ERQ_ESc           0.090    0.077    1.164    0.244    0.090    0.101
##     SEX              -0.041    0.052   -0.795    0.427   -0.041   -0.067
##
## Intercepts:
##                    Estimate  Std.Err  z-value  P(>|z|)   Std.lv  Std.all
```

```
##     .SDQ_C_INT2        0.891    0.587    1.518    0.129    0.891    3.392
##     .SDQ_C_INT1        0.102    0.438    0.234    0.815    0.102    0.501
##      YINTERNAL         0.410    0.019   22.074    0.000    0.410    1.833
##      ERQ_ESc           0.003    0.059    0.059    0.953    0.003    0.005
##      SEX               0.434    0.041   10.546    0.000    0.434    0.876
##      Covid_Age        14.375    0.038  375.975    0.000   14.375   31.225
##     .Covid_Age2        0.466    0.127    3.674    0.000    0.466    0.999
##      COVID_Stress1    -0.031    0.144   -0.215    0.829   -0.031   -0.018
##     .COVID_Stress2    -0.078    0.139   -0.559    0.576   -0.078   -0.047
##      ESxStress1        0.130    0.101    1.290    0.197    0.130    0.108
##     .ESxStress2        0.098    0.114    0.860    0.390    0.098    0.068
##
## Variances:
##                     Estimate  Std.Err  z-value  P(>|z|)   Std.lv  Std.all
##     .SDQ_C_INT2        0.040    0.005    7.663    0.000    0.040    0.574
##     .SDQ_C_INT1        0.026    0.003    8.414    0.000    0.026    0.629
##      YINTERNAL         0.050    0.006    8.515    0.000    0.050    1.000
##      ERQ_ESc           0.497    0.059    8.422    0.000    0.497    1.000
##      SEX               0.246    0.029    8.486    0.000    0.246    1.000
##      Covid_Age         0.212    0.025    8.512    0.000    0.212    1.000
##     .Covid_Age2        0.002    0.000    7.723    0.000    0.002    0.009
##      COVID_Stress1     3.017    0.354    8.515    0.000    3.017    1.000
##     .COVID_Stress2     2.401    0.305    7.866    0.000    2.401    0.861
##      ESxStress1        1.459    0.173    8.452    0.000    1.459    1.000
##     .ESxStress2        1.569    0.201    7.809    0.000    1.569    0.762
```

```
#Cognitive Reappraisal main effects
CRMod1 <- '
# regressions
SDQ_C_INT2 ~ SDQ_C_INT1+Covid_Age2+ERQ_CRc+SEX
SDQ_C_INT1 ~ YINTERNAL + ERQ_CRc + SEX + Covid_Age
Covid_Age2 ~ Covid_Age

# variances
SDQ_C_INT2 ~~ SDQ_C_INT2
SDQ_C_INT1 ~~ SDQ_C_INT1
YINTERNAL ~~ YINTERNAL
ERQ_CRc~~ERQ_CRc
SEX ~~ SEX
Covid_Age~~Covid_Age
Covid_Age2~~Covid_Age2

# covariances
YINTERNAL~~ERQ_CRc
YINTERNAL~~SEX
SEX~~ERQ_CRc
Covid_Age~~ERQ_CRc
Covid_Age2~~ERQ_CRc
Covid_Age~~SEX
Covid_Age2~~SEX
```

```
Covid_Age~~YINTERNAL
Covid_Age2~~SDQ_C_INT1

# intercepts
SDQ_C_INT2 ~1
SDQ_C_INT1 ~1
YINTERNAL ~1
ERQ_CRc~1
SEX ~1
Covid_Age~1
Covid_Age2~1
'

CRMod1.fit <- sem(CRMod1, estimator = "ML", missing ="ML", data = dt.covid)
summary(CRMod1.fit, standardized=TRUE, fit.measures = TRUE)

## lavaan 0.6-6 ended normally after 128 iterations
##
##   Estimator                                         ML
##   Optimization method                           NLMINB
##   Number of free parameters                         32
##
##   Number of observations                           145
##   Number of missing patterns                         4
##
## Model Test User Model:
##
##   Test statistic                                 3.197
##   Degrees of freedom                                 3
##   P-value (Chi-square)                           0.362
##
## Model Test Baseline Model:
##
##   Test statistic                               700.409
##   Degrees of freedom                                21
##   P-value                                        0.000
##
## User Model versus Baseline Model:
##
##   Comparative Fit Index (CFI)                    1.000
##   Tucker-Lewis Index (TLI)                       0.998
##
## Loglikelihood and Information Criteria:
##
##   Loglikelihood user model (H0)                -45.349
##   Loglikelihood unrestricted model (H1)        -43.751
##
##   Akaike (AIC)                                 154.699
##   Bayesian (BIC)                               249.954
##   Sample-size adjusted Bayesian (BIC)          148.695
```

```
##
## Root Mean Square Error of Approximation:
##
##   RMSEA                                          0.021
##   90 Percent confidence interval - lower         0.000
##   90 Percent confidence interval - upper         0.143
##   P-value RMSEA <= 0.05                          0.513
##
## Standardized Root Mean Square Residual:
##
##   SRMR                                           0.007
##
## Parameter Estimates:
##
##   Standard errors                             Standard
##   Information                                 Observed
##   Observed information based on                Hessian
##
## Regressions:
##                    Estimate  Std.Err  z-value  P(>|z|)   Std.lv  Std.all
##   SDQ_C_INT2 ~
##     SDQ_C_INT1        0.683    0.109    6.281    0.000    0.683    0.534
##     Covid_Age2       -0.055    0.041   -1.333    0.183   -0.055   -0.096
##     ERQ_CRc          -0.015    0.033   -0.457    0.647   -0.015   -0.037
##     SEX               0.083    0.043    1.956    0.050    0.083    0.156
##   SDQ_C_INT1 ~
##     YINTERNAL         0.181    0.072    2.504    0.012    0.181    0.195
##     ERQ_CRc          -0.120    0.023   -5.169    0.000   -0.120   -0.374
##     SEX               0.149    0.032    4.604    0.000    0.149    0.355
##     Covid_Age        -0.017    0.033   -0.527    0.598   -0.017   -0.038
##   Covid_Age2 ~
##     Covid_Age         1.005    0.009  113.601    0.000    1.005    0.995
##
## Covariances:
##                    Estimate  Std.Err  z-value  P(>|z|)   Std.lv  Std.all
##   ERQ_CRc ~~
##     YINTERNAL        -0.019    0.012   -1.589    0.112   -0.019   -0.131
##   SEX ~~
##     YINTERNAL        -0.041    0.010   -4.197    0.000   -0.041   -0.373
##   ERQ_CRc ~~
##     SEX               0.002    0.027    0.072    0.942    0.002    0.006
##     Covid_Age        -0.012    0.025   -0.466    0.641   -0.012   -0.039
##  .Covid_Age2 ~~
##     ERQ_CRc          -0.008    0.003   -3.013    0.003   -0.008   -0.281
##   SEX ~~
##     Covid_Age         0.032    0.019    1.664    0.096    0.032    0.140
##  .Covid_Age2 ~~
##     SEX               0.001    0.002    0.511    0.609    0.001    0.044
##   YINTERNAL ~~
##     Covid_Age        -0.015    0.009   -1.712    0.087   -0.015   -0.144
```

```
##   .SDQ_C_INT1 ~~
##     .Covid_Age2        0.001    0.001    1.825    0.068    0.001    0.171
##
## Intercepts:
##                     Estimate  Std.Err  z-value  P(>|z|)   Std.lv  Std.all
##     .SDQ_C_INT2        0.982    0.614    1.600    0.110    0.982    3.697
##     .SDQ_C_INT1        0.347    0.476    0.729    0.466    0.347    1.669
##      YINTERNAL         0.410    0.019   22.074    0.000    0.410    1.833
##      ERQ_CRc          -0.010    0.054   -0.194    0.846   -0.010   -0.016
##      SEX               0.434    0.041   10.568    0.000    0.434    0.878
##      Covid_Age        14.375    0.038  376.002    0.000   14.375   31.227
##     .Covid_Age2        0.499    0.127    3.921    0.000    0.499    1.073
##
## Variances:
##                     Estimate  Std.Err  z-value  P(>|z|)   Std.lv  Std.all
##     .SDQ_C_INT2        0.044    0.006    7.665    0.000    0.044    0.626
##     .SDQ_C_INT1        0.032    0.004    8.446    0.000    0.032    0.731
##      YINTERNAL         0.050    0.006    8.515    0.000    0.050    1.000
##      ERQ_CRc           0.419    0.050    8.416    0.000    0.419    1.000
##      SEX               0.245    0.029    8.528    0.000    0.245    1.000
##      Covid_Age         0.212    0.025    8.512    0.000    0.212    1.000
##     .Covid_Age2        0.002    0.000    7.677    0.000    0.002    0.009
```

```
#Cognitive Reappraisal, interaction with pandemic-related stress
dt.covid$CRxStress1<-dt.covid$ERQ_CRc*dt.covid$COVID_Stress1
dt.covid$CRxStress2<-dt.covid$ERQ_CRc*dt.covid$COVID_Stress2
CRMod2 <- '
# regressions
SDQ_C_INT2 ~ SDQ_C_INT1+Covid_Age2+ERQ_CRc+COVID_Stress2+CRxStress2+SEX
SDQ_C_INT1 ~ YINTERNAL + ERQ_CRc + SEX + Covid_Age+COVID_Stress1+CRxStress1
Covid_Age2 ~ Covid_Age
COVID_Stress2~COVID_Stress1
CRxStress2~CRxStress1

# variances
SDQ_C_INT2 ~~ SDQ_C_INT2
SDQ_C_INT1 ~~ SDQ_C_INT1
YINTERNAL ~~ YINTERNAL
ERQ_CRc~~ERQ_CRc
SEX ~~ SEX
Covid_Age~~Covid_Age
Covid_Age2~~Covid_Age2
COVID_Stress1~~COVID_Stress1
COVID_Stress2~~COVID_Stress2
CRxStress1~~CRxStress1
CRxStress2~~CRxStress2

# covariances
YINTERNAL~~ERQ_CRc
YINTERNAL~~SEX
```

```
SEX~~ERQ_CRc
Covid_Age~~ERQ_CRc
Covid_Age2~~ERQ_CRc
Covid_Age~~SEX
Covid_Age2~~SEX
Covid_Age~~YINTERNAL
Covid_Age2~~SDQ_C_INT1
COVID_Stress1~~YINTERNAL
COVID_Stress1~~SEX
COVID_Stress1~~ERQ_CRc
COVID_Stress1~~Covid_Age
COVID_Stress2~~SDQ_C_INT1
COVID_Stress2~~Covid_Age2
COVID_Stress2~~ERQ_CRc
COVID_Stress2~~SEX
COVID_Stress1~~CRxStress1
CRxStress1~~YINTERNAL
CRxStress1~~SEX
CRxStress1~~ERQ_CRc
CRxStress1~~Covid_Age
COVID_Stress2~~CRxStress2
CRxStress2~~SDQ_C_INT1
CRxStress2~~Covid_Age2
CRxStress2~~ERQ_CRc
CRxStress2~~SEX

# intercepts
SDQ_C_INT2 ~1
SDQ_C_INT1 ~1
YINTERNAL ~1
ERQ_CRc~1
SEX ~1
Covid_Age~1
Covid_Age2~1
COVID_Stress1~1
COVID_Stress2~1
CRxStress1~1
CRxStress2~1
'
CRMod2.fit <- sem(CRMod2, estimator = "ML", missing ="ML", data = dt.covid)
summary(CRMod2.fit, standardized=TRUE, fit.measures = TRUE)

## lavaan 0.6-6 ended normally after 168 iterations
##
##   Estimator                                         ML
##   Optimization method                           NLMINB
##   Number of free parameters                         64
##
##   Number of observations                           145
##   Number of missing patterns                         5
```

```
##
## Model Test User Model:
##
##   Test statistic                                22.796
##   Degrees of freedom                                13
##   P-value (Chi-square)                           0.044
##
## Model Test Baseline Model:
##
##   Test statistic                               897.132
##   Degrees of freedom                                55
##   P-value                                        0.000
##
## User Model versus Baseline Model:
##
##   Comparative Fit Index (CFI)                    0.988
##   Tucker-Lewis Index (TLI)                       0.951
##
## Loglikelihood and Information Criteria:
##
##   Loglikelihood user model (H0)               -959.363
##   Loglikelihood unrestricted model (H1)       -947.965
##
##   Akaike (AIC)                                2046.726
##   Bayesian (BIC)                              2237.236
##   Sample-size adjusted Bayesian (BIC)         2034.718
##
## Root Mean Square Error of Approximation:
##
##   RMSEA                                          0.072
##   90 Percent confidence interval - lower         0.012
##   90 Percent confidence interval - upper         0.120
##   P-value RMSEA <= 0.05                          0.207
##
## Standardized Root Mean Square Residual:
##
##   SRMR                                           0.052
##
## Parameter Estimates:
##
##   Standard errors                             Standard
##   Information                                 Observed
##   Observed information based on                Hessian
##
## Regressions:
##                    Estimate  Std.Err  z-value  P(>|z|)   Std.lv  Std.all
##   SDQ_C_INT2 ~
##     SDQ_C_INT1        0.568    0.113    5.038    0.000    0.568    0.442
##     Covid_Age2       -0.041    0.040   -1.012    0.312   -0.041   -0.072
##     ERQ_CRc          -0.020    0.032   -0.603    0.547   -0.020   -0.048
```

```
##     COVID_Stress2      0.036    0.012    2.973    0.003    0.036    0.229
##     CRxStress2        -0.003    0.012   -0.225    0.822   -0.003   -0.017
##     SEX                0.091    0.041    2.201    0.028    0.091    0.172
##   SDQ_C_INT1 ~
##     YINTERNAL          0.173    0.066    2.641    0.008    0.173    0.190
##     ERQ_CRc           -0.085    0.022   -3.816    0.000   -0.085   -0.268
##     SEX                0.139    0.030    4.714    0.000    0.139    0.339
##     Covid_Age         -0.005    0.030   -0.158    0.875   -0.005   -0.011
##     COVID_Stress1      0.040    0.008    4.833    0.000    0.040    0.338
##     CRxStress1        -0.011    0.011   -0.965    0.335   -0.011   -0.067
##   Covid_Age2 ~
##     Covid_Age          1.006    0.009  116.799    0.000    1.006    0.995
##   COVID_Stress2 ~
##     COVID_Stress1      0.394    0.080    4.921    0.000    0.394    0.404
##   CRxStress2 ~
##     CRxStress1         0.874    0.079   11.080    0.000    0.874    0.700
##
## Covariances:
##                     Estimate  Std.Err  z-value  P(>|z|)   Std.lv  Std.all
##   ERQ_CRc ~~
##     YINTERNAL         -0.019    0.012   -1.547    0.122   -0.019   -0.128
##   SEX ~~
##     YINTERNAL         -0.042    0.010   -4.239    0.000   -0.042   -0.378
##   ERQ_CRc ~~
##     SEX                0.004    0.027    0.140    0.889    0.004    0.012
##     Covid_Age         -0.012    0.025   -0.490    0.624   -0.012   -0.042
##  .Covid_Age2 ~~
##     ERQ_CRc           -0.006    0.003   -2.448    0.014   -0.006   -0.217
##   SEX ~~
##     Covid_Age          0.033    0.019    1.703    0.089    0.033    0.145
##  .Covid_Age2 ~~
##     SEX                0.001    0.002    0.452    0.651    0.001    0.039
##   YINTERNAL ~~
##     Covid_Age         -0.015    0.009   -1.712    0.087   -0.015   -0.144
##  .SDQ_C_INT1 ~~
##    .Covid_Age2         0.001    0.001    1.261    0.207    0.001    0.116
##   YINTERNAL ~~
##     COVID_Stress1      0.001    0.032    0.045    0.964    0.001    0.004
##   SEX ~~
##     COVID_Stress1      0.031    0.073    0.430    0.667    0.031    0.036
##   ERQ_CRc ~~
##     COVID_Stress1     -0.282    0.096   -2.934    0.003   -0.282   -0.251
##   Covid_Age ~~
##     COVID_Stress1     -0.026    0.066   -0.396    0.692   -0.026   -0.033
##  .SDQ_C_INT1 ~~
##    .COVID_Stress2      0.032    0.023    1.355    0.176    0.032    0.126
##  .Covid_Age2 ~~
##    .COVID_Stress2      0.010    0.007    1.590    0.112    0.010    0.149
##  .COVID_Stress2 ~~
##     ERQ_CRc            0.069    0.089    0.775    0.438    0.069    0.069
```

```
##     SEX                 0.042    0.066    0.628    0.530    0.042    0.054
##   COVID_Stress1 ~~
##     CRxStress1         -0.313    0.190   -1.645    0.100   -0.313   -0.139
##   YINTERNAL ~~
##     CRxStress1          0.001    0.024    0.043    0.966    0.001    0.004
##   SEX ~~
##     CRxStress1         -0.027    0.055   -0.491    0.623   -0.027   -0.042
##   ERQ_CRc ~~
##     CRxStress1          0.152    0.072    2.128    0.033    0.152    0.182
##   Covid_Age ~~
##     CRxStress1          0.038    0.050    0.748    0.455    0.038    0.063
##  .COVID_Stress2 ~~
##    .CRxStress2         -0.051    0.170   -0.301    0.763   -0.051   -0.029
##  .SDQ_C_INT1 ~~
##    .CRxStress2         -0.020    0.017   -1.165    0.244   -0.020   -0.107
##  .Covid_Age2 ~~
##    .CRxStress2         -0.013    0.005   -2.564    0.010   -0.013   -0.252
##  .CRxStress2 ~~
##     ERQ_CRc            -0.054    0.065   -0.830    0.406   -0.054   -0.073
##     SEX                -0.007    0.049   -0.151    0.880   -0.007   -0.013
##
## Intercepts:
##                     Estimate  Std.Err  z-value  P(>|z|)   Std.lv  Std.all
##    .SDQ_C_INT2          0.795    0.597    1.333    0.183    0.795    3.033
##    .SDQ_C_INT1          0.171    0.436    0.393    0.694    0.171    0.840
##     YINTERNAL           0.410    0.019   22.074    0.000    0.410    1.833
##     ERQ_CRc            -0.008    0.054   -0.144    0.886   -0.008   -0.012
##     SEX                 0.434    0.041   10.546    0.000    0.434    0.876
##     Covid_Age          14.375    0.038  375.943    0.000   14.375   31.222
##    .Covid_Age2          0.495    0.124    3.996    0.000    0.495    1.063
##     COVID_Stress1      -0.031    0.144   -0.215    0.829   -0.031   -0.018
##    .COVID_Stress2      -0.094    0.139   -0.677    0.498   -0.094   -0.056
##     CRxStress1         -0.293    0.108   -2.705    0.007   -0.293   -0.226
##    .CRxStress2          0.164    0.107    1.524    0.127    0.164    0.101
##
## Variances:
##                     Estimate  Std.Err  z-value  P(>|z|)   Std.lv  Std.all
##    .SDQ_C_INT2          0.041    0.005    7.667    0.000    0.041    0.599
##    .SDQ_C_INT1          0.026    0.003    8.416    0.000    0.026    0.627
##     YINTERNAL           0.050    0.006    8.515    0.000    0.050    1.000
##     ERQ_CRc             0.417    0.049    8.439    0.000    0.417    1.000
##     SEX                 0.246    0.029    8.477    0.000    0.246    1.000
##     Covid_Age           0.212    0.025    8.512    0.000    0.212    1.000
##    .Covid_Age2          0.002    0.000    7.685    0.000    0.002    0.009
##     COVID_Stress1       3.017    0.354    8.515    0.000    3.017    1.000
##    .COVID_Stress2       2.396    0.304    7.880    0.000    2.396    0.837
##     CRxStress1          1.681    0.199    8.459    0.000    1.681    1.000
##    .CRxStress2          1.334    0.171    7.809    0.000    1.334    0.510
```

```
p.adjust(c(.018,.842,.000,.044,.000,.335),method="fdr")
```

```
## [1] 0.036 0.842 0.000 0.066 0.000 0.402

p.adjust(c(.791,.537,.018,.946,.647,.822),method="fdr")

## [1] 0.946 0.946 0.108 0.946 0.946 0.946
```

Amygdala Activation Models

```
#Amygdala Reactivity to fear vs. neutral faces
#Left amygdala, main effects
LAmygMod1 <- '
# regressions
SDQ_C_INT2 ~ SDQ_C_INT1+Covid_Age2+LAmyg.Fear_GT_Calm_c+SEX
SDQ_C_INT1 ~ YINTERNAL + LAmyg.Fear_GT_Calm_c + SEX + Covid_Age
Covid_Age2 ~ Covid_Age

# variances
SDQ_C_INT2 ~~ SDQ_C_INT2
SDQ_C_INT1 ~~ SDQ_C_INT1
YINTERNAL ~~ YINTERNAL
LAmyg.Fear_GT_Calm_c~~LAmyg.Fear_GT_Calm_c
SEX ~~ SEX
Covid_Age~~Covid_Age
Covid_Age2~~Covid_Age2

# covariances
YINTERNAL~~LAmyg.Fear_GT_Calm_c
YINTERNAL~~SEX
SEX~~LAmyg.Fear_GT_Calm_c
Covid_Age~~LAmyg.Fear_GT_Calm_c
Covid_Age2~~LAmyg.Fear_GT_Calm_c
Covid_Age~~SEX
Covid_Age2~~SEX
Covid_Age~~YINTERNAL
Covid_Age2~~SDQ_C_INT1

# intercepts
SDQ_C_INT2 ~1
SDQ_C_INT1 ~1
YINTERNAL ~1
LAmyg.Fear_GT_Calm_c~1
SEX ~1
Covid_Age~1
Covid_Age2~1
'

LAmygMod1.fit <- sem(LAmygMod1, estimator = "ML", missing ="ML", data = dt.co
vid)
summary(LAmygMod1.fit, standardized=TRUE,fit.measures = TRUE)
```

```
## lavaan 0.6-6 ended normally after 140 iterations
##
##   Estimator                                         ML
##   Optimization method                           NLMINB
##   Number of free parameters                         32
##
##   Number of observations                           145
##   Number of missing patterns                         6
##
## Model Test User Model:
##
##   Test statistic                                 3.555
##   Degrees of freedom                                 3
##   P-value (Chi-square)                           0.314
##
## Model Test Baseline Model:
##
##   Test statistic                               681.879
##   Degrees of freedom                                21
##   P-value                                        0.000
##
## User Model versus Baseline Model:
##
##   Comparative Fit Index (CFI)                    0.999
##   Tucker-Lewis Index (TLI)                       0.994
##
## Loglikelihood and Information Criteria:
##
##   Loglikelihood user model (H0)                -73.931
##   Loglikelihood unrestricted model (H1)        -72.153
##
##   Akaike (AIC)                                 211.862
##   Bayesian (BIC)                               307.117
##   Sample-size adjusted Bayesian (BIC)          205.858
##
## Root Mean Square Error of Approximation:
##
##   RMSEA                                          0.036
##   90 Percent confidence interval - lower         0.000
##   90 Percent confidence interval - upper         0.149
##   P-value RMSEA <= 0.05                          0.464
##
## Standardized Root Mean Square Residual:
##
##   SRMR                                           0.008
##
## Parameter Estimates:
##
##   Standard errors                             Standard
##   Information                                 Observed
```

```
##   Observed information based on                Hessian
##
## Regressions:
##                      Estimate  Std.Err  z-value  P(>|z|)   Std.lv  Std.all
##   SDQ_C_INT2 ~
##     SDQ_C_INT1          0.699    0.101    6.894    0.000    0.699    0.547
##     Covid_Age2         -0.056    0.041   -1.363    0.173   -0.056   -0.098
##     LAmyg.Fr_GT_C_     -0.005    0.023   -0.215    0.830   -0.005   -0.017
##     SEX                 0.080    0.042    1.900    0.057    0.080    0.149
##   SDQ_C_INT1 ~
##     YINTERNAL           0.241    0.075    3.194    0.001    0.241    0.260
##     LAmyg.Fr_GT_C_     -0.054    0.019   -2.831    0.005   -0.054   -0.233
##     SEX                 0.149    0.034    4.318    0.000    0.149    0.354
##     Covid_Age          -0.002    0.035   -0.049    0.961   -0.002   -0.004
##   Covid_Age2 ~
##     Covid_Age           1.005    0.009  113.481    0.000    1.005    0.995
##
## Covariances:
##                             Estimate  Std.Err  z-value  P(>|z|)   Std.lv  St
d.all
##   LAmyg.Fear_GT_Calm_c ~~
##     YINTERNAL                  0.011    0.018    0.622    0.534    0.011
0.055
##   SEX ~~
##     YINTERNAL                 -0.041    0.010   -4.199    0.000   -0.041   -
0.373
##   LAmyg.Fear_GT_Calm_c ~~
##     SEX                       -0.035    0.040   -0.871    0.384   -0.035   -
0.079
##     Covid_Age                  0.009    0.037    0.250    0.803    0.009
0.023
##  .Covid_Age2 ~~
##     LAmyg.Fr_GT_C_            -0.010    0.004   -2.464    0.014   -0.010   -
0.250
##   SEX ~~
##     Covid_Age                  0.032    0.019    1.664    0.096    0.032
0.140
##  .Covid_Age2 ~~
##     SEX                        0.001    0.002    0.377    0.706    0.001
0.033
##   YINTERNAL ~~
##     Covid_Age                 -0.015    0.009   -1.707    0.088   -0.015   -
0.143
##  .SDQ_C_INT1 ~~
##    .Covid_Age2                 0.002    0.001    2.356    0.018    0.002
0.223
##
## Intercepts:
##                      Estimate  Std.Err  z-value  P(>|z|)   Std.lv  Std.all
##    .SDQ_C_INT2          0.999    0.614    1.628    0.103    0.999    3.760
```

```
##    .SDQ_C_INT1        0.099    0.506    0.196    0.845    0.099    0.477
##     YINTERNAL         0.410    0.019   22.074    0.000    0.410    1.833
##     LAmyg.Fr_GT_C_    0.009    0.080    0.118    0.906    0.009    0.011
##     SEX               0.434    0.041   10.565    0.000    0.434    0.877
##     Covid_Age        14.376    0.038  376.277    0.000   14.376   31.250
##    .Covid_Age2        0.505    0.127    3.969    0.000    0.505    1.088
##
## Variances:
##                    Estimate  Std.Err  z-value  P(>|z|)   Std.lv  Std.all
##    .SDQ_C_INT2        0.044    0.006    7.664    0.000    0.044    0.627
##    .SDQ_C_INT1        0.035    0.004    8.358    0.000    0.035    0.815
##     YINTERNAL         0.050    0.006    8.515    0.000    0.050    1.000
##     LAmyg.Fr_GT_C_    0.795    0.102    7.829    0.000    0.795    1.000
##     SEX               0.245    0.029    8.522    0.000    0.245    1.000
##     Covid_Age         0.212    0.025    8.512    0.000    0.212    1.000
##    .Covid_Age2        0.002    0.000    7.640    0.000    0.002    0.009
```

```
#Left amygdala, interaction with pandemic-related stress
dt.covid$LAmygxStress1<-dt.covid$LAmyg.Fear_GT_Calm_c*dt.covid$COVID_Stress1
dt.covid$LAmygxStress2<-dt.covid$LAmyg.Fear_GT_Calm_c*dt.covid$COVID_Stress2
LAmygMod2 <- '
# regressions
SDQ_C_INT2 ~ SDQ_C_INT1+Covid_Age2+LAmyg.Fear_GT_Calm_c+COVID_Stress2+LAmygxS
tress2+SEX
SDQ_C_INT1 ~ YINTERNAL + LAmyg.Fear_GT_Calm_c + SEX + Covid_Age+COVID_Stress1
+LAmygxStress1
Covid_Age2 ~ Covid_Age
COVID_Stress2~COVID_Stress1
LAmygxStress2~LAmygxStress1

# variances
SDQ_C_INT2 ~~ SDQ_C_INT2
SDQ_C_INT1 ~~ SDQ_C_INT1
YINTERNAL ~~ YINTERNAL
LAmyg.Fear_GT_Calm_c~~LAmyg.Fear_GT_Calm_c
SEX ~~ SEX
Covid_Age~~Covid_Age
Covid_Age2~~Covid_Age2
COVID_Stress1~~COVID_Stress1
COVID_Stress2~~COVID_Stress2
LAmygxStress1~~LAmygxStress1
LAmygxStress2~~LAmygxStress2

# covariances
YINTERNAL~~LAmyg.Fear_GT_Calm_c
YINTERNAL~~SEX
SEX~~LAmyg.Fear_GT_Calm_c
Covid_Age~~LAmyg.Fear_GT_Calm_c
Covid_Age2~~LAmyg.Fear_GT_Calm_c
Covid_Age~~SEX
```

```
Covid_Age2~~SEX
Covid_Age~~YINTERNAL
Covid_Age2~~SDQ_C_INT1
COVID_Stress1~~YINTERNAL
COVID_Stress1~~SEX
COVID_Stress1~~LAmyg.Fear_GT_Calm_c
COVID_Stress1~~Covid_Age
COVID_Stress2~~SDQ_C_INT1
COVID_Stress2~~Covid_Age2
COVID_Stress2~~LAmyg.Fear_GT_Calm_c
COVID_Stress2~~SEX
LAmygxStress1~~COVID_Stress1
LAmygxStress1~~YINTERNAL
LAmygxStress1~~SEX
LAmygxStress1~~LAmyg.Fear_GT_Calm_c
LAmygxStress1~~Covid_Age
LAmygxStress2~~COVID_Stress2
LAmygxStress2~~SDQ_C_INT1
LAmygxStress2~~Covid_Age2
LAmygxStress2~~LAmyg.Fear_GT_Calm_c
LAmygxStress2~~SEX
# intercepts
SDQ_C_INT2 ~1
SDQ_C_INT1 ~1
YINTERNAL ~1
LAmyg.Fear_GT_Calm_c~1
SEX ~1
Covid_Age~1
Covid_Age2~1
COVID_Stress1~1
COVID_Stress2~1
LAmygxStress1~1
LAmygxStress2~1
'
LAmygMod2.fit <- sem(LAmygMod2, estimator = "ML", missing ="ML", data = dt.co
vid)
summary(LAmygMod2.fit, , standardized=TRUE, fit.measures = TRUE)

## lavaan 0.6-6 ended normally after 165 iterations
##
##   Estimator                                         ML
##   Optimization method                           NLMINB
##   Number of free parameters                         64
##
##   Number of observations                           145
##   Number of missing patterns                         7
##
## Model Test User Model:
##
##   Test statistic                                11.949
```

```
##   Degrees of freedom                                13
##   P-value (Chi-square)                           0.532
##
## Model Test Baseline Model:
##
##   Test statistic                               804.478
##   Degrees of freedom                                55
##   P-value                                        0.000
##
## User Model versus Baseline Model:
##
##   Comparative Fit Index (CFI)                    1.000
##   Tucker-Lewis Index (TLI)                       1.006
##
## Loglikelihood and Information Criteria:
##
##   Loglikelihood user model (H0)               -968.592
##   Loglikelihood unrestricted model (H1)       -962.618
##
##   Akaike (AIC)                                2065.184
##   Bayesian (BIC)                              2255.695
##   Sample-size adjusted Bayesian (BIC)         2053.177
##
## Root Mean Square Error of Approximation:
##
##   RMSEA                                          0.000
##   90 Percent confidence interval - lower         0.000
##   90 Percent confidence interval - upper         0.077
##   P-value RMSEA <= 0.05                          0.801
##
## Standardized Root Mean Square Residual:
##
##   SRMR                                           0.029
##
## Parameter Estimates:
##
##   Standard errors                             Standard
##   Information                                 Observed
##   Observed information based on                Hessian
##
## Regressions:
##                    Estimate  Std.Err  z-value  P(>|z|)   Std.lv  Std.all
##   SDQ_C_INT2 ~
##     SDQ_C_INT1        0.598    0.106    5.613    0.000    0.598    0.464
##     Covid_Age2       -0.039    0.040   -0.975    0.329   -0.039   -0.069
##     LAmyg.Fr_GT_C_   -0.001    0.023   -0.030    0.976   -0.001   -0.002
##     COVID_Stress2     0.036    0.012    3.042    0.002    0.036    0.229
##     LAmygxStress2    -0.001    0.013   -0.076    0.939   -0.001   -0.006
##     SEX               0.086    0.041    2.125    0.034    0.086    0.163
##   SDQ_C_INT1 ~
```

```
##     YINTERNAL         0.191    0.067    2.846    0.004    0.191    0.209
##     LAmyg.Fr_GT_C_   -0.031    0.018   -1.741    0.082   -0.031   -0.132
##     SEX               0.133    0.030    4.375    0.000    0.133    0.323
##     Covid_Age         0.012    0.031    0.394    0.694    0.012    0.027
##     COVID_Stress1     0.042    0.008    5.076    0.000    0.042    0.360
##     LAmygxStress1    -0.028    0.010   -2.734    0.006   -0.028   -0.200
##   Covid_Age2 ~
##     Covid_Age         1.008    0.008  119.088    0.000    1.008    0.995
##   COVID_Stress2 ~
##     COVID_Stress1     0.393    0.079    4.951    0.000    0.393    0.403
##   LAmygxStress2 ~
##     LAmygxStress1     0.372    0.094    3.953    0.000    0.372    0.335
##
## Covariances:
##                            Estimate  Std.Err  z-value  P(>|z|)   Std.lv  St
d.all
##   LAmyg.Fear_GT_Calm_c ~~
##     YINTERNAL                 0.015    0.017    0.841    0.400    0.015
0.073
##   SEX ~~
##     YINTERNAL                -0.042    0.010   -4.256    0.000   -0.042   -
0.380
##   LAmyg.Fear_GT_Calm_c ~~
##     SEX                      -0.037    0.040   -0.933    0.351   -0.037   -
0.084
##     Covid_Age                 0.007    0.036    0.205    0.837    0.007
0.018
##  .Covid_Age2 ~~
##     LAmyg.Fr_GT_C_           -0.008    0.004   -2.074    0.038   -0.008   -
0.202
##   SEX ~~
##     Covid_Age                 0.034    0.019    1.755    0.079    0.034
0.148
##  .Covid_Age2 ~~
##     SEX                       0.001    0.002    0.305    0.761    0.001
0.026
##   YINTERNAL ~~
##     Covid_Age                -0.015    0.009   -1.709    0.087   -0.015   -
0.143
##  .SDQ_C_INT1 ~~
##    .Covid_Age2                0.001    0.001    1.807    0.071    0.001
0.167
##   YINTERNAL ~~
##     COVID_Stress1             0.001    0.032    0.045    0.964    0.001
0.004
##   SEX ~~
##     COVID_Stress1             0.032    0.072    0.444    0.657    0.032
0.037
##   LAmyg.Fear_GT_Calm_c ~~
##     COVID_Stress1            -0.374    0.136   -2.757    0.006   -0.374   -
```

```
0.243
##   Covid_Age ~~
##     COVID_Stress1          -0.027    0.066   -0.403    0.687   -0.027   -
0.033
##   .SDQ_C_INT1 ~~
##    .COVID_Stress2           0.034    0.023    1.474    0.140    0.034
0.132
##   .Covid_Age2 ~~
##    .COVID_Stress2           0.010    0.006    1.470    0.141    0.010
0.138
##   .COVID_Stress2 ~~
##     LAmyg.Fr_GT_C_         -0.056    0.124   -0.456    0.648   -0.056   -
0.041
##     SEX                     0.054    0.064    0.839    0.401    0.054
0.070
##   COVID_Stress1 ~~
##     LAmygxStress1          -0.337    0.226   -1.490    0.136   -0.337   -
0.133
##   YINTERNAL ~~
##     LAmygxStress1          -0.036    0.029   -1.221    0.222   -0.036   -
0.110
##   SEX ~~
##     LAmygxStress1          -0.032    0.066   -0.488    0.625   -0.032   -
0.044
##   LAmyg.Fear_GT_Calm_c ~~
##     LAmygxStress1           0.006    0.115    0.049    0.961    0.006
0.004
##   Covid_Age ~~
##     LAmygxStress1           0.051    0.061    0.836    0.403    0.051
0.075
##   .COVID_Stress2 ~~
##    .LAmygxStress2          -0.128    0.222   -0.580    0.562   -0.128   -
0.054
##   .SDQ_C_INT1 ~~
##    .LAmygxStress2          -0.034    0.023   -1.502    0.133   -0.034   -
0.135
##   .Covid_Age2 ~~
##    .LAmygxStress2          -0.021    0.007   -2.997    0.003   -0.021   -
0.309
##   .LAmygxStress2 ~~
##     LAmyg.Fr_GT_C_          0.345    0.129    2.673    0.008    0.345
0.253
##     SEX                     0.009    0.066    0.129    0.897    0.009
0.011
##
## Intercepts:
##                    Estimate  Std.Err  z-value  P(>|z|)   Std.lv  Std.all
##    .SDQ_C_INT2        0.768    0.597    1.287    0.198    0.768    2.911
##    .SDQ_C_INT1       -0.082    0.443   -0.185    0.853   -0.082   -0.399
##     YINTERNAL         0.410    0.019   22.074    0.000    0.410    1.833
```

```
##     LAmyg.Fr_GT_C_    0.018    0.080    0.219    0.826    0.018    0.020
##     SEX               0.434    0.041   10.526    0.000    0.434    0.874
##     Covid_Age        14.376    0.038  376.188    0.000   14.376   31.243
##    .Covid_Age2        0.467    0.122    3.835    0.000    0.467    1.002
##     COVID_Stress1    -0.031    0.144   -0.215    0.829   -0.031   -0.018
##    .COVID_Stress2    -0.085    0.139   -0.609    0.542   -0.085   -0.050
##     LAmygxStress1    -0.395    0.132   -2.995    0.003   -0.395   -0.270
##    .LAmygxStress2    -0.062    0.151   -0.413    0.680   -0.062   -0.038
##
## Variances:
##                    Estimate  Std.Err  z-value  P(>|z|)   Std.lv  Std.all
##    .SDQ_C_INT2        0.041    0.005    7.671    0.000    0.041    0.594
##    .SDQ_C_INT1        0.027    0.003    8.328    0.000    0.027    0.647
##     YINTERNAL         0.050    0.006    8.515    0.000    0.050    1.000
##     LAmyg.Fr_GT_C_    0.789    0.100    7.917    0.000    0.789    1.000
##     SEX               0.247    0.029    8.449    0.000    0.247    1.000
##     Covid_Age         0.212    0.025    8.513    0.000    0.212    1.000
##    .Covid_Age2        0.002    0.000    7.734    0.000    0.002    0.009
##     COVID_Stress1     3.017    0.354    8.515    0.000    3.017    1.000
##    .COVID_Stress2     2.393    0.303    7.889    0.000    2.393    0.837
##     LAmygxStress1     2.142    0.274    7.817    0.000    2.142    1.000
##    .LAmygxStress2     2.350    0.321    7.327    0.000    2.350    0.888
```

```
#Right amygdala, main effects
RAmygMod1 <- '
# regressions
SDQ_C_INT2 ~ SDQ_C_INT1+Covid_Age2+RAmyg.Fear_GT_Calm_c+SEX
SDQ_C_INT1 ~ YINTERNAL + RAmyg.Fear_GT_Calm_c + SEX + Covid_Age
Covid_Age2 ~ Covid_Age

# variances
SDQ_C_INT2 ~~ SDQ_C_INT2
SDQ_C_INT1 ~~ SDQ_C_INT1
YINTERNAL ~~ YINTERNAL
RAmyg.Fear_GT_Calm_c~~RAmyg.Fear_GT_Calm_c
SEX ~~ SEX
Covid_Age~~Covid_Age
Covid_Age2~~Covid_Age2

# covariances
YINTERNAL~~RAmyg.Fear_GT_Calm_c
YINTERNAL~~SEX
SEX~~RAmyg.Fear_GT_Calm_c
Covid_Age~~RAmyg.Fear_GT_Calm_c
Covid_Age2~~RAmyg.Fear_GT_Calm_c
Covid_Age~~SEX
Covid_Age2~~SEX
Covid_Age~~YINTERNAL
Covid_Age2~~SDQ_C_INT1
```

```
# intercepts
SDQ_C_INT2 ~1
SDQ_C_INT1 ~1
YINTERNAL ~1
RAmyg.Fear_GT_Calm_c~1
SEX ~1
Covid_Age~1
Covid_Age2~1
'

RAmygMod1.fit <- sem(RAmygMod1, estimator = "ML", missing ="ML", data = dt.co
vid)
summary(RAmygMod1.fit, standardized=TRUE, fit.measures = TRUE)
```

```
## lavaan 0.6-6 ended normally after 129 iterations
##
##   Estimator                                         ML
##   Optimization method                           NLMINB
##   Number of free parameters                         32
##
##   Number of observations                           145
##   Number of missing patterns                         6
##
## Model Test User Model:
##
##   Test statistic                                 3.702
##   Degrees of freedom                                 3
##   P-value (Chi-square)                           0.296
##
## Model Test Baseline Model:
##
##   Test statistic                               670.828
##   Degrees of freedom                                21
##   P-value                                        0.000
##
## User Model versus Baseline Model:
##
##   Comparative Fit Index (CFI)                    0.999
##   Tucker-Lewis Index (TLI)                       0.992
##
## Loglikelihood and Information Criteria:
##
##   Loglikelihood user model (H0)                -75.356
##   Loglikelihood unrestricted model (H1)        -73.505
##
##   Akaike (AIC)                                 214.713
##   Bayesian (BIC)                               309.968
##   Sample-size adjusted Bayesian (BIC)          208.709
##
## Root Mean Square Error of Approximation:
```

```
##
##   RMSEA                                          0.040
##   90 Percent confidence interval - lower         0.000
##   90 Percent confidence interval - upper         0.151
##   P-value RMSEA <= 0.05                          0.445
##
## Standardized Root Mean Square Residual:
##
##   SRMR                                           0.006
##
## Parameter Estimates:
##
##   Standard errors                             Standard
##   Information                                 Observed
##   Observed information based on                Hessian
##
## Regressions:
##                      Estimate  Std.Err  z-value  P(>|z|)   Std.lv  Std.all
##   SDQ_C_INT2 ~
##     SDQ_C_INT1          0.704    0.099    7.092    0.000    0.704    0.551
##     Covid_Age2         -0.055    0.042   -1.333    0.182   -0.055   -0.097
##     RAmyg.Fr_GT_C_     -0.006    0.024   -0.239    0.811   -0.006   -0.019
##     SEX                 0.081    0.042    1.917    0.055    0.081    0.150
##   SDQ_C_INT1 ~
##     YINTERNAL           0.239    0.077    3.109    0.002    0.239    0.258
##     RAmyg.Fr_GT_C_     -0.008    0.021   -0.394    0.694   -0.008   -0.034
##     SEX                 0.157    0.035    4.443    0.000    0.157    0.374
##     Covid_Age          -0.009    0.036   -0.243    0.808   -0.009   -0.019
##   Covid_Age2 ~
##     Covid_Age           1.005    0.009  113.159    0.000    1.005    0.995
##
## Covariances:
##                             Estimate  Std.Err  z-value  P(>|z|)   Std.lv  St
d.all
##   RAmyg.Fear_GT_Calm_c ~~
##     YINTERNAL                  0.014    0.017    0.821    0.412    0.014
0.074
##   SEX ~~
##     YINTERNAL                 -0.041    0.010   -4.202    0.000   -0.041   -
0.374
##   RAmyg.Fear_GT_Calm_c ~~
##     SEX                        0.004    0.039    0.104    0.917    0.004
0.009
##     Covid_Age                 -0.027    0.036   -0.741    0.459   -0.027   -
0.067
##  .Covid_Age2 ~~
##     RAmyg.Fr_GT_C_             0.001    0.004    0.308    0.758    0.001
0.031
##   SEX ~~
##     Covid_Age                  0.032    0.019    1.663    0.096    0.032
```

```
0.139
##   .Covid_Age2 ~~
##     SEX                      0.001    0.002    0.334    0.739    0.001
0.029
##   YINTERNAL ~~
##     Covid_Age               -0.015    0.009   -1.712    0.087   -0.015   -
0.144
##   .SDQ_C_INT1 ~~
##    .Covid_Age2               0.002    0.001    2.810    0.005    0.002
0.276
##
## Intercepts:
##                    Estimate  Std.Err  z-value  P(>|z|)   Std.lv  Std.all
##    .SDQ_C_INT2        0.984    0.618    1.594    0.111    0.984    3.703
##    .SDQ_C_INT1        0.196    0.517    0.380    0.704    0.196    0.945
##     YINTERNAL         0.410    0.019   22.074    0.000    0.410    1.833
##     RAmyg.Fr_GT_C_   -0.039    0.078   -0.498    0.618   -0.039   -0.045
##     SEX               0.434    0.041   10.564    0.000    0.434    0.877
##     Covid_Age        14.375    0.038  375.980    0.000   14.375   31.226
##    .Covid_Age2        0.507    0.128    3.969    0.000    0.507    1.090
##
## Variances:
##                    Estimate  Std.Err  z-value  P(>|z|)   Std.lv  Std.all
##    .SDQ_C_INT2        0.044    0.006    7.659    0.000    0.044    0.626
##    .SDQ_C_INT1        0.037    0.004    8.437    0.000    0.037    0.867
##     YINTERNAL         0.050    0.006    8.515    0.000    0.050    1.000
##     RAmyg.Fr_GT_C_    0.745    0.095    7.814    0.000    0.745    1.000
##     SEX               0.245    0.029    8.520    0.000    0.245    1.000
##     Covid_Age         0.212    0.025    8.512    0.000    0.212    1.000
##    .Covid_Age2        0.002    0.000    7.656    0.000    0.002    0.009

#Right amygdala, interaction with pandemic-related stress
dt.covid$RAmygxStress1<-dt.covid$RAmyg.Fear_GT_Calm_c*dt.covid$COVID_Stress1
dt.covid$RAmygxStress2<-dt.covid$RAmyg.Fear_GT_Calm_c*dt.covid$COVID_Stress2
RAmygMod2 <- '
# regressions
SDQ_C_INT2 ~ SDQ_C_INT1+Covid_Age2+RAmyg.Fear_GT_Calm_c+COVID_Stress2+RAmygxS
tress2+SEX
SDQ_C_INT1 ~ YINTERNAL + RAmyg.Fear_GT_Calm_c + SEX + Covid_Age+COVID_Stress1
+RAmygxStress1
Covid_Age2 ~ Covid_Age
COVID_Stress2~COVID_Stress1
RAmygxStress2~RAmygxStress1

# variances
SDQ_C_INT2 ~~ SDQ_C_INT2
SDQ_C_INT1 ~~ SDQ_C_INT1
YINTERNAL ~~ YINTERNAL
RAmyg.Fear_GT_Calm_c~~RAmyg.Fear_GT_Calm_c
SEX ~~ SEX
```

```
Covid_Age~~Covid_Age
Covid_Age2~~Covid_Age2
COVID_Stress1~~COVID_Stress1
COVID_Stress2~~COVID_Stress2
RAmygxStress1~~RAmygxStress1
RAmygxStress2~~RAmygxStress2

# covariances
YINTERNAL~~RAmyg.Fear_GT_Calm_c
YINTERNAL~~SEX
SEX~~RAmyg.Fear_GT_Calm_c
Covid_Age~~RAmyg.Fear_GT_Calm_c
Covid_Age2~~RAmyg.Fear_GT_Calm_c
Covid_Age~~SEX
Covid_Age2~~SEX
Covid_Age~~YINTERNAL
Covid_Age2~~SDQ_C_INT1
COVID_Stress1~~YINTERNAL
COVID_Stress1~~SEX
COVID_Stress1~~RAmyg.Fear_GT_Calm_c
COVID_Stress1~~Covid_Age
COVID_Stress2~~SDQ_C_INT1
COVID_Stress2~~Covid_Age2
COVID_Stress2~~RAmyg.Fear_GT_Calm_c
COVID_Stress2~~SEX
RAmygxStress1~~COVID_Stress1
RAmygxStress1~~YINTERNAL
RAmygxStress1~~SEX
RAmygxStress1~~RAmyg.Fear_GT_Calm_c
RAmygxStress1~~Covid_Age
RAmygxStress2~~COVID_Stress2
RAmygxStress2~~SDQ_C_INT1
RAmygxStress2~~Covid_Age2
RAmygxStress2~~RAmyg.Fear_GT_Calm_c
RAmygxStress2~~SEX
# intercepts
SDQ_C_INT2 ~1
SDQ_C_INT1 ~1
YINTERNAL ~1
RAmyg.Fear_GT_Calm_c~1
SEX ~1
Covid_Age~1
Covid_Age2~1
COVID_Stress1~1
COVID_Stress2~1
RAmygxStress1~1
RAmygxStress2~1
'
RAmygMod2.fit <- sem(RAmygMod2, estimator = "ML", missing ="ML", data = dt.co
```

```
vid)
summary(RAmygMod2.fit, standardized=TRUE, fit.measures = TRUE)
```

```
## lavaan 0.6-6 ended normally after 174 iterations
##
##   Estimator                                         ML
##   Optimization method                           NLMINB
##   Number of free parameters                         64
##
##   Number of observations                           145
##   Number of missing patterns                         7
##
## Model Test User Model:
##
##   Test statistic                                17.661
##   Degrees of freedom                                13
##   P-value (Chi-square)                           0.171
##
## Model Test Baseline Model:
##
##   Test statistic                               820.021
##   Degrees of freedom                                55
##   P-value                                        0.000
##
## User Model versus Baseline Model:
##
##   Comparative Fit Index (CFI)                    0.994
##   Tucker-Lewis Index (TLI)                       0.974
##
## Loglikelihood and Information Criteria:
##
##   Loglikelihood user model (H0)               -963.155
##   Loglikelihood unrestricted model (H1)       -954.324
##
##   Akaike (AIC)                                2054.309
##   Bayesian (BIC)                              2244.820
##   Sample-size adjusted Bayesian (BIC)         2042.302
##
## Root Mean Square Error of Approximation:
##
##   RMSEA                                          0.050
##   90 Percent confidence interval - lower         0.000
##   90 Percent confidence interval - upper         0.103
##   P-value RMSEA <= 0.05                          0.455
##
## Standardized Root Mean Square Residual:
##
##   SRMR                                           0.041
##
## Parameter Estimates:
```

```
##
##   Standard errors                                Standard
##   Information                                    Observed
##   Observed information based on                   Hessian
##
## Regressions:
##                    Estimate  Std.Err  z-value  P(>|z|)   Std.lv  Std.all
##   SDQ_C_INT2 ~
##     SDQ_C_INT1        0.594    0.102    5.845    0.000    0.594    0.461
##     Covid_Age2       -0.036    0.040   -0.883    0.377   -0.036   -0.063
##     RAmyg.Fr_GT_C_    0.006    0.023    0.257    0.797    0.006    0.020
##     COVID_Stress2     0.039    0.012    3.193    0.001    0.039    0.253
##     RAmygxStress2     0.014    0.013    1.080    0.280    0.014    0.083
##     SEX               0.084    0.040    2.086    0.037    0.084    0.159
##   SDQ_C_INT1 ~
##     YINTERNAL         0.207    0.069    3.002    0.003    0.207    0.227
##     RAmyg.Fr_GT_C_    0.019    0.019    0.989    0.323    0.019    0.081
##     SEX               0.143    0.031    4.537    0.000    0.143    0.348
##     Covid_Age         0.010    0.032    0.301    0.764    0.010    0.022
##     COVID_Stress1     0.050    0.009    5.494    0.000    0.050    0.422
##     RAmygxStress1    -0.003    0.010   -0.267    0.789   -0.003   -0.022
##   Covid_Age2 ~
##     Covid_Age         1.008    0.009  114.188    0.000    1.008    0.995
##   COVID_Stress2 ~
##     COVID_Stress1     0.434    0.077    5.655    0.000    0.434    0.438
##   RAmygxStress2 ~
##     RAmygxStress1     0.473    0.075    6.317    0.000    0.473    0.495
##
## Covariances:
##                           Estimate  Std.Err  z-value  P(>|z|)   Std.lv  St
d.all
##   RAmyg.Fear_GT_Calm_c ~~
##     YINTERNAL                0.018    0.017    1.028    0.304    0.018
0.093
##   SEX ~~
##     YINTERNAL               -0.042    0.010   -4.263    0.000   -0.042   -
0.379
##   RAmyg.Fear_GT_Calm_c ~~
##     SEX                      0.003    0.039    0.067    0.946    0.003
0.006
##     Covid_Age               -0.029    0.036   -0.810    0.418   -0.029   -
0.073
##  .Covid_Age2 ~~
##     RAmyg.Fr_GT_C_           0.002    0.004    0.627    0.530    0.002
0.059
##   SEX ~~
##     Covid_Age                0.035    0.019    1.837    0.066    0.035
0.154
##  .Covid_Age2 ~~
##     SEX                      0.001    0.002    0.271    0.786    0.001
```

```
0.024
##   YINTERNAL ~~
##     Covid_Age               -0.015    0.009   -1.711    0.087   -0.015   -
0.144
##  .SDQ_C_INT1 ~~
##    .Covid_Age2               0.002    0.001    2.180    0.029    0.002
0.210
##   YINTERNAL ~~
##     COVID_Stress1            0.001    0.032    0.045    0.964    0.001
0.004
##   SEX ~~
##     COVID_Stress1            0.023    0.073    0.322    0.748    0.023
0.027
##   RAmyg.Fear_GT_Calm_c ~~
##     COVID_Stress1           -0.411    0.139   -2.971    0.003   -0.411   -
0.274
##   Covid_Age ~~
##     COVID_Stress1           -0.026    0.066   -0.392    0.695   -0.026   -
0.033
##  .SDQ_C_INT1 ~~
##    .COVID_Stress2            0.037    0.024    1.548    0.122    0.037
0.140
##  .Covid_Age2 ~~
##    .COVID_Stress2            0.009    0.007    1.381    0.167    0.009
0.130
##  .COVID_Stress2 ~~
##     RAmyg.Fr_GT_C_          -0.057    0.118   -0.482    0.630   -0.057   -
0.043
##     SEX                      0.063    0.065    0.976    0.329    0.063
0.082
##   COVID_Stress1 ~~
##     RAmygxStress1           -0.580    0.251   -2.306    0.021   -0.580   -
0.208
##   YINTERNAL ~~
##     RAmygxStress1            0.006    0.032    0.173    0.863    0.006
0.015
##   SEX ~~
##     RAmygxStress1           -0.100    0.074   -1.350    0.177   -0.100   -
0.125
##   RAmyg.Fear_GT_Calm_c ~~
##     RAmygxStress1           -0.181    0.128   -1.415    0.157   -0.181   -
0.130
##   Covid_Age ~~
##     RAmygxStress1           -0.025    0.067   -0.376    0.707   -0.025   -
0.034
##  .COVID_Stress2 ~~
##    .RAmygxStress2           -0.629    0.205   -3.073    0.002   -0.629   -
0.305
##  .SDQ_C_INT1 ~~
##    .RAmygxStress2            0.012    0.022    0.563    0.574    0.012
```

```
0.053
##   .Covid_Age2 ~~
##     .RAmygxStress2            0.004    0.006    0.665    0.506    0.004
0.071
##   .RAmygxStress2 ~~
##      RAmyg.Fr_GT_C_           0.094    0.105    0.899    0.369    0.094
0.082
##      SEX                      0.059    0.059    1.000    0.317    0.059
0.089
##
## Intercepts:
##                    Estimate  Std.Err  z-value  P(>|z|)   Std.lv  Std.all
##    .SDQ_C_INT2        0.721    0.599    1.203    0.229    0.721    2.736
##    .SDQ_C_INT1       -0.046    0.459   -0.100    0.921   -0.046   -0.223
##     YINTERNAL         0.410    0.019   22.074    0.000    0.410    1.833
##     RAmyg.Fr_GT_C_   -0.024    0.078   -0.305    0.760   -0.024   -0.028
##     SEX               0.434    0.041   10.512    0.000    0.434    0.873
##     Covid_Age        14.375    0.038  375.903    0.000   14.375   31.219
##    .Covid_Age2        0.461    0.127    3.635    0.000    0.461    0.989
##     COVID_Stress1    -0.031    0.144   -0.215    0.829   -0.031   -0.018
##    .COVID_Stress2    -0.087    0.139   -0.624    0.533   -0.087   -0.050
##     RAmygxStress1    -0.382    0.145   -2.629    0.009   -0.382   -0.238
##    .RAmygxStress2    -0.041    0.131   -0.315    0.752   -0.041   -0.027
##
## Variances:
##                    Estimate  Std.Err  z-value  P(>|z|)   Std.lv  Std.all
##    .SDQ_C_INT2        0.041    0.005    7.669    0.000    0.041    0.586
##    .SDQ_C_INT1        0.029    0.004    8.379    0.000    0.029    0.702
##     YINTERNAL         0.050    0.006    8.515    0.000    0.050    1.000
##     RAmyg.Fr_GT_C_    0.749    0.097    7.764    0.000    0.749    1.000
##     SEX               0.248    0.029    8.423    0.000    0.248    1.000
##     Covid_Age         0.212    0.025    8.512    0.000    0.212    1.000
##    .Covid_Age2        0.002    0.000    7.677    0.000    0.002    0.009
##     COVID_Stress1     3.017    0.354    8.515    0.000    3.017    1.000
##    .COVID_Stress2     2.395    0.304    7.883    0.000    2.395    0.808
##     RAmygxStress1     2.581    0.329    7.847    0.000    2.581    1.000
##    .RAmygxStress2     1.778    0.242    7.336    0.000    1.778    0.755

#FDR correction on analyses of amygdala reactivity and internalizing at T1
p.adjust(c(.005,.694,.006,.802),method="fdr")

## [1] 0.012 0.802 0.012 0.802

#FDR correction on analyses of amygdala reactivity and internalizing at T2
p.adjust(c(.830,.811,.939,.280),method="fdr")

## [1] 0.939 0.939 0.939 0.939

# Figure 3
laaplot <- ggplot(dt.covid, aes(x = LAmyg.Fear_GT_Calm, y = SDQ_C_INT))
laaplot1 <- laaplot + geom_jitter(height = 0.3, width = 0, col = 1) + theme_c
```

```
lassic() +
    xlab("Left Amygdala Activation") + ylab("Internalizing Problems") + geom_
smooth(method = "lm",
    col = 1) + theme(axis.title = element_text(size = 25), axis.text = elemen
t_text(size = 20))
laaplot2 <- laaplot + theme_classic() + xlab("Left Amygdala Activation") + yl
ab("Internalizing Problems") +
    geom_jitter(height = 0.3, width = 0, aes(x = LAmyg.Fear_GT_Scramble, y =
SDQ_C_INT),
        col = 5) + geom_smooth(method = "lm", aes(x = LAmyg.Fear_GT_Scramble,
y = SDQ_C_INT),
    col = 5) + geom_jitter(height = 0.3, width = 0, aes(x = LAmyg.Calm_GT_Scr
amble,
    y = SDQ_C_INT), col = 6) + geom_smooth(method = "lm", aes(x = LAmyg.Calm_
GT_Scramble,
    y = SDQ_C_INT), col = 6) + theme(axis.title = element_text(size = 25), ax
is.text = element_text(size = 20))
laaplot1 + laaplot2
```

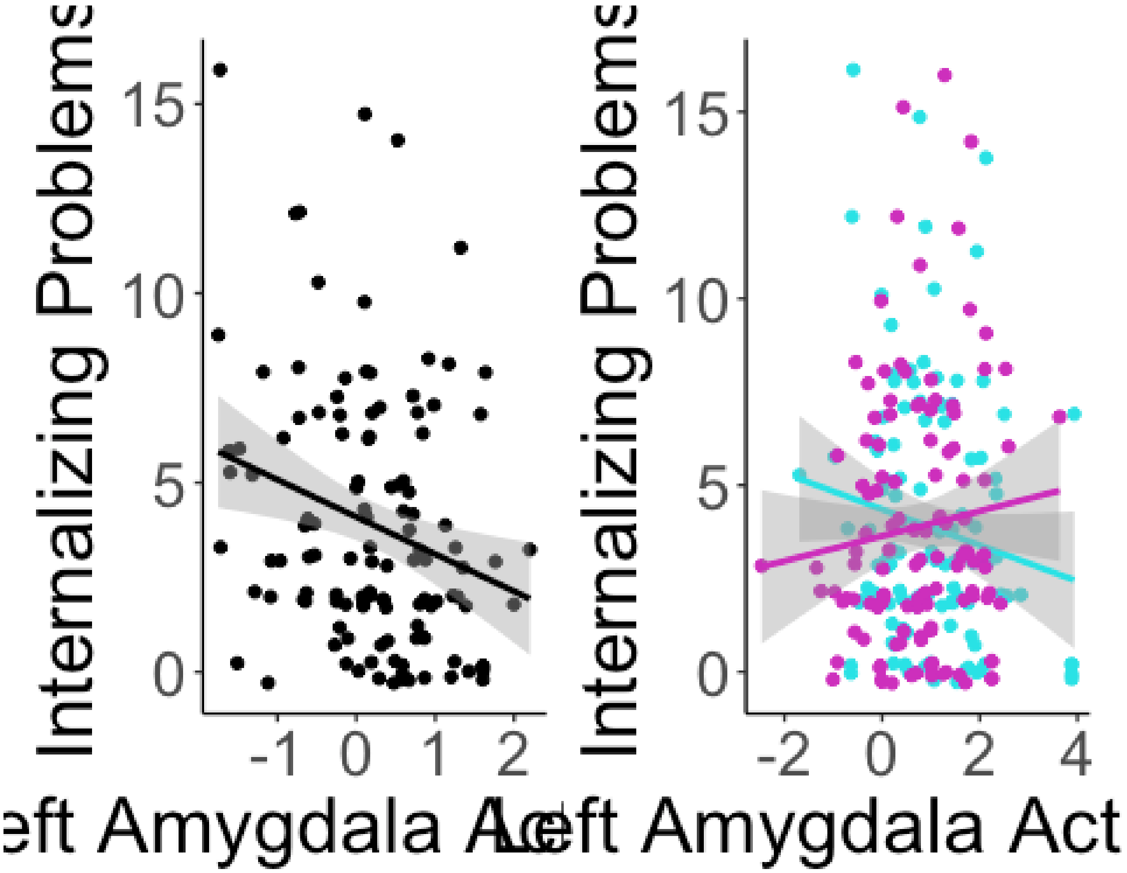

#Hippocampal and amygdala volume models

```
#Hippocampal and amygdala volume
dt.covid$IntraCranialVol<-dt.covid$IntraCranialVol/1000000
dt.covid$Hippocampus_c<-dt.covid$Hippocampus_c/1000
```

```
dt.covid$Amygdala_c<-dt.covid$Amygdala_c/1000
#Hippocampus, main effects
HippocampusMod1 <- '
# regressions
SDQ_C_INT2 ~ SDQ_C_INT1+Covid_Age2+Hippocampus_c+IntraCranialVol+SEX
SDQ_C_INT1 ~ YINTERNAL + Hippocampus_c + SEX + Covid_Age+IntraCranialVol
Covid_Age2 ~ Covid_Age

# variances
SDQ_C_INT2 ~~ SDQ_C_INT2
SDQ_C_INT1 ~~ SDQ_C_INT1
YINTERNAL ~~ YINTERNAL
Hippocampus_c~~Hippocampus_c
SEX ~~ SEX
Covid_Age~~Covid_Age
Covid_Age2~~Covid_Age2
IntraCranialVol~~IntraCranialVol

# covariances
YINTERNAL~~Hippocampus_c
YINTERNAL~~SEX
SEX~~Hippocampus_c
Covid_Age~~Hippocampus_c
Covid_Age2~~Hippocampus_c
Covid_Age~~SEX
Covid_Age2~~SEX
Covid_Age~~YINTERNAL
Covid_Age2~~SDQ_C_INT1
IntraCranialVol~~Hippocampus_c
IntraCranialVol~~Covid_Age
IntraCranialVol~~Covid_Age2
IntraCranialVol~~SEX
IntraCranialVol~~YINTERNAL

# intercepts
SDQ_C_INT2 ~1
SDQ_C_INT1 ~1
YINTERNAL ~1
Hippocampus_c~1
SEX ~1
Covid_Age~1
Covid_Age2~1
IntraCranialVol~1
'

HippocampusMod1.fit <- sem(HippocampusMod1, estimator = "ML", missing ="ML",
data = dt.covid)
summary(HippocampusMod1.fit, standardized=TRUE, fit.measures = TRUE)
```

```
## lavaan 0.6-6 ended normally after 183 iterations
##
##   Estimator                                         ML
##   Optimization method                           NLMINB
##   Number of free parameters                         41
##
##   Number of observations                           145
##   Number of missing patterns                         6
##
## Model Test User Model:
##
##   Test statistic                                 3.509
##   Degrees of freedom                                 3
##   P-value (Chi-square)                           0.320
##
## Model Test Baseline Model:
##
##   Test statistic                               783.437
##   Degrees of freedom                                28
##   P-value                                        0.000
##
## User Model versus Baseline Model:
##
##   Comparative Fit Index (CFI)                    0.999
##   Tucker-Lewis Index (TLI)                       0.994
##
## Loglikelihood and Information Criteria:
##
##   Loglikelihood user model (H0)                 56.565
##   Loglikelihood unrestricted model (H1)         58.320
##
##   Akaike (AIC)                                 -31.130
##   Bayesian (BIC)                                90.916
##   Sample-size adjusted Bayesian (BIC)          -38.823
##
## Root Mean Square Error of Approximation:
##
##   RMSEA                                          0.034
##   90 Percent confidence interval - lower         0.000
##   90 Percent confidence interval - upper         0.148
##   P-value RMSEA <= 0.05                          0.470
##
## Standardized Root Mean Square Residual:
##
##   SRMR                                           0.005
##
## Parameter Estimates:
##
##   Standard errors                             Standard
##   Information                                 Observed
```

```
##   Observed information based on                Hessian
##
## Regressions:
##                    Estimate  Std.Err  z-value  P(>|z|)   Std.lv  Std.all
##   SDQ_C_INT2 ~
##     SDQ_C_INT1        0.703    0.102    6.911    0.000    0.703    0.550
##     Covid_Age2       -0.053    0.041   -1.287    0.198   -0.053   -0.093
##     Hippocampus_c     0.019    0.035    0.547    0.585    0.019    0.055
##     IntraCranialVl   -0.113    0.194   -0.582    0.561   -0.113   -0.061
##     SEX               0.076    0.049    1.564    0.118    0.076    0.142
##   SDQ_C_INT1 ~
##     YINTERNAL         0.222    0.076    2.903    0.004    0.222    0.239
##     Hippocampus_c    -0.027    0.029   -0.907    0.364   -0.027   -0.099
##     SEX               0.115    0.042    2.730    0.006    0.115    0.273
##     Covid_Age        -0.005    0.035   -0.136    0.892   -0.005   -0.011
##     IntraCranialVl   -0.138    0.165   -0.834    0.404   -0.138   -0.095
##   Covid_Age2 ~
##     Covid_Age         1.005    0.009  113.133    0.000    1.005    0.995
##
## Covariances:
##                      Estimate  Std.Err  z-value  P(>|z|)   Std.lv  Std.all
##   Hippocampus_c ~~
##     YINTERNAL           0.017    0.015    1.133    0.257    0.017    0.101
##   SEX ~~
##     YINTERNAL          -0.041    0.010   -4.197    0.000   -0.041   -0.373
##   Hippocampus_c ~~
##     SEX                -0.169    0.037   -4.574    0.000   -0.169   -0.442
##     Covid_Age          -0.008    0.032   -0.258    0.797   -0.008   -0.023
##  .Covid_Age2 ~~
##     Hippocampus_c       0.002    0.003    0.578    0.563    0.002    0.056
##   SEX ~~
##     Covid_Age           0.032    0.019    1.664    0.096    0.032    0.140
##  .Covid_Age2 ~~
##     SEX                 0.001    0.002    0.402    0.688    0.001    0.035
##   YINTERNAL ~~
##     Covid_Age          -0.015    0.009   -1.711    0.087   -0.015   -0.144
##  .SDQ_C_INT1 ~~
##    .Covid_Age2          0.003    0.001    2.982    0.003    0.003    0.294
##   Hippocampus_c ~~
##     IntraCranialVl      0.070    0.012    6.013    0.000    0.070    0.632
##   IntraCranialVol ~~
##     Covid_Age          -0.001    0.006   -0.102    0.918   -0.001   -0.009
##  .Covid_Age2 ~~
##     IntraCranialVl      0.000    0.001    0.213    0.831    0.000    0.020
##   IntraCranialVol ~~
##     SEX                -0.036    0.007   -5.161    0.000   -0.036   -0.507
##     YINTERNAL           0.003    0.003    0.972    0.331    0.003    0.086
##
## Intercepts:
##                    Estimate  Std.Err  z-value  P(>|z|)   Std.lv  Std.all
```

```
##    .SDQ_C_INT2        1.128    0.673    1.676    0.094    1.128    4.246
##    .SDQ_C_INT1        0.381    0.563    0.675    0.499    0.381    1.829
##     YINTERNAL         0.410    0.019   22.074    0.000    0.410    1.833
##     Hippocampus_c     0.007    0.069    0.105    0.916    0.007    0.009
##     SEX               0.434    0.041   10.566    0.000    0.434    0.877
##     Covid_Age        14.375    0.038  376.052    0.000   14.375   31.231
##    .Covid_Age2        0.511    0.128    4.004    0.000    0.511    1.100
##     IntraCranialVl    1.553    0.013  122.255    0.000    1.553   10.832
##
## Variances:
##                    Estimate  Std.Err  z-value  P(>|z|)   Std.lv  Std.all
##    .SDQ_C_INT2        0.044    0.006    7.662    0.000    0.044    0.624
##    .SDQ_C_INT1        0.037    0.004    8.410    0.000    0.037    0.845
##     YINTERNAL         0.050    0.006    8.515    0.000    0.050    1.000
##     Hippocampus_c     0.595    0.075    7.919    0.000    0.595    1.000
##     SEX               0.245    0.029    8.523    0.000    0.245    1.000
##     Covid_Age         0.212    0.025    8.512    0.000    0.212    1.000
##    .Covid_Age2        0.002    0.000    7.640    0.000    0.002    0.009
##     IntraCranialVl    0.021    0.003    7.948    0.000    0.021    1.000

#Hippocampal volume, interaction with pandemic-related stress
dt.covid$HipxStress1<-dt.covid$Hippocampus_c*dt.covid$COVID_Stress1
dt.covid$HipxStress2<-dt.covid$Hippocampus_c*dt.covid$COVID_Stress2
HippocampusMod2 <- '
# regressions
SDQ_C_INT2 ~ SDQ_C_INT1+Covid_Age2+Hippocampus_c+IntraCranialVol+COVID_Stress
2+HipxStress2+SEX
SDQ_C_INT1 ~ YINTERNAL + Hippocampus_c + SEX + Covid_Age+COVID_Stress1+IntraC
ranialVol+HipxStress1
Covid_Age2 ~ Covid_Age
COVID_Stress2~COVID_Stress1
HipxStress2~HipxStress1

# variances
SDQ_C_INT2 ~~ SDQ_C_INT2
SDQ_C_INT1 ~~ SDQ_C_INT1
YINTERNAL ~~ YINTERNAL
Hippocampus_c~~Hippocampus_c
SEX ~~ SEX
Covid_Age~~Covid_Age
Covid_Age2~~Covid_Age2
COVID_Stress1~~COVID_Stress1
COVID_Stress2~~COVID_Stress2
IntraCranialVol~~IntraCranialVol
HipxStress1~~HipxStress1
HipxStress2~~HipxStress2
# covariances
YINTERNAL~~Hippocampus_c
YINTERNAL~~SEX
SEX~~Hippocampus_c
```

```
Covid_Age~~Hippocampus_c
Covid_Age2~~Hippocampus_c
Covid_Age~~SEX
Covid_Age2~~SEX
Covid_Age~~YINTERNAL
Covid_Age2~~SDQ_C_INT1
COVID_Stress1~~YINTERNAL
COVID_Stress1~~SEX
COVID_Stress1~~Hippocampus_c
COVID_Stress1~~Covid_Age
COVID_Stress2~~SDQ_C_INT1
COVID_Stress2~~Covid_Age2
COVID_Stress2~~Hippocampus_c
COVID_Stress2~~SEX
COVID_Stress1~~IntraCranialVol
COVID_Stress2~~IntraCranialVol
Covid_Age~~IntraCranialVol
Covid_Age2~~IntraCranialVol
YINTERNAL ~~ IntraCranialVol
Hippocampus_c~~IntraCranialVol
SEX ~~ IntraCranialVol
COVID_Stress1~~HipxStress1
HipxStress1~~YINTERNAL
HipxStress1~~SEX
HipxStress1~~Hippocampus_c
HipxStress1~~Covid_Age
HipxStress2~~SDQ_C_INT1
HipxStress2~~Covid_Age2
HipxStress2~~Hippocampus_c
COVID_Stress2~~HipxStress2
HipxStress2~~SEX
HipxStress1~~IntraCranialVol
HipxStress2~~IntraCranialVol

# intercepts
SDQ_C_INT2 ~1
SDQ_C_INT1 ~1
YINTERNAL ~1
Hippocampus_c~1
SEX ~1
Covid_Age~1
Covid_Age2~1
COVID_Stress1~1
COVID_Stress2~1
IntraCranialVol~1
HipxStress1~1
HipxStress2~1
'
HippocampusMod2.fit <- sem(HippocampusMod2, estimator = "ML", missing ="ML",
```

```
data = dt.covid)
summary(HippocampusMod2.fit, standardized=TRUE, fit.measures = TRUE)
```

```
## lavaan 0.6-6 ended normally after 210 iterations
##
##   Estimator                                         ML
##   Optimization method                           NLMINB
##   Number of free parameters                         77
##
##   Number of observations                           145
##   Number of missing patterns                         7
##
## Model Test User Model:
##
##   Test statistic                                20.860
##   Degrees of freedom                                13
##   P-value (Chi-square)                           0.076
##
## Model Test Baseline Model:
##
##   Test statistic                               911.856
##   Degrees of freedom                                66
##   P-value                                        0.000
##
## User Model versus Baseline Model:
##
##   Comparative Fit Index (CFI)                    0.991
##   Tucker-Lewis Index (TLI)                       0.953
##
## Loglikelihood and Information Criteria:
##
##   Loglikelihood user model (H0)               -787.329
##   Loglikelihood unrestricted model (H1)       -776.899
##
##   Akaike (AIC)                                1728.658
##   Bayesian (BIC)                              1957.867
##   Sample-size adjusted Bayesian (BIC)         1714.211
##
## Root Mean Square Error of Approximation:
##
##   RMSEA                                          0.065
##   90 Percent confidence interval - lower         0.000
##   90 Percent confidence interval - upper         0.114
##   P-value RMSEA <= 0.05                          0.286
##
## Standardized Root Mean Square Residual:
##
##   SRMR                                           0.049
##
## Parameter Estimates:
```

```
##
##   Standard errors                             Standard
##   Information                                 Observed
##   Observed information based on                Hessian
##
## Regressions:
##                    Estimate  Std.Err  z-value  P(>|z|)   Std.lv  Std.all
##   SDQ_C_INT2 ~
##     SDQ_C_INT1        0.601    0.103    5.816    0.000    0.601    0.467
##     Covid_Age2       -0.039    0.040   -0.964    0.335   -0.039   -0.069
##     Hippocampus_c     0.035    0.034    1.031    0.302    0.035    0.104
##     IntraCranialVl   -0.109    0.186   -0.587    0.557   -0.109   -0.059
##     COVID_Stress2     0.038    0.012    3.098    0.002    0.038    0.240
##     HipxStress2      -0.009    0.016   -0.541    0.589   -0.009   -0.043
##     SEX               0.098    0.047    2.088    0.037    0.098    0.184
##   SDQ_C_INT1 ~
##     YINTERNAL         0.196    0.069    2.864    0.004    0.196    0.215
##     Hippocampus_c    -0.025    0.026   -0.977    0.329   -0.025   -0.096
##     SEX               0.104    0.037    2.809    0.005    0.104    0.252
##     Covid_Age         0.010    0.033    0.309    0.757    0.010    0.023
##     COVID_Stress1     0.047    0.008    5.806    0.000    0.047    0.401
##     IntraCranialVl   -0.145    0.146   -0.996    0.319   -0.145   -0.102
##     HipxStress1      -0.004    0.014   -0.274    0.784   -0.004   -0.021
##   Covid_Age2 ~
##     Covid_Age         1.007    0.009  111.785    0.000    1.007    0.995
##   COVID_Stress2 ~
##     COVID_Stress1     0.392    0.079    4.948    0.000    0.392    0.404
##   HipxStress2 ~
##     HipxStress1       0.357    0.104    3.445    0.001    0.357    0.310
##
## Covariances:
##                      Estimate  Std.Err  z-value  P(>|z|)   Std.lv  Std.all
##   Hippocampus_c ~~
##     YINTERNAL           0.019    0.014    1.313    0.189    0.019    0.109
##   SEX ~~
##     YINTERNAL          -0.041    0.010   -4.199    0.000   -0.041   -0.374
##   Hippocampus_c ~~
##     SEX                -0.168    0.037   -4.582    0.000   -0.168   -0.439
##     Covid_Age          -0.000    0.030   -0.013    0.990   -0.000   -0.001
##  .Covid_Age2 ~~
##     Hippocampus_c       0.002    0.003    0.645    0.519    0.002    0.062
##   SEX ~~
##     Covid_Age           0.029    0.019    1.518    0.129    0.029    0.129
##  .Covid_Age2 ~~
##     SEX                 0.001    0.002    0.377    0.706    0.001    0.033
##   YINTERNAL ~~
##     Covid_Age          -0.015    0.009   -1.711    0.087   -0.015   -0.144
##  .SDQ_C_INT1 ~~
##    .Covid_Age2          0.002    0.001    2.401    0.016    0.002    0.231
##   YINTERNAL ~~
```

```
##     COVID_Stress1        0.001    0.032    0.045    0.964    0.001    0.004
##   SEX ~~
##     COVID_Stress1        0.040    0.072    0.549    0.583    0.040    0.046
##   Hippocampus_c ~~
##     COVID_Stress1       -0.064    0.115   -0.552    0.581   -0.064   -0.047
##   Covid_Age ~~
##     COVID_Stress1       -0.026    0.066   -0.398    0.691   -0.026   -0.033
##  .SDQ_C_INT1 ~~
##    .COVID_Stress2        0.019    0.022    0.864    0.388    0.019    0.074
##  .Covid_Age2 ~~
##    .COVID_Stress2        0.009    0.006    1.415    0.157    0.009    0.133
##  .COVID_Stress2 ~~
##     Hippocampus_c       -0.375    0.112   -3.342    0.001   -0.375   -0.315
##     SEX                  0.056    0.064    0.875    0.382    0.056    0.074
##   IntraCranialVol ~~
##     COVID_Stress1       -0.010    0.022   -0.486    0.627   -0.010   -0.042
##  .COVID_Stress2 ~~
##     IntraCranialVl      -0.041    0.020   -2.064    0.039   -0.041   -0.186
##   IntraCranialVol ~~
##     Covid_Age            0.001    0.006    0.168    0.866    0.001    0.015
##  .Covid_Age2 ~~
##     IntraCranialVl       0.000    0.001    0.293    0.769    0.000    0.027
##   IntraCranialVol ~~
##     YINTERNAL            0.003    0.003    0.991    0.322    0.003    0.085
##   Hippocampus_c ~~
##     IntraCranialVl       0.070    0.012    6.017    0.000    0.070    0.633
##   IntraCranialVol ~~
##     SEX                 -0.036    0.007   -5.132    0.000   -0.036   -0.503
##   COVID_Stress1 ~~
##     HipxStress1         -0.111    0.176   -0.628    0.530   -0.111   -0.056
##   YINTERNAL ~~
##     HipxStress1         -0.015    0.023   -0.635    0.526   -0.015   -0.057
##   SEX ~~
##     HipxStress1          0.037    0.052    0.710    0.478    0.037    0.065
##   Hippocampus_c ~~
##     HipxStress1         -0.055    0.076   -0.723    0.469   -0.055   -0.062
##   Covid_Age ~~
##     HipxStress1         -0.102    0.048   -2.110    0.035   -0.102   -0.194
##  .SDQ_C_INT1 ~~
##    .HipxStress2         -0.017    0.020   -0.888    0.374   -0.017   -0.082
##  .Covid_Age2 ~~
##    .HipxStress2          0.001    0.006    0.112    0.911    0.001    0.011
##  .HipxStress2 ~~
##     Hippocampus_c       -0.228    0.093   -2.444    0.015   -0.228   -0.236
##  .COVID_Stress2 ~~
##    .HipxStress2         -0.048    0.183   -0.264    0.792   -0.048   -0.025
##  .HipxStress2 ~~
##     SEX                  0.071    0.056    1.259    0.208    0.071    0.115
##   IntraCranialVol ~~
##     HipxStress1         -0.013    0.015   -0.863    0.388   -0.013   -0.077
```

```
##  .HipxStress2 ~~
##     IntraCranialVl     -0.036    0.017   -2.145    0.032   -0.036   -0.202
##
## Intercepts:
##                    Estimate  Std.Err  z-value  P(>|z|)   Std.lv  Std.all
##    .SDQ_C_INT2        0.921    0.650    1.418    0.156    0.921    3.500
##    .SDQ_C_INT1        0.193    0.519    0.372    0.710    0.193    0.942
##     YINTERNAL         0.410    0.019   22.074    0.000    0.410    1.833
##     Hippocampus_c     0.008    0.068    0.113    0.910    0.008    0.010
##     SEX               0.434    0.041   10.567    0.000    0.434    0.878
##     Covid_Age        14.375    0.038  376.031    0.000   14.375   31.230
##    .Covid_Age2        0.474    0.130    3.660    0.000    0.474    1.018
##     COVID_Stress1    -0.031    0.144   -0.215    0.829   -0.031   -0.018
##    .COVID_Stress2    -0.082    0.138   -0.597    0.550   -0.082   -0.049
##     IntraCranialVl    1.552    0.013  122.218    0.000    1.552   10.828
##     HipxStress1      -0.052    0.103   -0.499    0.618   -0.052   -0.045
##    .HipxStress2      -0.419    0.120   -3.478    0.001   -0.419   -0.319
##
## Variances:
##                    Estimate  Std.Err  z-value  P(>|z|)   Std.lv  Std.all
##    .SDQ_C_INT2        0.041    0.005    7.652    0.000    0.041    0.587
##    .SDQ_C_INT1        0.029    0.003    8.354    0.000    0.029    0.682
##     YINTERNAL         0.050    0.006    8.515    0.000    0.050    1.000
##     Hippocampus_c     0.595    0.075    7.928    0.000    0.595    1.000
##     SEX               0.245    0.029    8.501    0.000    0.245    1.000
##     Covid_Age         0.212    0.025    8.512    0.000    0.212    1.000
##    .Covid_Age2        0.002    0.000    7.712    0.000    0.002    0.009
##     COVID_Stress1     3.017    0.354    8.515    0.000    3.017    1.000
##    .COVID_Stress2     2.380    0.300    7.926    0.000    2.380    0.837
##     IntraCranialVl    0.021    0.003    7.947    0.000    0.021    1.000
##     HipxStress1       1.308    0.167    7.846    0.000    1.308    1.000
##    .HipxStress2       1.561    0.213    7.344    0.000    1.561    0.904

#Amygdala, main effects
AmygdalaMod1 <- '
# regressions
SDQ_C_INT2 ~ SDQ_C_INT1+Covid_Age2+Amygdala_c+IntraCranialVol+SEX
SDQ_C_INT1 ~ YINTERNAL + Amygdala_c + SEX + Covid_Age+IntraCranialVol
Covid_Age2 ~ Covid_Age

# variances
SDQ_C_INT2 ~~ SDQ_C_INT2
SDQ_C_INT1 ~~ SDQ_C_INT1
YINTERNAL ~~ YINTERNAL
Amygdala_c~~Amygdala_c
SEX ~~ SEX
Covid_Age~~Covid_Age
Covid_Age2~~Covid_Age2
IntraCranialVol~~IntraCranialVol
```

```
# covariances
YINTERNAL~~Amygdala_c
YINTERNAL~~SEX
SEX~~Amygdala_c
Covid_Age~~Amygdala_c
Covid_Age2~~Amygdala_c
Covid_Age~~SEX
Covid_Age2~~SEX
Covid_Age~~YINTERNAL
Covid_Age2~~SDQ_C_INT1
IntraCranialVol~~Amygdala_c
IntraCranialVol~~Covid_Age
IntraCranialVol~~Covid_Age2
IntraCranialVol~~SEX
IntraCranialVol~~YINTERNAL

# intercepts
SDQ_C_INT2 ~1
SDQ_C_INT1 ~1
YINTERNAL ~1
Amygdala_c~1
SEX ~1
Covid_Age~1
Covid_Age2~1
IntraCranialVol~1
'

AmygdalaMod1.fit <- sem(AmygdalaMod1, estimator = "ML", missing ="ML", data =
dt.covid)
summary(AmygdalaMod1.fit, standardized=TRUE, fit.measures = TRUE)

## lavaan 0.6-6 ended normally after 198 iterations
##
##   Estimator                                         ML
##   Optimization method                           NLMINB
##   Number of free parameters                         41
##
##   Number of observations                           145
##   Number of missing patterns                         6
##
## Model Test User Model:
##
##   Test statistic                                 3.599
##   Degrees of freedom                                 3
##   P-value (Chi-square)                           0.308
##
## Model Test Baseline Model:
##
##   Test statistic                               792.012
##   Degrees of freedom                                28
```

```
##   P-value                                       0.000
##
## User Model versus Baseline Model:
##
##   Comparative Fit Index (CFI)                   0.999
##   Tucker-Lewis Index (TLI)                      0.993
##
## Loglikelihood and Information Criteria:
##
##   Loglikelihood user model (H0)               156.353
##   Loglikelihood unrestricted model (H1)       158.152
##
##   Akaike (AIC)                               -230.705
##   Bayesian (BIC)                             -108.659
##   Sample-size adjusted Bayesian (BIC)        -238.398
##
## Root Mean Square Error of Approximation:
##
##   RMSEA                                         0.037
##   90 Percent confidence interval - lower        0.000
##   90 Percent confidence interval - upper        0.150
##   P-value RMSEA <= 0.05                         0.459
##
## Standardized Root Mean Square Residual:
##
##   SRMR                                          0.006
##
## Parameter Estimates:
##
##   Standard errors                            Standard
##   Information                                Observed
##   Observed information based on               Hessian
##
## Regressions:
##                    Estimate  Std.Err  z-value  P(>|z|)   Std.lv  Std.all
##   SDQ_C_INT2 ~
##     SDQ_C_INT1        0.707    0.102    6.936    0.000    0.707    0.554
##     Covid_Age2       -0.054    0.041   -1.305    0.192   -0.054   -0.094
##     Amygdala_c        0.060    0.077    0.773    0.439    0.060    0.079
##     IntraCranialVl   -0.134    0.192   -0.700    0.484   -0.134   -0.072
##     SEX               0.079    0.049    1.613    0.107    0.079    0.147
##   SDQ_C_INT1 ~
##     YINTERNAL         0.214    0.077    2.776    0.005    0.214    0.231
##     Amygdala_c       -0.047    0.067   -0.709    0.478   -0.047   -0.080
##     SEX               0.113    0.043    2.628    0.009    0.113    0.269
##     Covid_Age        -0.004    0.036   -0.112    0.911   -0.004   -0.009
##     IntraCranialVl   -0.154    0.166   -0.927    0.354   -0.154   -0.106
##   Covid_Age2 ~
##     Covid_Age         1.005    0.009  113.161    0.000    1.005    0.995
##
```

```
## Covariances:
##                    Estimate  Std.Err  z-value  P(>|z|)   Std.lv  Std.all
##   Amygdala_c ~~
##     YINTERNAL          0.003    0.007    0.497    0.619    0.003    0.044
##   SEX ~~
##     YINTERNAL         -0.041    0.010   -4.198    0.000   -0.041   -0.373
##   Amygdala_c ~~
##     SEX               -0.084    0.017   -4.953    0.000   -0.084   -0.483
##     Covid_Age         -0.002    0.014   -0.167    0.868   -0.002   -0.015
##  .Covid_Age2 ~~
##     Amygdala_c         0.000    0.002    0.216    0.829    0.000    0.021
##   SEX ~~
##     Covid_Age          0.032    0.019    1.664    0.096    0.032    0.140
##  .Covid_Age2 ~~
##     SEX                0.001    0.002    0.392    0.695    0.001    0.034
##   YINTERNAL ~~
##     Covid_Age         -0.015    0.009   -1.712    0.087   -0.015   -0.144
##  .SDQ_C_INT1 ~~
##    .Covid_Age2         0.002    0.001    2.923    0.003    0.002    0.288
##   Amygdala_c ~~
##     IntraCranialVl     0.033    0.005    6.136    0.000    0.033    0.649
##   IntraCranialVol ~~
##     Covid_Age         -0.001    0.006   -0.102    0.919   -0.001   -0.009
##  .Covid_Age2 ~~
##     IntraCranialVl     0.000    0.001    0.219    0.827    0.000    0.020
##   IntraCranialVol ~~
##     SEX               -0.036    0.007   -5.161    0.000   -0.036   -0.507
##     YINTERNAL          0.003    0.003    0.973    0.331    0.003    0.086
##
## Intercepts:
##                  Estimate  Std.Err  z-value  P(>|z|)   Std.lv  Std.all
##    .SDQ_C_INT2      0.987    0.651    1.516    0.129    0.987    3.715
##    .SDQ_C_INT1      0.542    0.553    0.980    0.327    0.542    2.605
##     YINTERNAL       0.410    0.019   22.074    0.000    0.410    1.833
##     Amygdala_c      3.076    0.031   98.341    0.000    3.076    8.727
##     SEX             0.434    0.041   10.566    0.000    0.434    0.877
##     Covid_Age      14.375    0.038  376.011    0.000   14.375   31.228
##    .Covid_Age2      0.510    0.128    3.997    0.000    0.510    1.098
##     IntraCranialVl  1.553    0.013  122.247    0.000    1.553   10.832
##
## Variances:
##                  Estimate  Std.Err  z-value  P(>|z|)   Std.lv  Std.all
##    .SDQ_C_INT2      0.044    0.006    7.659    0.000    0.044    0.623
##    .SDQ_C_INT1      0.037    0.004    8.414    0.000    0.037    0.848
##     YINTERNAL       0.050    0.006    8.515    0.000    0.050    1.000
##     Amygdala_c      0.124    0.016    7.935    0.000    0.124    1.000
##     SEX             0.245    0.029    8.522    0.000    0.245    1.000
##     Covid_Age       0.212    0.025    8.512    0.000    0.212    1.000
##    .Covid_Age2      0.002    0.000    7.648    0.000    0.002    0.009
##     IntraCranialVl  0.021    0.003    7.948    0.000    0.021    1.000
```

```
#Amygdala volume, interaction with pandemic-related stress
dt.covid$AmyxStress1<-dt.covid$Amygdala_c*dt.covid$COVID_Stress1
dt.covid$AmyxStress2<-dt.covid$Amygdala_c*dt.covid$COVID_Stress2
AmygdalaMod2 <- '
# regressions
SDQ_C_INT2 ~ SDQ_C_INT1+Covid_Age2+Amygdala_c+IntraCranialVol+COVID_Stress2+A
myxStress2+SEX
SDQ_C_INT1 ~ YINTERNAL + Amygdala_c + SEX + Covid_Age+COVID_Stress1+IntraCran
ialVol+AmyxStress1
Covid_Age2 ~ Covid_Age
COVID_Stress2~COVID_Stress1
AmyxStress2~AmyxStress1

# variances
SDQ_C_INT2 ~~ SDQ_C_INT2
SDQ_C_INT1 ~~ SDQ_C_INT1
YINTERNAL ~~ YINTERNAL
Amygdala_c~~Amygdala_c
SEX ~~ SEX
Covid_Age~~Covid_Age
Covid_Age2~~Covid_Age2
COVID_Stress1~~COVID_Stress1
COVID_Stress2~~COVID_Stress2
IntraCranialVol~~IntraCranialVol
AmyxStress1~~AmyxStress1
AmyxStress2~~AmyxStress2
# covariances
YINTERNAL~~Amygdala_c
YINTERNAL~~SEX
SEX~~Amygdala_c
Covid_Age~~Amygdala_c
Covid_Age2~~Amygdala_c
Covid_Age~~SEX
Covid_Age2~~SEX
Covid_Age~~YINTERNAL
Covid_Age2~~SDQ_C_INT1
COVID_Stress1~~YINTERNAL
COVID_Stress1~~SEX
COVID_Stress1~~Amygdala_c
COVID_Stress1~~Covid_Age
COVID_Stress2~~SDQ_C_INT1
COVID_Stress2~~Covid_Age2
COVID_Stress2~~Amygdala_c
COVID_Stress2~~SEX
COVID_Stress1~~IntraCranialVol
COVID_Stress2~~IntraCranialVol
Covid_Age~~IntraCranialVol
Covid_Age2~~IntraCranialVol
YINTERNAL ~~ IntraCranialVol
Amygdala_c~~IntraCranialVol
```

```
SEX ~~ IntraCranialVol
COVID_Stress1~~AmyxStress1
AmyxStress1~~YINTERNAL
AmyxStress1~~SEX
AmyxStress1~~Amygdala_c
AmyxStress1~~Covid_Age
AmyxStress2~~SDQ_C_INT1
AmyxStress2~~Covid_Age2
AmyxStress2~~Amygdala_c
COVID_Stress2~~AmyxStress2
AmyxStress2~~SEX
AmyxStress1~~IntraCranialVol
AmyxStress2~~IntraCranialVol

# intercepts
SDQ_C_INT2 ~1
SDQ_C_INT1 ~1
YINTERNAL ~1
Amygdala_c~1
SEX ~1
Covid_Age~1
Covid_Age2~1
COVID_Stress1~1
COVID_Stress2~1
IntraCranialVol~1
AmyxStress1~1
AmyxStress2~1
'
AmygdalaMod2.fit <- sem(AmygdalaMod2, estimator = "ML", missing ="ML", data =
dt.covid)
summary(AmygdalaMod2.fit, standardized=TRUE, fit.measures = TRUE)

## lavaan 0.6-6 ended normally after 328 iterations
##
##   Estimator                                         ML
##   Optimization method                           NLMINB
##   Number of free parameters                         77
##
##   Number of observations                           145
##   Number of missing patterns                         7
##
## Model Test User Model:
##
##   Test statistic                                17.039
##   Degrees of freedom                                13
##   P-value (Chi-square)                           0.198
##
## Model Test Baseline Model:
##
##   Test statistic                              1947.462
```

```
##   Degrees of freedom                                66
##   P-value                                        0.000
##
## User Model versus Baseline Model:
##
##   Comparative Fit Index (CFI)                    0.998
##   Tucker-Lewis Index (TLI)                       0.989
##
## Loglikelihood and Information Criteria:
##
##   Loglikelihood user model (H0)               -511.786
##   Loglikelihood unrestricted model (H1)       -503.266
##
##   Akaike (AIC)                                1177.572
##   Bayesian (BIC)                              1406.781
##   Sample-size adjusted Bayesian (BIC)         1163.125
##
## Root Mean Square Error of Approximation:
##
##   RMSEA                                          0.046
##   90 Percent confidence interval - lower         0.000
##   90 Percent confidence interval - upper         0.100
##   P-value RMSEA <= 0.05                          0.492
##
## Standardized Root Mean Square Residual:
##
##   SRMR                                           0.031
##
## Parameter Estimates:
##
##   Standard errors                             Standard
##   Information                                 Observed
##   Observed information based on                Hessian
##
## Regressions:
##                    Estimate  Std.Err  z-value  P(>|z|)   Std.lv  Std.all
##   SDQ_C_INT2 ~
##     SDQ_C_INT1        0.604    0.105    5.763    0.000    0.604    0.468
##     Covid_Age2       -0.041    0.040   -1.028    0.304   -0.041   -0.072
##     Amygdala_c        0.137    0.079    1.722    0.085    0.137    0.182
##     IntraCranialVl   -0.157    0.182   -0.862    0.389   -0.157   -0.085
##     COVID_Stress2     0.059    0.111    0.528    0.598    0.059    0.378
##     AmyxStress2      -0.005    0.037   -0.141    0.888   -0.005   -0.103
##     SEX               0.108    0.048    2.253    0.024    0.108    0.202
##   SDQ_C_INT1 ~
##     YINTERNAL         0.193    0.068    2.824    0.005    0.193    0.212
##     Amygdala_c       -0.010    0.058   -0.166    0.868   -0.010   -0.017
##     SEX               0.118    0.038    3.105    0.002    0.118    0.286
##     Covid_Age        -0.005    0.033   -0.145    0.885   -0.005   -0.011
##     COVID_Stress1     0.183    0.078    2.341    0.019    0.183    1.560
```

```
##     IntraCranialVl   -0.181    0.145   -1.244    0.213   -0.181   -0.127
##     AmyxStress1      -0.044    0.025   -1.734    0.083   -0.044   -1.156
##   Covid_Age2 ~
##     Covid_Age         1.009    0.009  113.715    0.000    1.009    0.995
##   COVID_Stress2 ~
##     COVID_Stress1     0.409    0.056    7.311    0.000    0.409    0.419
##   AmyxStress2 ~
##     AmyxStress1       0.395    0.056    7.121    0.000    0.395    0.411
##
## Covariances:
##                      Estimate  Std.Err  z-value  P(>|z|)   Std.lv  Std.all
##   Amygdala_c ~~
##     YINTERNAL           0.005    0.006    0.819    0.413    0.005    0.066
##   SEX ~~
##     YINTERNAL          -0.042    0.010   -4.250    0.000   -0.042   -0.375
##   Amygdala_c ~~
##     SEX                -0.085    0.017   -5.052    0.000   -0.085   -0.489
##     Covid_Age          -0.002    0.013   -0.131    0.896   -0.002   -0.011
##  .Covid_Age2 ~~
##     Amygdala_c          0.001    0.001    0.362    0.717    0.001    0.034
##   SEX ~~
##     Covid_Age           0.029    0.019    1.543    0.123    0.029    0.129
##  .Covid_Age2 ~~
##     SEX                 0.001    0.002    0.440    0.660    0.001    0.039
##   YINTERNAL ~~
##     Covid_Age          -0.015    0.009   -1.711    0.087   -0.015   -0.144
##  .SDQ_C_INT1 ~~
##    .Covid_Age2          0.002    0.001    2.242    0.025    0.002    0.217
##   YINTERNAL ~~
##     COVID_Stress1       0.001    0.032    0.045    0.964    0.001    0.004
##   SEX ~~
##     COVID_Stress1       0.033    0.072    0.452    0.651    0.033    0.038
##   Amygdala_c ~~
##     COVID_Stress1      -0.064    0.052   -1.230    0.219   -0.064   -0.105
##   Covid_Age ~~
##     COVID_Stress1      -0.026    0.066   -0.396    0.692   -0.026   -0.033
##  .SDQ_C_INT1 ~~
##    .COVID_Stress2       0.020    0.022    0.904    0.366    0.020    0.077
##  .Covid_Age2 ~~
##    .COVID_Stress2       0.009    0.006    1.437    0.151    0.009    0.134
##  .COVID_Stress2 ~~
##     Amygdala_c         -0.186    0.051   -3.657    0.000   -0.186   -0.345
##     SEX                 0.059    0.064    0.909    0.363    0.059    0.077
##   IntraCranialVol ~~
##     COVID_Stress1      -0.007    0.022   -0.315    0.753   -0.007   -0.027
##  .COVID_Stress2 ~~
##     IntraCranialVl     -0.040    0.020   -2.005    0.045   -0.040   -0.182
##   IntraCranialVol ~~
##     Covid_Age          -0.000    0.006   -0.028    0.978   -0.000   -0.002
##  .Covid_Age2 ~~
```

```
##     IntraCranialVl      0.000    0.001    0.286    0.775    0.000    0.026
##   IntraCranialVol ~~
##     YINTERNAL           0.003    0.003    1.087    0.277    0.003    0.094
##   Amygdala_c ~~
##     IntraCranialVl      0.033    0.005    6.154    0.000    0.033    0.649
##   IntraCranialVol ~~
##     SEX                -0.036    0.007   -5.183    0.000   -0.036   -0.509
##   COVID_Stress1 ~~
##     AmyxStress1         9.270    1.093    8.483    0.000    9.270    0.993
##   YINTERNAL ~~
##     AmyxStress1        -0.005    0.100   -0.048    0.961   -0.005   -0.004
##   SEX ~~
##     AmyxStress1         0.130    0.223    0.583    0.560    0.130    0.049
##   Amygdala_c ~~
##     AmyxStress1        -0.199    0.162   -1.231    0.218   -0.199   -0.105
##   Covid_Age ~~
##     AmyxStress1        -0.145    0.206   -0.702    0.483   -0.145   -0.058
##  .SDQ_C_INT1 ~~
##    .AmyxStress2         0.054    0.066    0.814    0.416    0.054    0.068
##  .Covid_Age2 ~~
##    .AmyxStress2         0.025    0.020    1.287    0.198    0.025    0.120
##  .AmyxStress2 ~~
##     Amygdala_c         -0.616    0.157   -3.924    0.000   -0.616   -0.372
##  .COVID_Stress2 ~~
##    .AmyxStress2         7.218    0.910    7.928    0.000    7.218    0.994
##  .AmyxStress2 ~~
##     SEX                 0.224    0.197    1.136    0.256    0.224    0.096
##   IntraCranialVol ~~
##     AmyxStress1        -0.021    0.067   -0.309    0.758   -0.021   -0.027
##  .AmyxStress2 ~~
##     IntraCranialVl     -0.135    0.061   -2.208    0.027   -0.135   -0.200
##
## Intercepts:
##                    Estimate  Std.Err  z-value  P(>|z|)   Std.lv  Std.all
##    .SDQ_C_INT2        0.605    0.634    0.954    0.340    0.605    2.297
##    .SDQ_C_INT1        0.483    0.497    0.971    0.331    0.483    2.363
##     YINTERNAL         0.410    0.019   22.074    0.000    0.410    1.833
##     Amygdala_c        3.077    0.031   99.343    0.000    3.077    8.764
##     SEX               0.434    0.041   10.566    0.000    0.434    0.877
##     Covid_Age        14.375    0.038  376.002    0.000   14.375   31.227
##    .Covid_Age2        0.446    0.128    3.495    0.000    0.446    0.955
##     COVID_Stress1    -0.031    0.144   -0.215    0.829   -0.031   -0.018
##    .COVID_Stress2    -0.086    0.137   -0.627    0.531   -0.086   -0.051
##     IntraCranialVl    1.552    0.013  122.365    0.000    1.552   10.842
##     AmyxStress1      -0.174    0.447   -0.389    0.698   -0.174   -0.032
##    .AmyxStress2      -0.477    0.420   -1.134    0.257   -0.477   -0.092
##
## Variances:
##                    Estimate  Std.Err  z-value  P(>|z|)   Std.lv  Std.all
##    .SDQ_C_INT2        0.040    0.005    7.639    0.000    0.040    0.578
```

```
##    .SDQ_C_INT1        0.028    0.003    8.334    0.000    0.028    0.667
##     YINTERNAL         0.050    0.006    8.515    0.000    0.050    1.000
##     Amygdala_c        0.123    0.015    7.977    0.000    0.123    1.000
##     SEX               0.245    0.029    8.524    0.000    0.245    1.000
##     Covid_Age         0.212    0.025    8.512    0.000    0.212    1.000
##    .Covid_Age2        0.002    0.000    7.704    0.000    0.002    0.009
##     COVID_Stress1     3.017    0.354    8.515    0.000    3.017    1.000
##    .COVID_Stress2     2.373    0.298    7.950    0.000    2.373    0.825
##     IntraCranialVl    0.020    0.003    7.964    0.000    0.020    1.000
##     AmyxStress1      28.867    3.396    8.500    0.000   28.867    1.000
##    .AmyxStress2      22.230    2.796    7.951    0.000   22.230    0.831
```

```
#FDR correction on analyses of hippocampal and amygdala volume and internaliz
ing at T1
p.adjust(c(.364,.784,.478,.083),method="fdr")
```

```
## [1] 0.6373333 0.7840000 0.6373333 0.3320000
```

```
#FDR correction on analyses of hippocampal and amygdala volume and internaliz
ing at T2
p.adjust(c(.585,.589,.439,.888),method="fdr")
```

```
## [1] 0.7853333 0.7853333 0.7853333 0.8880000
```

Sensitivity Analyses of associations between amygdala activation and internalizing problems using Harvard-Oxford Atlas ROIs thresholded at 50% probability

```
lamod1 <- summary(lm(SDQ_C_INT ~ LAmyg50.Fear_GT_Calm + SEX + YINTERNAL + Cov
id_Age,
    data = dt.covid))
ramod1 <- summary(lm(SDQ_C_INT ~ RAmyg50.Fear_GT_Calm + SEX + YINTERNAL + Cov
id_Age,
    data = dt.covid))
lamod1
```

```
##
## Call:
## lm(formula = SDQ_C_INT ~ LAmyg50.Fear_GT_Calm + SEX + YINTERNAL +
##     Covid_Age, data = dt.covid)
##
## Residuals:
##     Min      1Q  Median      3Q     Max
## -6.3128 -2.1156 -0.5472  1.4738 10.2205
##
## Coefficients:
##                      Estimate Std. Error t value Pr(>|t|)
## (Intercept)           -4.8989     8.8653  -0.553  0.58162
## LAmyg50.Fear_GT_Calm  -0.8822     0.2983  -2.957  0.00377 **
## SEX                    2.7770     0.6075   4.571 1.23e-05 ***
```

```
## YINTERNAL              3.3111     1.3402   2.471  0.01495 *
## Covid_Age              0.4443     0.6132   0.725  0.47018
## ---
## Signif. codes:  0 '***' 0.001 '**' 0.01 '*' 0.05 '.' 0.1 ' ' 1
##
## Residual standard error: 3.075 on 115 degrees of freedom
##   (25 observations deleted due to missingness)
## Multiple R-squared:  0.2258, Adjusted R-squared:  0.1989
## F-statistic: 8.387 on 4 and 115 DF,  p-value: 5.671e-06
```

```
lm.beta(lm(SDQ_C_INT ~ LAmyg50.Fear_GT_Calm + SEX + YINTERNAL + Covid_Age, da
ta = dt.covid))
```

```
##
## Call:
## lm(formula = SDQ_C_INT ~ LAmyg50.Fear_GT_Calm + SEX + YINTERNAL +
##     Covid_Age, data = dt.covid)
##
## Standardized Coefficients::
##          (Intercept) LAmyg50.Fear_GT_Calm                  SEX
##           0.00000000          -0.24417848           0.40310208
##            YINTERNAL            Covid_Age
##           0.21711896           0.05964765
```

```
ramod1
```

```
##
## Call:
## lm(formula = SDQ_C_INT ~ RAmyg50.Fear_GT_Calm + SEX + YINTERNAL +
##     Covid_Age, data = dt.covid)
##
## Residuals:
##     Min      1Q  Median      3Q     Max
## -6.4105 -2.1705 -0.3626  1.3927 11.7875
##
## Coefficients:
##                        Estimate Std. Error t value Pr(>|t|)
## (Intercept)             -3.7426     9.1971  -0.407   0.6848
## RAmyg50.Fear_GT_Calm    -0.1661     0.3169  -0.524   0.6012
## SEX                      2.9595     0.6270   4.720  6.7e-06 ***
## YINTERNAL                3.3162     1.3913   2.384   0.0188 *
## Covid_Age                0.3457     0.6359   0.544   0.5878
## ---
## Signif. codes:  0 '***' 0.001 '**' 0.01 '*' 0.05 '.' 0.1 ' ' 1
##
## Residual standard error: 3.186 on 115 degrees of freedom
##   (25 observations deleted due to missingness)
## Multiple R-squared:  0.1689, Adjusted R-squared:   0.14
## F-statistic: 5.845 on 4 and 115 DF,  p-value: 0.0002562
```

```
lm.beta(lm(SDQ_C_INT ~ RAmyg50.Fear_GT_Calm + SEX + YINTERNAL + Covid_Age, da
ta = dt.covid))

##
## Call:
## lm(formula = SDQ_C_INT ~ RAmyg50.Fear_GT_Calm + SEX + YINTERNAL +
##     Covid_Age, data = dt.covid)
##
## Standardized Coefficients::
##          (Intercept) RAmyg50.Fear_GT_Calm                  SEX
##           0.00000000          -0.04474336           0.42959150
##            YINTERNAL            Covid_Age
##           0.21745497           0.04640782

laxmod1 <- summary(lm(SDQ_C_INT ~ LAmyg50.Fear_GT_Calm_c * COVID_Stress1 + SE
X +
    YINTERNAL + Covid_Age, data = dt.covid))
raxmod1 <- summary(lm(SDQ_C_INT ~ RAmyg50.Fear_GT_Calm_c * COVID_Stress1 + SE
X +
    YINTERNAL + Covid_Age, data = dt.covid))
laxmod1

##
## Call:
## lm(formula = SDQ_C_INT ~ LAmyg50.Fear_GT_Calm_c * COVID_Stress1 +
##     SEX + YINTERNAL + Covid_Age, data = dt.covid)
##
## Residuals:
##     Min      1Q  Median      3Q     Max
## -6.1803 -1.7320 -0.4627  1.4677  9.2369
##
## Coefficients:
##                                      Estimate Std. Error t value Pr(>|t|)
## (Intercept)                           -6.4904     7.8738  -0.824   0.4115
## LAmyg50.Fear_GT_Calm_c                -0.4908     0.2739  -1.792   0.0758
.
## COVID_Stress1                          0.7179     0.1481   4.848 4.00e-06
***
## SEX                                    2.4320     0.5443   4.468 1.88e-05
***
## YINTERNAL                              2.6719     1.1981   2.230   0.0277
*
## Covid_Age                              0.5516     0.5445   1.013   0.3133
## LAmyg50.Fear_GT_Calm_c:COVID_Stress1  -0.3762     0.1638  -2.296   0.0235
*
## ---
## Signif. codes:  0 '***' 0.001 '**' 0.01 '*' 0.05 '.' 0.1 ' ' 1
##
## Residual standard error: 2.729 on 113 degrees of freedom
##   (25 observations deleted due to missingness)
```

```
## Multiple R-squared:  0.4009, Adjusted R-squared:  0.3691
## F-statistic:  12.6 on 6 and 113 DF,  p-value: 7.65e-11
```

```r
lm.beta(lm(SDQ_C_INT ~ LAmyg50.Fear_GT_Calm_c * COVID_Stress1 + SEX + YINTERN
AL +
    Covid_Age, data = dt.covid))
```

```
##
## Call:
## lm(formula = SDQ_C_INT ~ LAmyg50.Fear_GT_Calm_c * COVID_Stress1 +
##     SEX + YINTERNAL + Covid_Age, data = dt.covid)
##
## Standardized Coefficients::
##                          (Intercept)               LAmyg50.Fear_GT_Calm_c
##                           0.00000000                          -0.13583510
##                        COVID_Stress1                                  SEX
##                           0.37121595                           0.35302557
##                            YINTERNAL                            Covid_Age
##                           0.17520465                           0.07404245
## LAmyg50.Fear_GT_Calm_c:COVID_Stress1
##                          -0.17191967
```

```r
raxmod1
```

```
##
## Call:
## lm(formula = SDQ_C_INT ~ RAmyg50.Fear_GT_Calm_c * COVID_Stress1 +
##     SEX + YINTERNAL + Covid_Age, data = dt.covid)
##
## Residuals:
##    Min     1Q Median     3Q    Max
## -6.562 -1.742 -0.292  1.323  8.747
##
## Coefficients:
##                                      Estimate Std. Error t value Pr(>|t|)
## (Intercept)                          -6.27299    8.16691  -0.768   0.4440
## RAmyg50.Fear_GT_Calm_c                0.28430    0.29532   0.963   0.3378
## COVID_Stress1                         0.88313    0.15678   5.633 1.31e-07
***
## SEX                                   2.62996    0.56097   4.688 7.76e-06
***
## YINTERNAL                             2.82179    1.23544   2.284   0.0242
*
## Covid_Age                             0.53791    0.56521   0.952   0.3433
## RAmyg50.Fear_GT_Calm_c:COVID_Stress1  0.01647    0.15512   0.106   0.9156
## ---
## Signif. codes:  0 '***' 0.001 '**' 0.01 '*' 0.05 '.' 0.1 ' ' 1
##
## Residual standard error: 2.822 on 113 degrees of freedom
##   (25 observations deleted due to missingness)
```

```
## Multiple R-squared:  0.3592, Adjusted R-squared:  0.3252
## F-statistic: 10.56 on 6 and 113 DF,  p-value: 2.769e-09
```

```
lm.beta(lm(SDQ_C_INT ~ RAmyg50.Fear_GT_Calm_c * COVID_Stress1 + SEX + YINTERN
AL +
    Covid_Age, data = dt.covid))
```

```
##
## Call:
## lm(formula = SDQ_C_INT ~ RAmyg50.Fear_GT_Calm_c * COVID_Stress1 +
##     SEX + YINTERNAL + Covid_Age, data = dt.covid)
##
## Standardized Coefficients::
##                           (Intercept)               RAmyg50.Fear_GT_Calm_c
##                           0.000000000                          0.076588430
##                         COVID_Stress1                                  SEX
##                           0.456637890                          0.381762185
##                             YINTERNAL                            Covid_Age
##                           0.185033627                          0.072210971
## RAmyg50.Fear_GT_Calm_c:COVID_Stress1
##                           0.008374496
```

Sensitivity Analyses of associations between amygdala activation and internalizing problems controlling for mean framewise displacement and percentage of data censored due to motion outliers.

```
lamod1 <- summary(lm(SDQ_C_INT ~ LAmyg.Fear_GT_Calm + SEX + YINTERNAL + Covid
_Age +
    fd + outliers, data = dt.covid))
ramod1 <- summary(lm(SDQ_C_INT ~ RAmyg.Fear_GT_Calm + SEX + YINTERNAL + Covid
_Age +
    fd + outliers, data = dt.covid))
lamod1
```

```
##
## Call:
## lm(formula = SDQ_C_INT ~ LAmyg.Fear_GT_Calm + SEX + YINTERNAL +
##     Covid_Age + fd + outliers, data = dt.covid)
##
## Residuals:
##     Min      1Q  Median      3Q     Max
## -6.1858 -1.9656 -0.5221  1.3562 10.2635
##
## Coefficients:
##                     Estimate Std. Error t value Pr(>|t|)
## (Intercept)         -6.36877    9.02938  -0.705  0.48205
## LAmyg.Fear_GT_Calm -0.88383    0.32897  -2.687  0.00831 **
## SEX                  2.81210    0.61577   4.567 1.27e-05 ***
## YINTERNAL            3.16787    1.37365   2.306  0.02292 *
## Covid_Age            0.52318    0.62273   0.840  0.40260
```

```
## fd                   1.65395    3.06234   0.540  0.59020
## outliers             0.01134    0.10676   0.106  0.91563
## ---
## Signif. codes:  0 '***' 0.001 '**' 0.01 '*' 0.05 '.' 0.1 ' ' 1
##
## Residual standard error: 3.105 on 113 degrees of freedom
##   (25 observations deleted due to missingness)
## Multiple R-squared:  0.2242, Adjusted R-squared:  0.183
## F-statistic: 5.443 on 6 and 113 DF,  p-value: 5.622e-05
```

```
lm.beta(lm(SDQ_C_INT ~ LAmyg.Fear_GT_Calm + SEX + YINTERNAL + Covid_Age + fd
+ outliers,
    data = dt.covid))
```

```
##
## Call:
## lm(formula = SDQ_C_INT ~ LAmyg.Fear_GT_Calm + SEX + YINTERNAL +
##     Covid_Age + fd + outliers, data = dt.covid)
##
## Standardized Coefficients::
##        (Intercept) LAmyg.Fear_GT_Calm                SEX          YINTERNA
L
##         0.00000000        -0.22726418         0.40820267         0.2077271
7
##          Covid_Age                 fd           outliers
##         0.07023308         0.06608556         0.01300273
```

```
ramod1
```

```
##
## Call:
## lm(formula = SDQ_C_INT ~ RAmyg.Fear_GT_Calm + SEX + YINTERNAL +
##     Covid_Age + fd + outliers, data = dt.covid)
##
## Residuals:
##     Min      1Q  Median      3Q     Max
## -6.1609 -2.1694 -0.2048  1.3023 11.8849
##
## Coefficients:
##                    Estimate Std. Error t value Pr(>|t|)
## (Intercept)        -5.02815    9.28578  -0.541   0.5892
## RAmyg.Fear_GT_Calm -0.24744    0.34880  -0.709   0.4795
## SEX                 2.93952    0.63234   4.649 9.12e-06 ***
## YINTERNAL           3.09728    1.41444   2.190   0.0306 *
## Covid_Age           0.41605    0.64027   0.650   0.5171
## fd                  3.10014    3.14347   0.986   0.3261
## outliers           -0.03491    0.10854  -0.322   0.7483
## ---
## Signif. codes:  0 '***' 0.001 '**' 0.01 '*' 0.05 '.' 0.1 ' ' 1
##
## Residual standard error: 3.196 on 113 degrees of freedom
```

```
##   (25 observations deleted due to missingness)
## Multiple R-squared:  0.1783, Adjusted R-squared:  0.1347
## F-statistic: 4.087 on 6 and 113 DF,  p-value: 0.0009516
```

```
lm.beta(lm(SDQ_C_INT ~ RAmyg.Fear_GT_Calm + SEX + YINTERNAL + Covid_Age + fd
+ outliers,
    data = dt.covid))
```

```
##
## Call:
## lm(formula = SDQ_C_INT ~ RAmyg.Fear_GT_Calm + SEX + YINTERNAL +
##     Covid_Age + fd + outliers, data = dt.covid)
##
## Standardized Coefficients::
##        (Intercept) RAmyg.Fear_GT_Calm                SEX          YINTERNA
L
##         0.00000000        -0.06148649         0.42669844         0.2030982
3
##          Covid_Age                 fd           outliers
##         0.05585149         0.12387010        -0.04004681
```

```
laxmod1 <- summary(lm(SDQ_C_INT ~ LAmyg.Fear_GT_Calm_c * COVID_Stress1 + SEX
+ YINTERNAL +
    Covid_Age + fd + outliers, data = dt.covid))
raxmod1 <- summary(lm(SDQ_C_INT ~ RAmyg.Fear_GT_Calm_c * COVID_Stress1 + SEX
+ YINTERNAL +
    Covid_Age + fd + outliers, data = dt.covid))
laxmod1
```

```
##
## Call:
## lm(formula = SDQ_C_INT ~ LAmyg.Fear_GT_Calm_c * COVID_Stress1 +
##     SEX + YINTERNAL + Covid_Age + fd + outliers, data = dt.covid)
##
## Residuals:
##     Min      1Q  Median      3Q     Max
## -5.8429 -1.7021 -0.5304  1.3751  9.2435
##
## Coefficients:
##                                     Estimate Std. Error t value Pr(>|t|)
## (Intercept)                        -7.821659   7.964891  -0.982   0.3282
## LAmyg.Fear_GT_Calm_c               -0.506510   0.298985  -1.694   0.0931 .
## COVID_Stress1                       0.714757   0.148755   4.805 4.89e-06 *
**
## SEX                                 2.459154   0.546523   4.500 1.69e-05 *
**
## YINTERNAL                           2.483672   1.221077   2.034   0.0443 *
## Covid_Age                           0.624739   0.549081   1.138   0.2577
## fd                                  1.666559   2.700393   0.617   0.5384
## outliers                            0.005822   0.095164   0.061   0.9513
## LAmyg.Fear_GT_Calm_c:COVID_Stress1 -0.452082   0.177381  -2.549   0.0122 *
```

```
## ---
## Signif. codes:  0 '***' 0.001 '**' 0.01 '*' 0.05 '.' 0.1 ' ' 1
##
## Residual standard error: 2.736 on 111 degrees of freedom
##   (25 observations deleted due to missingness)
## Multiple R-squared:  0.4082, Adjusted R-squared:  0.3656
## F-statistic: 9.571 on 8 and 111 DF,  p-value: 5.273e-10
```

```
lm.beta(lm(SDQ_C_INT ~ LAmyg.Fear_GT_Calm_c * COVID_Stress1 + SEX + YINTERNAL
+ Covid_Age +
    fd + outliers, data = dt.covid))
```

```
##
## Call:
## lm(formula = SDQ_C_INT ~ LAmyg.Fear_GT_Calm_c * COVID_Stress1 +
##     SEX + YINTERNAL + Covid_Age + fd + outliers, data = dt.covid)
##
## Standardized Coefficients::
##                          (Intercept)                LAmyg.Fear_GT_Calm_c
##                           0.00000000                         -0.13024236
##                        COVID_Stress1                                 SEX
##                           0.36957632                          0.35696868
##                            YINTERNAL                           Covid_Age
##                           0.16286207                          0.08386692
##                                   fd                            outliers
##                           0.06658947                          0.00667837
## LAmyg.Fear_GT_Calm_c:COVID_Stress1
##                          -0.19269482
```

```
raxmod1
```

```
##
## Call:
## lm(formula = SDQ_C_INT ~ RAmyg.Fear_GT_Calm_c * COVID_Stress1 +
##     SEX + YINTERNAL + Covid_Age + fd + outliers, data = dt.covid)
##
## Residuals:
##    Min     1Q Median     3Q    Max
## -6.449 -1.703 -0.407  1.197  8.909
##
## Coefficients:
##                                     Estimate Std. Error t value Pr(>|t|)
## (Intercept)                        -6.696557   8.279692  -0.809   0.4204
## RAmyg.Fear_GT_Calm_c                0.222239   0.325786   0.682   0.4966
## COVID_Stress1                       0.862850   0.159419   5.412 3.63e-07 *
**
## SEX                                 2.605850   0.567986   4.588 1.18e-05 *
**
## YINTERNAL                           2.731808   1.259543   2.169   0.0322 *
## Covid_Age                           0.561315   0.571303   0.983   0.3280
## fd                                  2.273409   2.802372   0.811   0.4190
```

```
## outliers                           -0.052120   0.097092  -0.537   0.5925
## RAmyg.Fear_GT_Calm_c:COVID_Stress1 -0.005066   0.174147  -0.029   0.9768
## ---
## Signif. codes:  0 '***' 0.001 '**' 0.01 '*' 0.05 '.' 0.1 ' ' 1
##
## Residual standard error: 2.842 on 111 degrees of freedom
##   (25 observations deleted due to missingness)
## Multiple R-squared:  0.3617, Adjusted R-squared:  0.3156
## F-statistic: 7.861 on 8 and 111 DF,  p-value: 2.498e-08
```

```
lm.beta(lm(SDQ_C_INT ~ RAmyg.Fear_GT_Calm_c * COVID_Stress1 + SEX + YINTERNAL
+ Covid_Age +
    fd + outliers, data = dt.covid))
```

```
##
## Call:
## lm(formula = SDQ_C_INT ~ RAmyg.Fear_GT_Calm_c * COVID_Stress1 +
##     SEX + YINTERNAL + Covid_Age + fd + outliers, data = dt.covid)
##
## Standardized Coefficients::
##                        (Intercept)               RAmyg.Fear_GT_Calm_c
##                        0.000000000                        0.055224691
##                      COVID_Stress1                                SEX
##                        0.446149604                        0.378263026
##                          YINTERNAL                          Covid_Age
##                        0.179133177                        0.075352603
##                                 fd                           outliers
##                        0.090836917                       -0.059788514
## RAmyg.Fear_GT_Calm_c:COVID_Stress1
##                       -0.002345758
```
